# The wheat powdery mildew resistance gene *Pm4* also confers resistance to wheat blast

**DOI:** 10.1038/s41477-024-01718-8

**Published:** 2024-06-19

**Authors:** Tom O’Hara, Andrew Steed, Rachel Goddard, Kumar Gaurav, Sanu Arora, Jesús Quiroz-Chávez, Ricardo Ramírez-González, Roshani Badgami, David Gilbert, Javier Sánchez-Martín, Luzie Wingen, Cong Feng, Mei Jiang, Shifeng Cheng, Susanne Dreisigacker, Beat Keller, Brande B. H. Wulff, Cristóbal Uauy, Paul Nicholson

**Affiliations:** 1 John Innes Centre, Norwich Research Park, Norwich, UK; 2 Department of Plant and Microbial Biology, Zürich-Basel Plant Science Center, University of Zürich, Zürich, Switzerland; 3 Agricultural Genomics Institute at Shenzhen, Chinese Academy of Agricultural Sciences, Shenzhen, China; 4 International Maize and Wheat Improvement Centre (CIMMYT), Texcoco, Mexico; 5 Present Address: Department of Microbiology and Genetics, Spanish-Portuguese Agricultural Research Center (CIALE), University of Salamanca, Salamanca, Spain; 6 Present Address: Plant Science Program, Biological and Environmental Science and Engineering Division (BESE), King Abdullah University of Science and Technology (KAUST), Thuwal, Saudi Arabia; 7 Present Address: Center for Desert Agriculture, KAUST, Thuwal, Saudi Arabia

**Keywords:** Plant genetics, Plant breeding, Plant immunity

## Abstract

Wheat blast, caused by the fungus *Magnaporthe oryzae*, threatens global cereal production since its emergence in Brazil in 1985 and recently spread to Bangladesh and Zambia. Here we demonstrate that the *AVR-Rmg8* effector, common in wheat-infecting isolates, is recognized by the gene *Pm4*, previously shown to confer resistance to specific races of *Blumeria graminis* f. sp. *tritici*, the cause of powdery mildew of wheat. We show that *Pm4* alleles differ in their recognition of different *AVR-Rmg8* alleles, and some confer resistance only in seedling leaves but not spikes, making it important to select for those alleles that function in both tissues. This study has identified a gene recognizing an important virulence factor present in wheat blast isolates in Bangladesh and Zambia and represents an important first step towards developing durably resistant wheat cultivars for these regions.

## Abstract (web summary)

The wheat powdery mildew resistance gene *Pm4* also confers resistance to wheat blast carrying the effector *AVR-Rmg8*. The authors propose the *Pm4f* allele as the most effective allele to deploy in Bangladesh and Zambia.

## Main

Wheat blast was first identified in Brazil in 1985 (ref. ^1^) and then spread to neighbouring countries in South America before appearing in Bangladesh in 2016 (ref. ^2^) and Zambia in 2018 (ref. ^3^). Wheat blast is caused by the fungus *Magnaporthe oryzae* (synonym of *Pyricularia oryzae*) pathotype *triticum* (MoT) and is considered to pose a threat to major wheat (*Triticum aestivum*) producers including India and China, where conditions are conducive to this disease. Pathotypes of *M. oryzae* show high levels of host specificity, and only six resistances have been reported to be effective against MoT pathotypes in wheat^4^ (Supplementary Table 1). These include *Rmg7*, *Rmg8* and the 2NS translocation originating from *Aegilops ventricosa* that has been introgressed onto the short arm of wheat chromosome 2A (ref. ^4^). Like most race-specific resistances, *Rmg7*, *Rmg8* and 2NS can give complete resistance depending upon the environment and genetic background^5,6^.

Genome-wide association studies of field-based resistance to wheat blast revealed that the 2NS resistance was the only potent and robust resistance in international field trials^7–9^. Unfortunately, some MoT isolates are virulent on wheat varieties carrying the 2NS translocation, and this resistance is not effective in all genetic backgrounds^5,10^ making it important to identify additional sources of resistance. *Rmg7* was identified in a tetraploid wheat accession (*Triticum dicoccum*, KU120)^11^ and mapped to chromosome 2A, while *Rmg8* was identified in hexaploid wheat line S-615 and mapped to chromosome 2B^12^. While *Rmg8* remains effective at higher temperatures, *Rmg7* loses its ability to confer resistance^6^. It was later demonstrated that *Rmg7* and *Rmg8* both recognize the same effector *AVR-Rmg8*^6^. Despite mapping these loci, no resistance gene effective against MoT has been cloned to date.

It has recently been reported that wheat blast isolates from Bangladesh and Zambia are part of a clonal lineage termed B71^13^. It was shown that members of this lineage all carry *AVR-Rmg8*, making identification of the corresponding resistance gene(s) an important target. We previously identified two wheat resistance genes, *Rwt3* and *Rwt4*, that acted as host specificity barriers against non-MoT pathotypes using isolates carrying specific effectors to screen a panel of wheat accessions^14^. *Rwt3* encodes a conventional nucleotide-binding domain leucine-rich repeat (NLR), and *Rwt4* encodes a wheat tandem kinase. In this Article, we used *k*-mer-based association of a large, whole genome shotgun-sequenced wheat diversity panel to identify the wheat gene recognizing and providing resistance to isolates of MoT carrying *AVR-Rmg8*.

## Results

### Resistance to *AVR-Rmg8* maps to a 5.3 Mbp region on chromosome 2A of wheat

We selected two MoT isolates to phenotype resistance to *AVR-Rmg8*. The first isolate (Py 15.1.018) carries the *eI* allele of *AVR-Rmg8* and is virulent against cultivar Jagger and the CBFusarium ENT014 wheat line. (Supplementary Fig. 1), despite them both carrying the 2NS resistance^15,16^. The second isolate (NO6047 + *AVR-Rmg8*) (henceforth referred to as NO6047 + *AVR8*) is a derivative of isolate NO6047 that was transformed with allele *eI* of *AVR-Rmg8* under control of the *PWL2* promoter^17^ (Supplementary Table 2). NO6047 contains an alternative allele of the *AVR-Rmg8* effector, designated *eII*′′′, which is not recognized by *Rmg8*^13^. This makes NO6047 an ideal isolate to host the effector *AVR-Rmg8*. Isolate NO6047 is virulent on wheat line S-615, which carries *Rmg8*, whereas isolate NO6047 + *AVR8* is not virulent because of host recognition of *AVR-Rmg8*^17^. The resistance of wheat accessions showing resistance to NO6047 + *AVR8* but not to NO6047 were assumed to be due to recognition of the *AVR-Rmg8* effector.

**Fig. 1:**
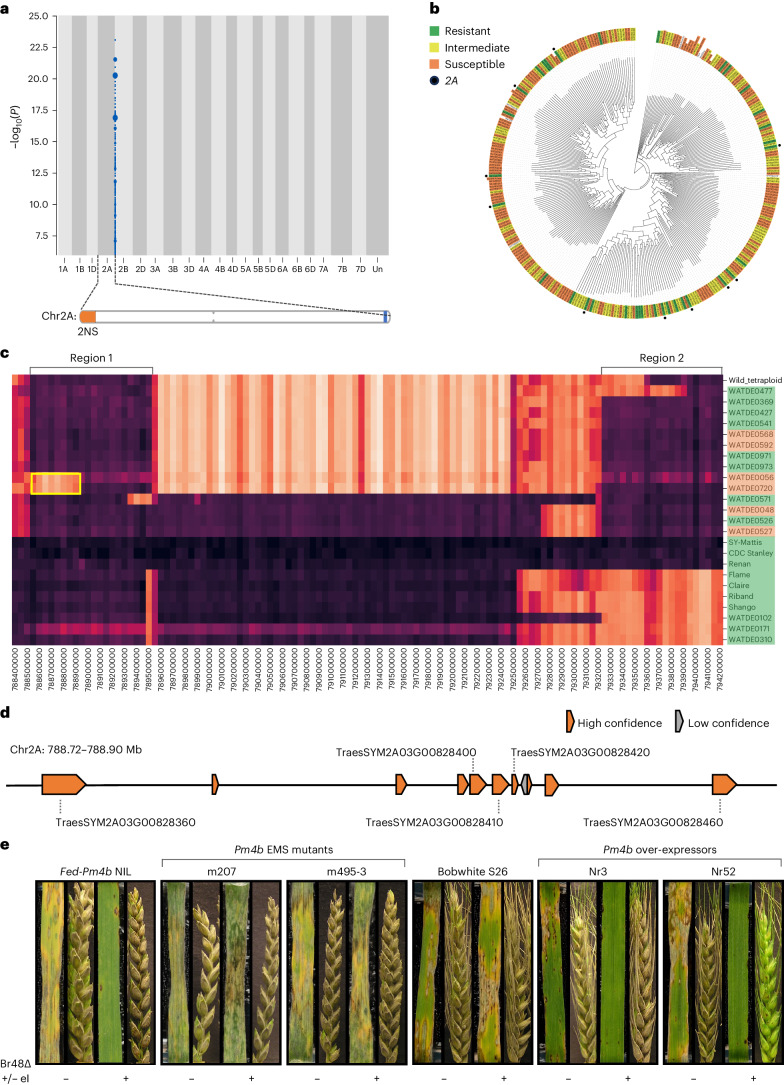
Genetic identification and validation of resistance to the wheat blast fungus effector *AVR-Rmg8* by *k*-mer-based association mapping and haplotype analysis. **a**, *k*-mers (WATDE0310) associated with resistance to Py 15.1.018 mapped to the SY-Mattis genome. Points on the *y* axis depict *k*-mers positively associated with resistance in blue. Point size is proportional to the number of *k*-mers. The association score is defined as the −log_10_ of the *P* value obtained using the likelihood ratio test for nested models (two-sided). The ideogram shows the position of the *A. ventricosa* 2NS segment (orange) and the AgRenSeq 2A association (blue) on the distal ends of the short and long chromosome arms, respectively. **b**, *k*-mer-based phylogeny of wheat landraces showing the phenotype of an accession after inoculation with Py 15.1.018. The phenotype of an accession after inoculation is indicated by the colour used to highlight the label of that accession (green, resistant (scores less than or equal to 3); yellow, intermediate (scores more than 3, less than 5) and orange, susceptible (scores equal to or greater than 5). Black circles indicate the presence of the chromosome 2A peak based on the AgRenSeq association plots. **c**, Representative cluster heat map for the haplotypes within the chromosome 2A interval using SY-Mattis as the reference. The phenotype of an accession after inoculation with Py 15.1.018 is indicated by the colour used to highlight the label of that accession, as in **b**. The darker the colour within a 50 kb window the more identical by state that sequence is to SY-Mattis. Among the accessions carrying the chromosome 2A interval, two regions were particularly similar to SY-Mattis, region 1 (788,550,000 to 789,550,000) and region 2 (793,250,000 to 794,250,000). Note that the region 1 haplotype block extends approximately 250 kb upstream of the 5.3 Mb 2A interval. Flame, Claire, Riband, Shango, WATDE0102, WATDE0171 and WATDE0310 were resistant but only contained region 1. WATDE0056 and WATDE0720 were susceptible and lacked the first 400 kb of region 1, highlighted in a yellow box. Source data are available in ref. ^69^. **d**, Gene content of the 400 kb in Mattis according to the de novo gene models. Genes coloured orange and grey correspond to high- and low-confidence genes, respectively. Genes that show expression in total leaf tissue at the three-leaf stage are labelled with their gene codes. **e**, Wheat blast detached leaf and spike assays for the *Pm4b* EMS-induced mutants of *Fed-Pm4b* and *Pm4b* over-expressors in the Bobwhite S26 background. Leaves and spikes were inoculated with *M. oryzae* isolates Br48ΔeI and Br48ΔeI+eI at 22 °C, denoted by ‘−’ and ‘+’, respectively.

We screened seedlings of a panel of 320 wheat lines including 300 landraces from the A. E. Watkins collection (Wingen et al.^18^) and wheat lines with chromosome-scale assemblies^15^ using the two MoT isolates described above (Supplementary Table 3). Only 13 accessions were highly resistant to the NO6047 + *AVR8* isolate (score of 1.5 or less) (Supplementary Table 4 and Supplementary Fig. 2), only 10 accessions were highly resistant to Py 15.1.018 (score of 1.5 or less) and 9 of these were also highly resistant to both isolates suggesting that, while resistance is rare, the majority of the resistance observed was due to the recognition of the same effector in the two isolates (Fig. 1b, Supplementary Table 4 and Supplementary Fig. 3). The accessions that conferred resistance to only NO6047 + *AVR8* likely contain an additional resistance that recognizes an effector absent in Py 15.1.018. Of the cultivars with chromosome or scaffold-scale assemblies, three (SY-Mattis, CDC Stanley, Claire) were highly resistant to both isolates (but susceptible to the wild-type isolate NO6047) (Supplementary Figs. 4 and 5). The resistance of these three cultivars enables them to be used as references for subsequent analyses to locate and identify the causal gene.

The NO6047 + *AVR8* Watkins phenotype data were analysed using NLR-enriched *k*-mer based association genetics (henceforth referred to as AgRenSeq^14^) with SY-Mattis as the reference genome. This produced a clear association peak on chromosome arm 2AL, spanning 788.8 Mbp to 794.1 Mbp. The Py 15.1.018 leaf disease scores were also analysed using SY-Mattis as the reference and produced an identical association peak (Fig. 1a). Ten Watkins accessions highly resistant to Py 15.1.018 produced associations within the same interval on chromosome arm 2AL using SY-Mattis as the reference (Fig. 1a, Supplementary Figs. 6 and 7 and Supplementary Table 4). These data mapped the resistance to *AVR*-*Rmg8* to a 5.3 Mbp chromosome 2A interval on SY-Mattis.

### Interrogating the 5.3 Mbp chromosome 2A interval

Haplotype analysis was run across the genomic sequence of the *AVR*-*Rmg8* resistance interval in the full Watkins collection (827 accessions) and a selection of modern wheat varieties (218 cultivars) using SY-Mattis as the reference genome^14,19^. A cluster heat map with 50 kb window size was generated to identify regions identical or near-identical to SY-Mattis which revealed that an additional 20 accessions of the Watkins collection carry sections of the 5.3 Mbp interval. Within the 5.3 Mbp interval, two 1 Mbp blocks of similarity among 63 accessions were observed, ‘region 1’ (788,550,000 bp to 789,550,000 bp) and ‘region 2’ (793,250,000 bp to 794,250,000 bp) (Fig. 1c). These 20 Watkins accessions, along with an additional 10 lines that lack the *AVR*-*Rmg8* resistance interval, were phenotyped with isolates NO6047 + *AVR8* and Py 15.1.018 (Supplementary Table 5). A cluster heat map containing the 20 additional Watkins lines is shown in Supplementary Fig. 8. Interestingly, the wild tetraploid wheat accession 33255 was highly similar to all of region 1 and to 600 kb of region 2, indicating that the interval may have originated from a hybridization between hexaploid wheat with a wheat wild relative similar to *Triticum turgidum* (Fig. 1c and Supplementary Fig. 8)^15^. Five Watkins lines (WATDE0102, WATDE0171, WATDE0310, WATDE0566 and WATDE0804) contained only region 1 but showed resistance, suggesting that the *AVR*-*Rmg8* resistance was contained within this interval (Fig. 1c). In addition, 22 modern European wheat cultivars also possessed only the region 1 SY-Mattis haplotype (Supplementary Fig. 8). These 22 cultivars were all resistant to both NO6047 + *AVR8* and Py 15.1.018 isolates (Supplementary Table 6) confirming that the *AVR*-*Rmg8* resistance is within the 1 Mbp interval termed region 1. Among the Watkins lines containing region 1, two lines (WATDE0056 and WATDE0720) both lacked the proximal 400 kb of region 1, and both were susceptible to NO6047 + *AVR8* and Py 15.1.018 isolates indicating that the *AVR*-*Rmg8* resistance lies within this 400 kb region (788550000–788950000 bp).

The 400 kb *AVR*-*Rmg8* resistance interval within region 1 contains ten annotated genes (Fig. 1d and Supplementary Table 7), only five of which were expressed in RNA sequencing (RNA-seq) data from total leaf tissue at the three-leaf stage (Supplementary Table 8). Notably, four lines (WATDE0048, WATDE0527, WATDE0568 and WATDE0592) classified as containing region 1 were susceptible to both isolates. We therefore compared the sequences of the five expressed genes between the four susceptible lines and resistant lines using the whole genome Watkins sequencing data and SY-Mattis as the reference^19^. Two of the genes (TraesSYM2A03G00828410 and TraesSYM2A03G00828450) were monomorphic among susceptible and resistant lines, and two (TraesSYM2A03G00828400 and TraesSYM2A03G00828460) had polymorphisms which did not associate with the resistance phenotype (Supplementary Table 9). In the remaining gene (TraesSYM2A03G00828360), two of the susceptible accessions (WATDE0568 and WATDE0592) contained a T/A single-nucleotide polymorphism (SNP) converting amino acid 446 from tryptophan to a stop codon (W446*) (Supplementary Table 10), while the other two susceptible accessions (WATDE0048 and WATDE0527) possessed an identical G/A SNP converting amino acid 50 from alanine to glutamic acid (A50E). The sequences of the five non-expressed genes in the interval were also examined, and no polymorphisms that segregated with resistance were identified. Thus, the combined haplotype and allelic diversity analyses identified TraesSYM2A03G00828360 as a strong candidate gene for recognizing and conferring resistance to isolates of MoT carrying *AVR-Rmg8*.

### The powdery mildew resistance gene *Pm4* confers resistance to MoT

The RNA-seq data revealed that TraesSYM2A03G00828360 is alternatively spliced resulting in two potential transcripts. The intron/exon structure for the first five exons was the same in both transcripts, while the last exons were distinct. Transcript 1 produced a protein of 560 amino acids, while in transcript 2 the fifth intron extended an additional 1,082 bp (encapsulating the sixth exon from transcript 1) and produced a 747-amino-acid protein. BLAST analysis of transcript 1 revealed it to be almost identical to the previously reported chimeric protein of a serine/threonine kinase and multiple C2 domains and transmembrane regions that function as the wheat powdery mildew (*Blumeria graminis* f. sp. *tritici* (*Bgt*)) race-specific resistance gene *Pm4*^20^. This study also established that *Pm4* has alternate splicing, producing ‘isoforms’ Pm4b-V1 (560 amino acids) and Pm4b-V2 (747 amino acids), corresponding to TraesSYM2A03G00828360 transcript 1 and transcript 2, respectively. Both isoforms are required to confer resistance to wheat mildew^20^. This suggests that the wheat blast *AVR*-*Rmg8* resistance is encoded by *Pm4*.

To confirm recognition of *AVR-Rmg8* by *Pm4*, we used the germplasm resources previously developed to characterize its role in resistance to powdery mildew. This included near-isogenic lines (NILs) for two functionally distinct *Pm4* alleles (*Pm4a* and *Pm4b*) in the susceptible wheat cultivar Federation (*Fed-Pm4a*, *Fed-Pm4b*), *Pm4b* Ethyl methanesulfonate (EMS)-induced mutants in *Fed-Pm4b*, and transgenic lines of susceptible cultivar Bobwhite S26 overexpressing *Pm4b* (Supplementary Table 11)^20^. To relate differences in response specifically to the presence or absence of the *eI* allele of *AVR-Rmg8*, we inoculated this germplasm with isogenic transformants of MoT isolate Br48 differing in the presence of only *AVR-Rmg8*. Isolate Br48∆eI has been disrupted to remove *AVR-Rmg8 eI*, while this gene has been replaced in isolate Br48∆eI+eI^21^. Federation was susceptible to both Br48∆eI and Br48∆eI+eI, while *Fed-Pm4b* and *Fed-Pm4a* (carrying different *Pm4* alleles) were both resistant in seedling leaves to Br48∆eI+eI (Fig. 1e and Supplementary Fig. 9). All three lines were susceptible in spikes inoculated and incubated at 22 °C to both Br48∆eI and Br48∆eI+eI indicating that these alleles (*Pm4a* and *Pm4b*) only function in seedling resistance (Fig. 1e and Supplementary Fig. 9). Loss of wheat blast resistance in adult plants of many wheat varieties has been observed previously, but the reasons for tissue- or stage-specific resistance is unknown^22^.

All eight loss-of-function EMS-induced mutants of *Fed-Pm4b* were susceptible to both MoT isolates in seedling assays (Fig. 1e and Supplementary Figs. 9 and 10). Mutations were present in exon 6 and 7 specific to *Pm4b_V1* and *Pm4b_V2*, respectively, indicating that both transcripts are required for resistance to MoT as was found to be the case for *Bgt*^20^. While Bobwhite S26 and lines S#3 and S#52 segregating from the T_1_ plants but lacking the transgene were susceptible to Br48∆eI and Br48∆eI+eI in seedling assays, both *Pm4b* over-expressing lines (Nr#3 and Nr#52) carrying the full-length complementary DNAs of *Pm4b_V1* and *Pm4b_V2* were resistant to Br48∆eI+eI. Surprisingly, Nr#52 was resistant to Br48∆eI+eI in spikes, while Nr#3 was susceptible (Fig. 1e). Sánchez-Martín et al.^20^ report that Nr#3 contains single copies of *Pm4b_V1* and *Pm4b_V2*, while Nr#52 contains two or more copies of the transcripts. Expression of *Pm4b_V1* and *Pm4b_V2* was assessed in spike tissues of Nr#3 and Nr#52. Expression of *Pm4b_V1* was significantly higher in Nr#52 compared to Nr#3 (*P* < 0.001) indicating that overexpression is sufficient to confer recognition and resistance in spike tissues to MoT isolates carrying *AVR-Rmg8* (Supplementary Fig. 11). The requirement for multiple copies or high expression of genes to provide full resistance has been reported recently^23^, suggesting that increased copy number, or expression levels, may provide a route to increase disease resistance.

#### Allelic variation

We designed PCR-based assays to detect *Pm4* and used these to investigate its prevalence in landraces and modern adapted varieties (primers ‘P1_F_hex’, ‘P1_F_fam’ and ‘P1_COM’ detailed in Supplementary Table 12). These primers do not differentiate between the different *Pm4* alleles. We found *Pm4* to be uncommon among the landraces within the Watkins collection, being present in only 28 of 827 (3.4%) accessions. The proportion of *Pm4*-containing varieties was higher (15.5%; 67 out of 432) in the ‘Gediflux’ collection of highly successful European varieties from the period 1945–2000 (ref. ^18^) (Supplementary Table 13). This probably reflects the selection of *Pm4* by breeders in Europe to control mildew while this disease is of lesser importance in many other parts of the globe.

**Fig. 2:**
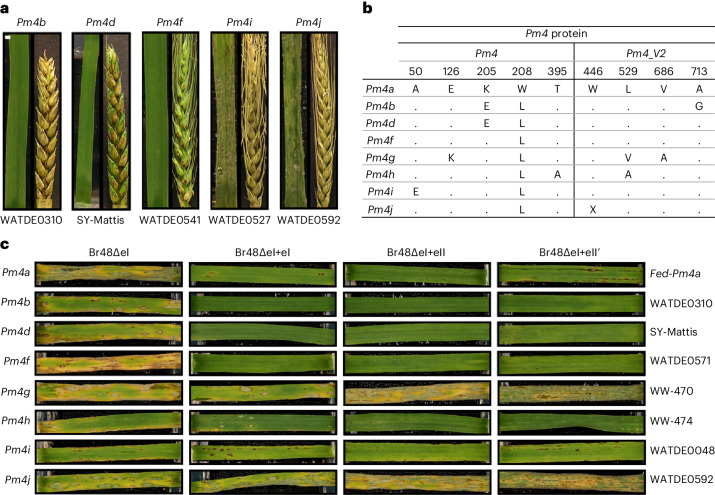
The effect of allelic variation in *Pm4* and *AVR-Rmg8* on wheat blast symptoms*.* **a**, Representative wheat blast detached leaf and spike assays for the *Pm4b*, *Pm4d*, *Pm4f*, *Pm4i* and *Pm4j* alleles, inoculated with Py 15.1.018 at 22 °C. **b**, Protein sequence comparison of the known *Pm4* alleles. Dots represent the same amino acid present in *Pm4a*. **c**, Representative wheat blast detached leaf assays for the known *Pm4* alleles, inoculated with *M. oryzae* isolates Br48ΔeI, Br48ΔeI+eI, Br48ΔeI+eII and Br48ΔeI+eII′ at 22 °C.

An allelic series of *Pm4*, each recognizing different isolates of *Bgt*, has been reported, and many of these originate from wild relatives of *T. aestivum*^20^. *Pm4b*/*Pm4c* share 100% nucleotide sequence identity, as do *Pm4d*/*Pm4e*, and are henceforth referred to as *Pm4b* and *Pm4d*, respectively^24^. *Pm4a* and *Pm4b* were introduced from tetraploid wheats^25,26^, while *Pm4d* is believed to have been introgressed from *Triticum monococcum*^27^. The origins of *Pm4f*, *Pm4g* and *Pm4h* are unknown. The two alleles of *Pm4* identified within this study (A50E and W446*) had sequences most similar to *Pm4f* but have not been reported previously. We designated these as *Pm4i* and *Pm4j*, respectively (Fig. 2b). *Pm4b* was the most common *Pm4* allele among the *Pm4*-containing modern wheat varieties included in the haplotype analysis that were genotyped (71%), but it was rare among the Watkins collection (11%). By contrast, *Pm4f* was not observed in the modern varieties but was present in 21 of the 28 Watkins collection accessions containing *Pm4* (Supplementary Table 14). *Pm4d* was absent within the Watkins collection and was only found in combination with the 2NS segment (33 Mbp, ref. ^28^) on the short arm of chromosome 2A introgressed from *A. ventricosa* into the wheat cultivar VPM1^29^ (Supplementary Table 14). The 2NS segment on the short arm of chromosome 2A carries the rust resistance genes *Sr38*, *Yr17* and *Lr37* along with resistance to isolates of MoT^5^. It has been proposed that *Pm4* was introduced into the long arm of chromosome 2A from the *Triticum persicum* parent of VPM1^30^. The absence of *Pm4d* in Watkins accessions and its presence alongside the 2NS segment from *A. ventricosa* in modern varieties supports this view and indicates that the *Pm4d* allele may have been introduced only once into *T. aestivum* through VPM1 at the same time as 2NS but at the opposite end of the 2A chromosome and from a different wheat relative. This represents a second example of the serendipitous introduction of resistance into wheat from VPM1 as this line was originally developed to introduce the *Pch1* eyespot resistance gene on chromosome 7D^v^ of *A. ventricosa* into wheat^31^, and the presence of the 2NS on the end of the short arm of chromosome 2A was not recognized.

The efficacy of seedling and spike resistance against *AVR-Rmg8* (isolate Py 15.1.018) was compared at 22 °C and 26 °C across wheat accessions carrying different *Pm4* alleles as it has been reported that resistance to MoT is often temperature sensitive^6^. Carriers of *Pm4b*, *Pm4d* and *Pm4f* were all resistant at the seedling stage at both temperatures (Fig. 2a and Supplementary Fig. 12). These three *Pm4* alleles, however, differed in efficacy in spikes. Carriers of *Pm4d* and *Pm4f* were resistant at 22 °C, while *Pm4b* carriers were susceptible confirming the ineffectiveness of the *Pm4b* allele observed in the *Fed-Pm4b* NIL (Figs. 1e and 2a). The level of expression of the *V1* and *V2* transcripts of *Pm4b* and *Pm4f* in spikes were not significantly different among the wheat varieties examined (*P* ≥ 0.314 and *P* ≥ 0.750 or *V1* and *V2* transcripts, respectively) indicating that differences in resistance more probably reflect differences in interaction between host and pathogen components than differences in expression of *Pm4* (Supplementary Fig. 11). Carriers of *Pm4d* expressed moderate resistance in the spikes at 26 °C, while carriers of *Pm4b* and *Pm4f* were susceptible at this temperature. It should be noted that all the carriers of *Pm4d* also contained the 2NS segment that functions only in spike tissues^5^, and the resistance may reflect the presence of the two resistances in these varieties.

### The effectiveness of *Pm4* alleles against alleles of *AVR-Rmg8*

Three alleles of *AVR-Rmg8* (*eI*, *eII* and *eII*′) were identified among MoT isolates collected in Brazil with *eII* being predominant^32^. The clonal lineage present in Bangladesh and Zambia, however, contains the *eI* allele^21^. Isolates transformed to carry different alleles of *AVR-Rmg8* (*eI*, *eII* and *eII*′) differed in aggressiveness towards a wheat line (IL191) carrying *Rmg8*, with resistance being more pronounced against isolates carrying *eI* than those carrying *eII* or *eII*′ (ref. ^21^).

The relative effectiveness of *Pm4* alleles against different alleles of *AVR-Rmg8* was examined by screening seedlings of wheat lines carrying different *Pm4* alleles for resistance to transformants lacking *AVR-Rmg8* or carrying *eI*, *eII* or *eII*′ alleles (Supplementary Table 15)^21^. The majority of *Pm4* alleles conferred resistance to all three *AVR-Rmg8* effector alleles: *Pm4a*, *Pm4b*, *Pm4d*, *Pm4f*, *Pm4h* and *Pm4i* (Fig. 2c). Alleles *Pm4g* (accession WW-470^33^) and *Pm4j* did not confer resistance against any of the three *AVR-Rmg8* effector alleles. The lack of effectiveness of *Pm4j* was expected as this protein is truncated and *Pm4g* was previously reported to be a susceptible *Pm4* allele with respect to resistance to *Bgt*^20^. Interestingly, the same study also reported *Pm4f* to be a *Bgt-*susceptible allele, but it is effective against the three alleles of *AVR-Rmg8*. *Pm4a*, *Pm4b*, *Pm4d* and *Pm4h* (accession WW-474, ref. ^33^) are also highly effective against the three alleles of *AVR-Rmg8*. The two accessions carrying *Pm4i* (WATDE0048 and WATDE0527) showed greater resistance against Br48 carrying the *eI* or *eII* allele of *AVR-Rmg8* than against the same isolate carrying the *eII*′ effector allele (Fig. 2c and Supplementary Table 15). A comparison of resistance responses of the different *Pm4* alleles against selected MoT and *Bgt* isolates is shown in Supplementary Table 16. Differences in aggressiveness of isolates carrying different *AVR-Rmg8* alleles has also been noted previously^21^. These two accessions, however, were highly susceptible to Py 15.1.018 (*eI*) and NO6047 + *AVR8* (*eI* and *eII*′′) (Fig. 2a and Supplementary Fig. 13). We postulate that this may be due to the presence of additional effectors in these isolates that suppress *Pm4i* alleles in an equivalent manner to that reported for *PWT4*^34^.

**Fig. 3:**
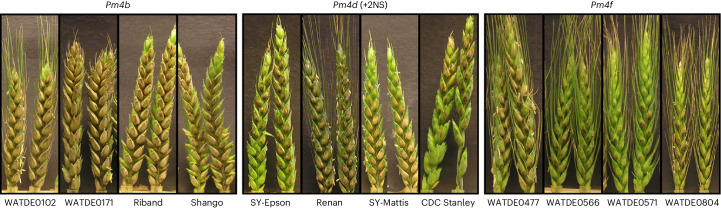
Identifying whether *Pm4* functions in the spike against Bangladeshi wheat blast isolates. Wheat blast detached spike assays for *Pm4b*, *Pm4d* and *Pm4f* alleles inoculated with Bangladeshi isolate BTJ4P-1 at 22 °C.

The pandemic clonal lineage of MoT present in Bangladesh and Zambia contains the *eI* allele of *AVR-Rmg8*^13^, and so it was important to demonstrate whether *Pm4* alleles would function against this lineage. Furthermore, as the impacts of MoT infection are most dramatic for spike disease, we screened spikes of wheat accessions carrying different *Pm4* alleles for resistance to the Bangladesh isolate BTJP4-1^13^. As anticipated from the studies above, accessions carrying *Pm4b* did not show resistance to BTJP4-1 in spikes. By contrast, spikes of accessions carrying *Pm4f* were highly resistant to this isolate (Fig. 3). Spikes of accessions carrying *Pm4d* showed moderate resistance to BTJP4-1, but this probably reflects the presence of the 2NS segment in all these accessions. Assessment of the International Maize and Wheat Improvement Center(CIMMYT)’s international screening nurseries has revealed that the 2NS segment contributes almost all the wheat blast resistance present within both the Bread Wheat Screening Nurseries and the Semi-Arid Wheat Screening Nurseries^8^. These authors emphasized the urgent need to identify additional non-2NS sources of resistance. We believe that the *Pm4f* allele provides such a source.

## Discussion

No resistance gene against MoT has been cloned to date. In this study, we used isolates carrying the *eI* allele of *AVR-Rmg8* to screen a genome-sequenced diversity panel of hexaploid wheat and clone a resistance gene recognizing this effector. Surprisingly, this gene, encoding a serine–threonine kinase–Multiple C2 domain and transmembrane region (MCTP) protein, has previously been identified as *Pm4*, a race-specific resistance gene to wheat powdery mildew^20^. *Bgt* is an obligate biotropic pathogen of wheat that exclusively infects epidermal cells^35^. By contrast, *Mo* is a hemibiotroph that can colonize all aerial tissues^36^. Further work is required to understand how a single gene can recognize two distinct pathogens with different life cycles. Despite emerging less than 40 years ago, MoT arose via an opportunistic cross between *Urochloa* and *Eleusine* infecting lineages and represents recombination of standing variation present in these pathotypes^37^. Thus, *AVR-Rmg8* was present in the progenitors of MoT that would have provided the selection pressure to maintain *Pm4* to provide resistance in wheat. This is the second example of resistance to blast and mildew being conferred by the same gene. The NLR mildew resistance gene *MLA3* of barley also recognizes the effector *PWL2* that acts as a host range determinant preventing *M. oryzae* from infecting weeping lovegrass (*Eragrostis curvula*)^38^. Furthermore, *Pm24*, an allele of the tandem kinase *Rwt4* that recognizes *PWT4*, an effector present in many non-*Triticum* pathotypes of *M. oryzae*^14^, also confers resistance against powdery mildew of wheat^39^. *Pm4* and *Pm24* are both examples of non-canonical *R-*genes encoding kinase fusion proteins^40^. The stem rust resistance gene *Sr62* from *Aegilops sharonensis* has been identified as an orthologue of *Pm24*/*Rwt4*^41^. A small number of genes of different classes have been shown to confer resistance to multiple pathogens in wheat or its progenitors. These include the NLRs *Mla7* and *Mla8*^42^ and the receptor-like kinase *TuRLK1*^43^, which confer resistance to wheat powdery mildew and wheat yellow rust, in addition to NLRs *Lr548* and *Yr548*, which confer resistance to leaf and stripe rust, respectively^44^.

The different alleles of *Pm4* can be split into three categories: effective against both MoT and *Bgt* (*Pm4a*, *Pm4b*, *Pm4d* and *Pm4h*), ineffective against both MoT and *Bgt* (*Pm4g*) and effective against MoT but ineffective against *Bgt* (*Pm4f*) (Supplementary Table 16). *Pm4i* and *Pm4j* are effective against MoT but have not been tested against *Bgt*. As *Pm4j* contains a truncated protein, it is likely also ineffective against *Bgt*. Asuke et al.^45^ propose that *Rmg8* on 2BL corresponds to *Pm4f* and that the *Rmg7* locus on 2AL consists of *Pm4a*, *Pm4b* and *Pm4d*. The predominant *Pm4* allele (*Pm4f*) identified within the Watkins collection functions against wheat blast in both seedling and spike tissues but was not found in elite European varieties, highlighting the potential value of landrace materials as sources of resistance. *Pm4* was not found in a selection of 565 CIMMYT wheat breeding lines carrying the 2NS translocation originating from wheat line VPM1 (Supplementary Table 17). As VPM1 was also the donor of *Pm4d* into wheat, this suggests that *Pm4d* was lost in the breeding process, probably because mildew resistance is not a major breeding target for CIMMYT target environments. These findings are supported by genome-wide association studies of wheat blast resistance in CIMMYT’s international screening nurseries in Bangladesh and Bolivia where the only robust and potent resistance was associated with 2NS, with no resistance identified in the *Pm4* region^8,9^. This emphasizes the importance of maintaining selection for as wide a range of diseases as practical because of the potential for unexpected benefits of resistances effective against multiple pathogens^38,42,43^. Conventionally, the search for new resistances centres upon examining accessions originating from regions where the disease is believed to have originated on the assumption that co-evolution of host and pathogen will have led to selection for resistance. This approach is not possible with wheat blast as this disease first appeared in 1985. The finding that at least some resistances effective against powdery mildew in wheat also confer resistance to wheat blast suggests that the search for additional resistances might benefit from focus on non-obvious regions with cool, damp environments where wheat mildew is most problematic. Our data support previous studies that concluded *Pm4* may have originated in wild tetraploid species in which several distinct alleles were identified^20,26^. Extending analysis to additional tetraploid wild wheat species may reveal new *Pm4* alleles with greater efficacies against the different alleles of *AVR-Rmg8* and so improve resistance against wheat blast in Bangladesh, Zambia and South America. It is important to maintain *Rwt4* within breeding programmes for blast resistance as *Pwt4* suppresses resistance provided by *Rmg8* in *rwt4* germplasm^34^.

The urgent need to identify additional wheat blast resistance sources to complement and protect against loss of efficacy of the 2NS resistance is widely recognized^8,46^. Our results show that *Pm4* offers the first such resistance and provides an important entry point to identify additional resistances. This work also raises new questions as to whether other mildew or rust resistances also confer resistance against wheat blast and suggests the need for additional research to establish the basis of similarity in resistances against *P. oryzae*, *B. graminis* and *Puccinia* species through comparison of effectors, resistance genes and their interactions. Further work is also required to establish whether the *Pm4*-associated leaf and spike resistance observed at 26 °C in this study is maintained at the higher temperatures often reached in Bangladesh and Zambia^47,48^. Even if spike resistance was lost, leaf resistance at high temperatures would still be highly advantageous to breeding efforts as conidia production on leaves has been shown to contribute to spike infection^49,50^.

## Methods

### Phenotyping plant material with wheat blast isolates

The MoT isolate Py 15.1.018 and the transformed isolates Br48∆eI and Br48∆eI+eI^21^ were grown on complete medium agar. The MoT isolate NO6047 and the transformed isolate NO6047+*AVR*8 were grown on oatmeal media. Oatmeal media was prepared by adding powdered oats (40 g) to 500 ml of distilled H_2_O and placed in a water bath at 65 °C for 1 h and then filtered through two layers of muslin. As much liquid was extracted from the oats as possible before dividing equally between two 1 l Schott bottles. Agar (10 g) (Sigma Aldrich) and 2.5 g sucrose were added to each bottle, and the volume was made up to 500 ml before autoclaving. Fungal inoculum was prepared as described by Goddard et al.^51^. A conidial suspension of 0.2 × 10^6^ to 0.4 × 10^6^ conidia per millilitre was used for all inoculations. Seedling and spike assays were carried out as described by Goddard et al.^51^ and scored for disease symptoms using a 0–6 scale as described by Arora et al.^14^. Five and three biological replicates were used for seedling and spike assays, respectively. Detached tissue *Mo* infection assays give comparable disease responses to whole plant assays^52,53^.

#### *k*-mer-based association mapping

Association mapping was performed using similar methods to Arora et al.^14^ using the reference genome SY-Mattis^15^.

### Haplotype analysis

Haplotype analysis was used to refine the chromosome 2A AgRenSeq candidate region. In brief, a visualization cluster heat map using the ‘variations’ database described in https://github.com/Uauy-Lab/IBSpy was used to identify candidates sharing similarity with the SY-Mattis genome reference within the chromosome 2A interval. The complete variations data are available in ref. ^54^. Samples with similar variations profiles in the target region were selected to run short reads alignments against the SY-Mattis reference (ref. ^15^). Alignments for the 10 Watkins accessions with the chromosome 2A AgRenSeq association, in addition to 10 adapted varieties and the 20 Watkins accessions outside of the core panel identified as having the chromosome 2A interval, were generated. The alignments were produced using bowtie2 (v.2.4.1) (Supplementary Tables 9 and 10)^19,55^. Alignments in SAM format were processed using samtools (v.1.7)^56^ and transformed to BAM format removing duplicates and filtering for mapping quality (MAPQ) > 30. BAM files were visualized with IGV (v.2.8.0)^57^ to detect candidate SNPs within the 400 kb *Rmg8* interval.

### Genome annotations and RNA-seq analysis

Pre-publication access was granted to the SY-Mattis RNA-seq data and gene annotations (v.03G) generated by the 10+ Wheat Genome project. Note that the current public annotation on EnsemblPlants is v.01G, but the gene models discussed in this study are available in ref. ^58^.

Seedlings were grown in a controlled environment room (Conviron BDW80; Conviron) set at a 16 h day and 8 h night photoperiod, temperatures of 20 and 16 °C, respectively, and 60% relative humidity. Plants were sampled at the three-leaf stage (Zadocks GS13), during which whole aerial organs were collected separately 4 h after dawn (9:00). Each biological replicate consisted of a single plant. RNA sequence data from two biological replicates were mapped to the SY-Mattis reference genome using the Linux programme HISAT2 (v.2.1.0)^15,59^. BAM files were viewed using IGV (v.2.8.0)^57^.

### KASP marker design to detect *Pm4*

The wheat reference genome Chinese Spring^60^ lacks *Pm4* (ref. ^20^). A region in exon 7 differentiating the functional *Pm4* from its closest (non-functional) homeologue in Chinese Spring, within gene TraesCS2A01G557900, was used to design kompetitive allele-specific PCR (KASP) markers (LGC genomics) to distinguish *Pm4*/*pm4* carriers in wheat (Supplementary Table 12)^20^. The KASP markers were validated on genome-sequenced cultivars and a subset of Watkins and adapted lines (Walkowiak et al.^15^) and could detect all known alleles of *Pm4*. Subsequently, KASP marker analysis was performed on the Gediflux Collection (497) to understand the distribution of *Pm4* within northern European wheat (Supplementary Table 13). Sixty-seven accessions were identified as containing a *Pm4* allele. All reactions were run using the following touchdown PCR programme using an Eppendorf vapo.protect Mastercycler pro 384 (Eppendorf AG, 22331): 94 °C for 15 min, 94 °C for 20 s followed by 65 °C for 1 min (repeated ten times, decreasing by 0.8 °C each cycle to 57 °C), 94 °C for 20 s followed by 57 °C for 1 min (repeated 30 times) and held at 16 °C. An additional five to ten cycles were sometimes required for full separation of the signals from the different genotypes. Plates were read using a PHERAstar microplate reader (BMG LABTECH) and analysed using the genotyping data analysis software KlusterCaller (v.4.1.1.23135).

### Quantitative real-time PCR

Expression of *Pm4b_V1* and *Pm4b_V2* in the spike was quantified through reverse transcription, quantitative real-time PCR (Supplementary Fig. 11). Mature spikes were harvested and individually shock frozen in liquid nitrogen and stored at −70 °C. Three biological replicates were sampled per genotype. RNA was extracted using RNeasy Plant Mini Kit (74904, Qiagen) according to the manufacturer’s protocol. Immediately after extraction, the samples were purified using DNA Turbo DNA-free kit (01134216, Invitrogen) according to the manufacturer’s protocol. Samples were quantified using a NanoDrop spectrophotometer (Thermo Scientific) and stored at −70 °C. First-strand cDNA was synthesized using SuperScript III First-Strand Synthesis System for RT-PCR kit (18080-051, Life Technologies). 1 μl each of 50 μM oligo(dT)_20_ and 50 ng μl^−1^ random hexamers were included in the first-strand cDNA synthesis. Subsequent steps were carried out according to the manufacturer’s protocol. Samples were quantified using a NanoDrop spectrophotometer and stored at −20 °C. Quantitative real-time PCR was performed with 4 μl of tenfold diluted cDNA in technical duplicates using SYBR Green JumpStart Taq ReadyMix (S4438, Sigma-Aldrich) as described by Chen et al.^61^. All reactions were run using a CFX96 Real-Time system C1000 Thermal Cycler (BioRad). Thermocycling conditions were 95 °C for 4 min, followed by 40 cycles of 94 °C for 10 s, then 60 °C for 10 s, then 72 °C for 30 s. Amplification specificity was confirmed using the ‘melting curve’ capability. Reference genes ADP and ZFL were used, as described by Sánchez-Martín et al.^20^. Average amplification efficiencies for the ADP, ZFL, *Pm4b_V1* and *Pm4b_V2* primers (Supplementary Table 12) were determined using a serial dilution (tenfold dilution decreasing to a 100,000-fold dilution) of a pool of six cDNA samples, to produce a standard curve. Target specific amplication efficiencies were calculated using the Agilent BioCalculator (https://www.agilent.com/store/bioCalcs.jsp#) and are given in Supplementary Table 18. Data are presented as the expression ratio of the target gene to the reference gene as described by Chen et al.^62^. Statistical analysis was performed using Genstat (v.22.1)^63^. Generalized linear model analysis using ‘replicate’ and ‘line’ was performed to compare relative expression of *V1* and *V2* in Nr52 and Nr3. A two-sample *t*-test was used to compare relative expression of *V1* and *V2* in *Pm4b* and *Pm4f* alleles at *P* < 0.05.

### Reporting summary

Further information on research design is available in the Nature Portfolio Reporting Summary linked to this article.

### Supplementary information

Supplementary Information Supplementary Figs. 1–13, Tables 1–18 and Refs.

Reporting Summary

## Data Availability

The *k*-mer matrix is available via Zenodo at 10.5281/zenodo.5557564 (ref. ^64^), The CLC assemblies of the 300 Watkins and 21 non-Watkins accessions are available via Zenodo at 10.5281/zenodo.5557685 (ref. ^65^), 10.5281/zenodo.5557721 (ref. ^66^), 10.5281/zenodo.5557827 (ref. ^67^), 10.5281/zenodo.5557837 (ref. ^68^). Sequencing data were obtained from Sequence Read Archive (SRA) accession SRX9897426 (Wild_tetraploid) and from the National Genomics Data Center (NGDC) Genome Sequence Archive (GSA) BioProject accession number PRJCA019636. Source data for Fig. 1c are available via Zenodo at 10.5281/zenodo.8377152 (ref. ^69^). The complete variations data used for haplotype analysis are available via Zenodo at 10.5281/zenodo.8355991 (ref. ^54^). SY-Mattis gene models discussed in this study are available via Zenodo at 10.5281/zenodo.8380858 (ref. ^58^). SY-Mattis genome data are available at https://ftp.ensemblgenomes.ebi.ac.uk/pub/plants/release-58/fasta/triticum_aestivum_mattis/.

## Code availability

Scripts for the Watkins *k*-mer matrix generation, phylogenetic tree construction and *k*-mer based association mapping are available via GitHub at https://github.com/arorasanu/watkins_renseq. Scripts for the haplotype analysis are available via GitHub at https://github.com/Uauy-Lab/IBSpy.
