# Supplementary material for: The wheat powdery mildew resistance gene Pm4 also confers resistance to wheat blast
Source: Nat Plants. 2024 Jun 19;10(6):984–93. doi: 10.1038/s41477-024-01718-8 (PMC11208137; doi:10.1038/s41477-024-01718-8)
Supplement: Supplementary file 1 — Supplementary Figs. 1–13, Tables 1–18 and Refs. [file 41477_2024_1718_MOESM1_ESM.pdf]

nature plants

Supplementary information https://doi.org/10.1038/s41477-024-01718-8

The wheat powdery mildew resistance gene *Pm4* also confers resistance to wheat blast

In the format provided by the authors and unedited

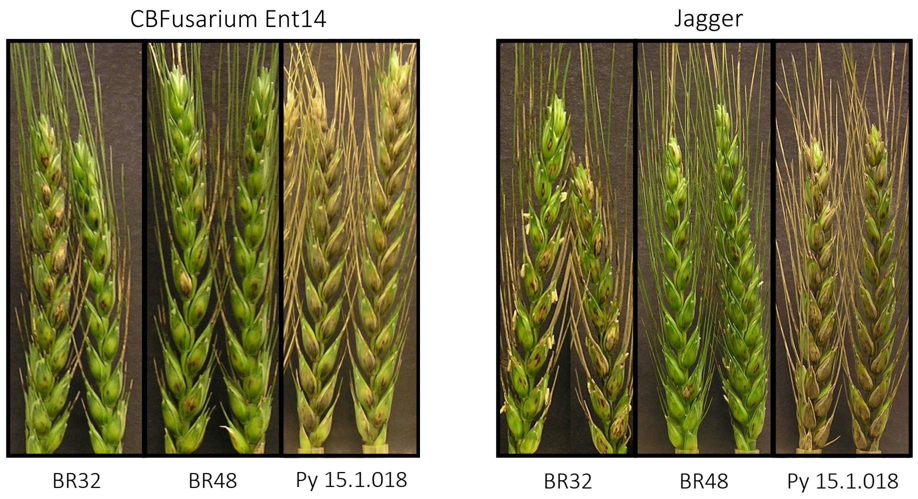

Figure S1 - Wheat blast detached spike assays for *Ae. ventricosa* 2NS translocation containing cultivars CBFusarium Ent14 and Jagger. Spikes were inoculated with Brazilian isolates BR32, BR48 and Py 15.1.018 at 22 ºC. Images were taken at six and seven days post inoculation for Jagger and CB Fusarium Ent14, respectively.

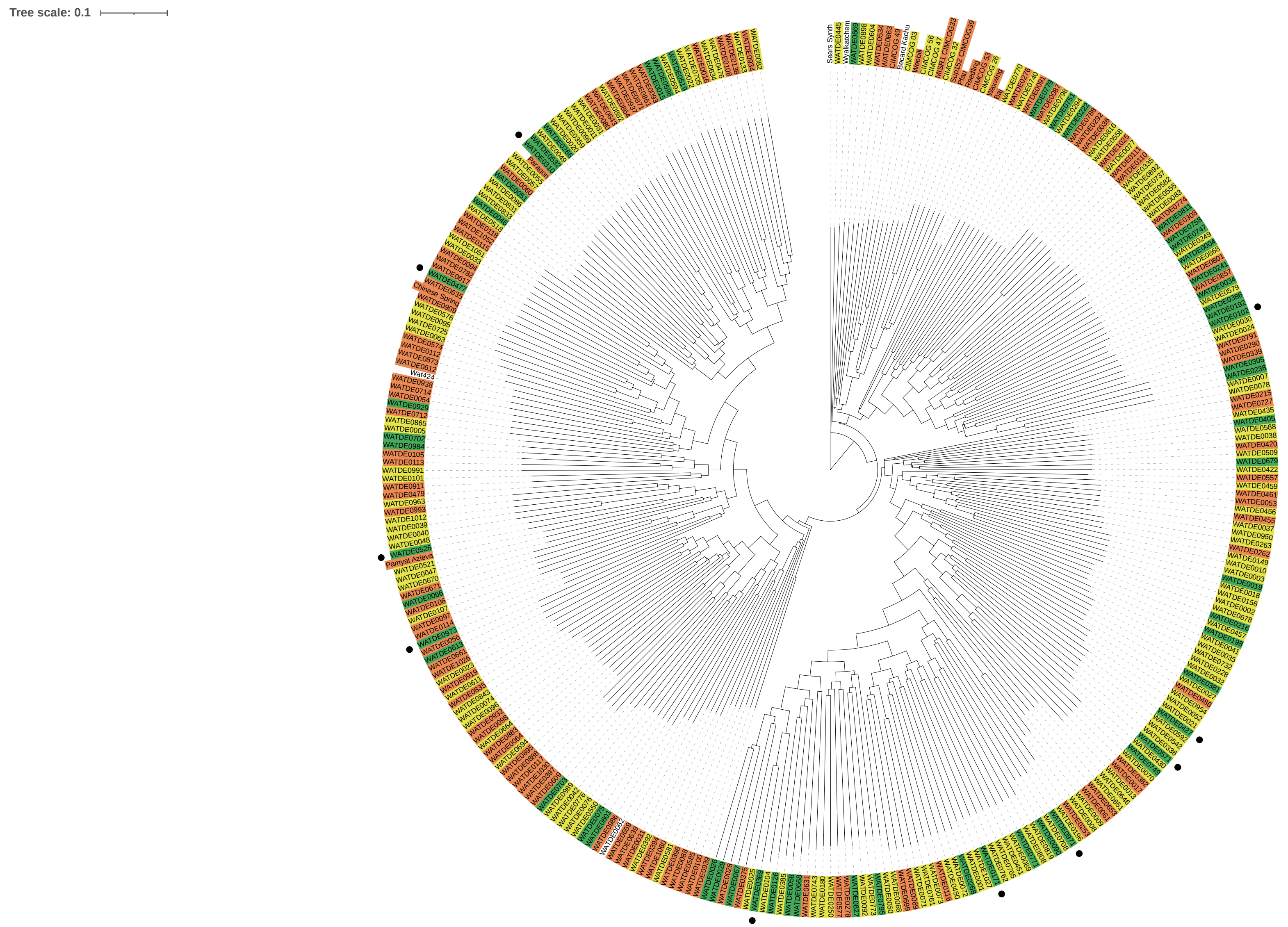

Figure S2 - *k*-mer-based phylogeny of wheat landraces showing the phenotype of an accession after inoculation with NO6047+*AVR8.* Phenotype of an accession after inoculation is indicated by the colour used to highlight the label of that accession (green = resistant (scores less than or equal to 3), yellow = intermediate (scores more than 3, less than 5) and orange = susceptible (scores equal to greater than 5). Black circles indicate the presence of the chromosome 2A peak based on the AgRenSeq association plots.

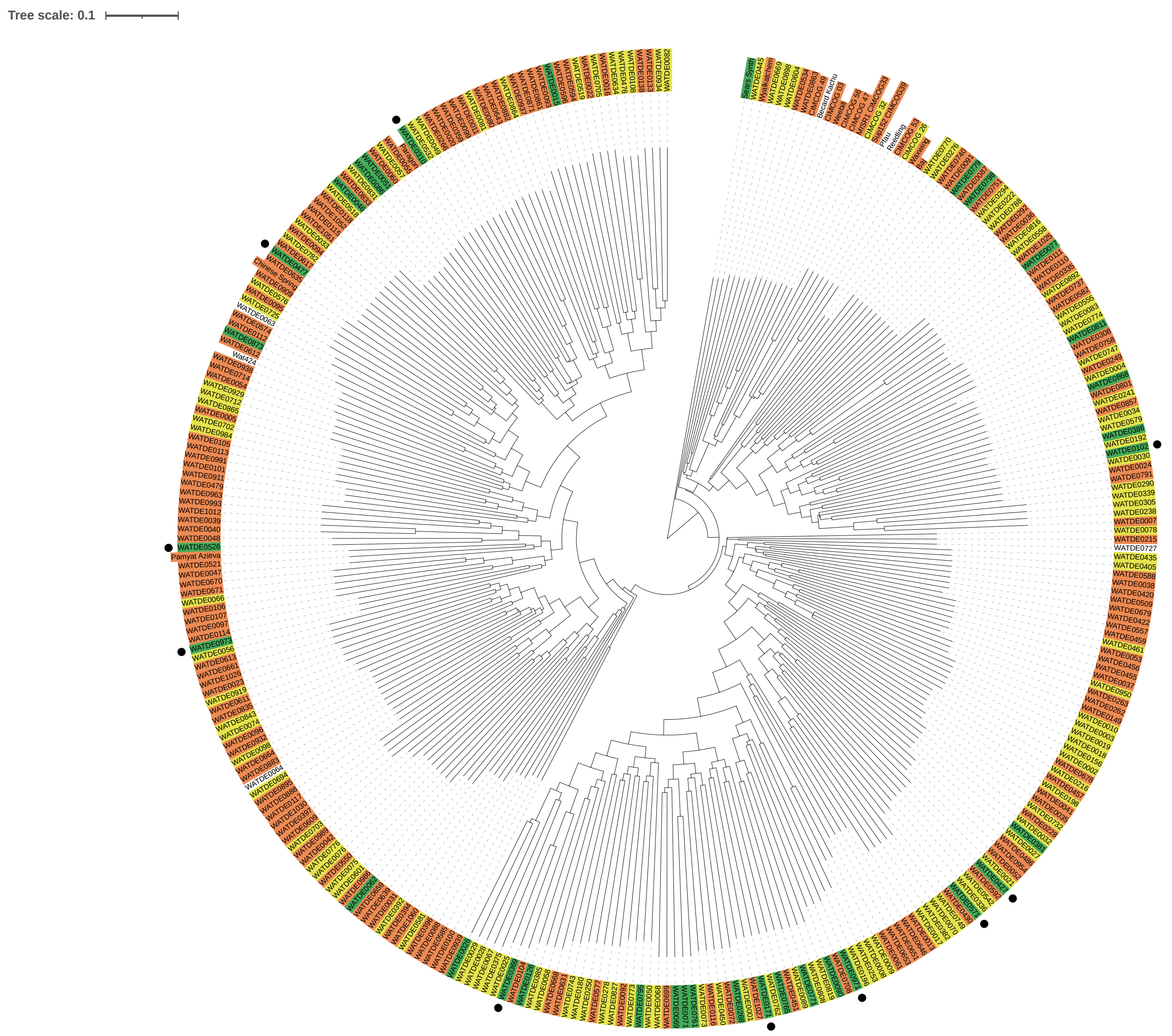

Figure S3 - *k*-mer-based phylogeny of wheat landraces showing the phenotype of an accession after inoculation with Py 15.1.018. Phenotype of an accession after inoculation is indicated by the colour used to highlight the label of that accession (green = resistant (scores less than or equal to 3), yellow = intermediate (scores more than 3, less than 5) and orange = susceptible (scores equal to greater than 5). Black circles indicate the presence of the chromosome 2A peak based on the AgRenSeq association plots.

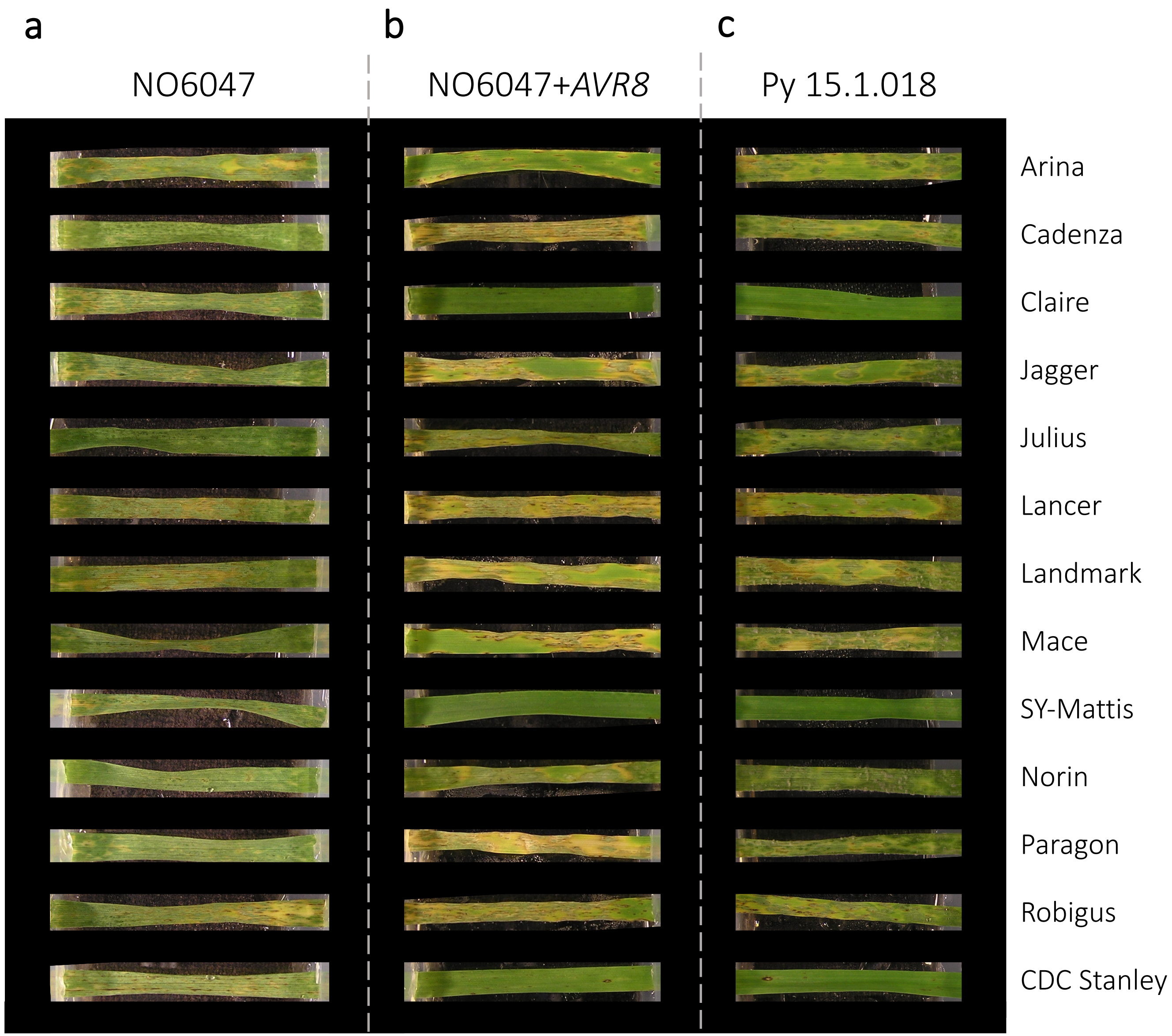

Figure S4 - Representative leaves of the 10 wheat cultivars with chromosome or scaffold-scale level assemblies used in this study. Leaves were inoculated at 22 °C with **a**. NO6047, **b**. NO6047+*AVR8* and **c**. Py 15.1.018. Images were taken at five days post inoculation.

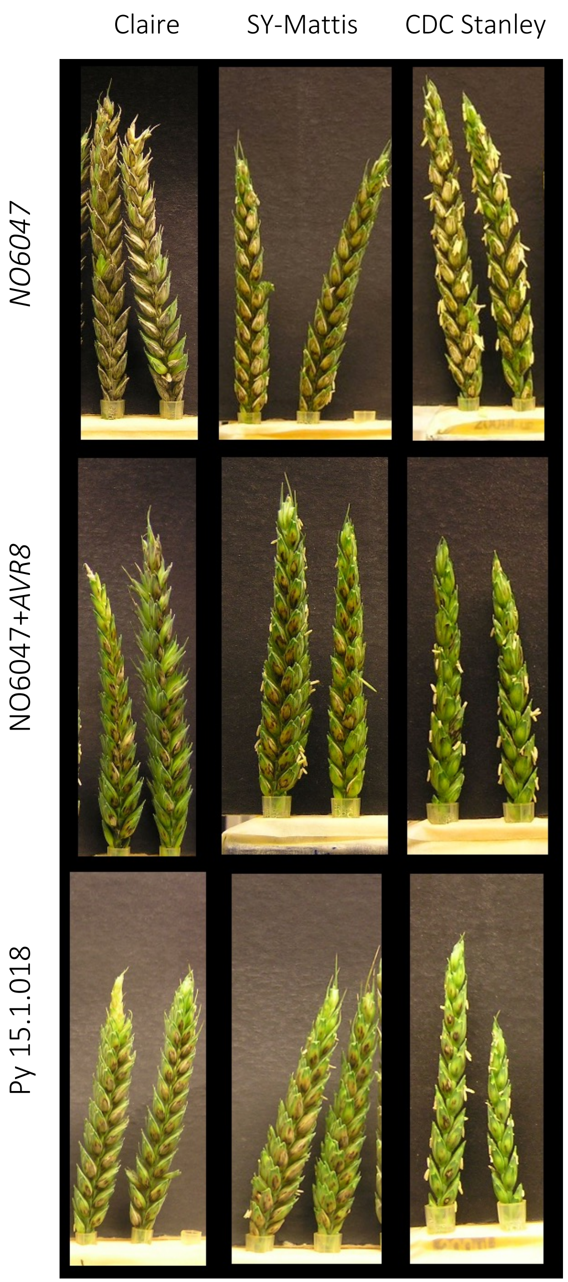

Figure S5 – Comparison of detached spike phenotypes for Claire, SY-Mattis and CDC Stanley. Spikes were inoculated at 22 °C with NO6047, NO6047+*AVR8* and Py 15.1.018. Images were taken at five to seven days post inoculation.

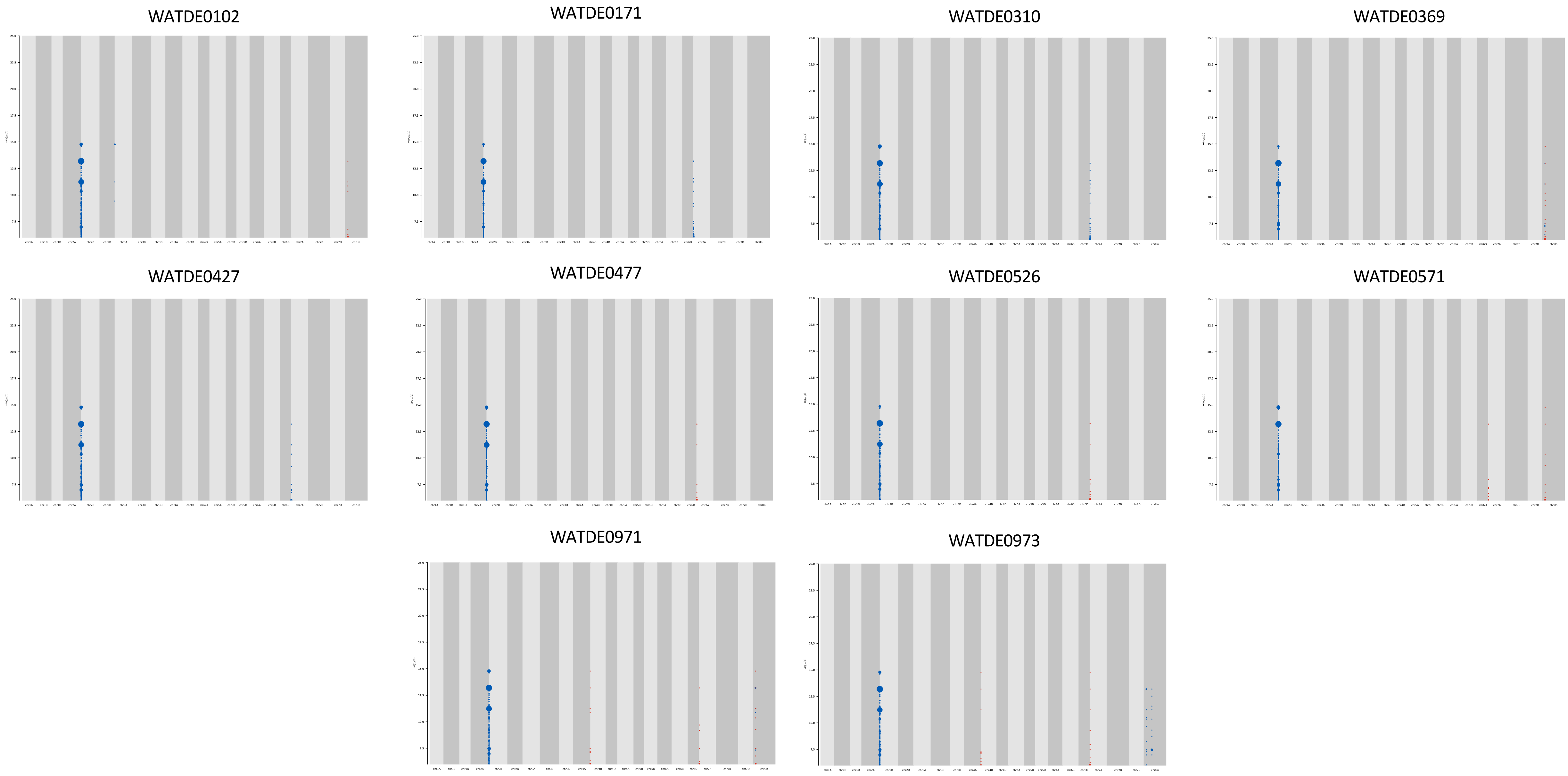

Figure S6 - *k*-mers associated with resistance to NO6047+*AVR8* mapped to SY-Mattis for the *k*-mer compliment of the ten Watkins accessions that have the chromosome 2A association peak. Phenotype data was collected at five days post inoculation. Points on the *y* axis depict *k*-mers positively associated with resistance in blue, and negatively associated with resistance in red. Point size is proportional to the number of *k*-mers. The association score is defined as the $-\log_{10}$ of the *P* value obtained using the likelihood ratio test for nested models (two-sided).

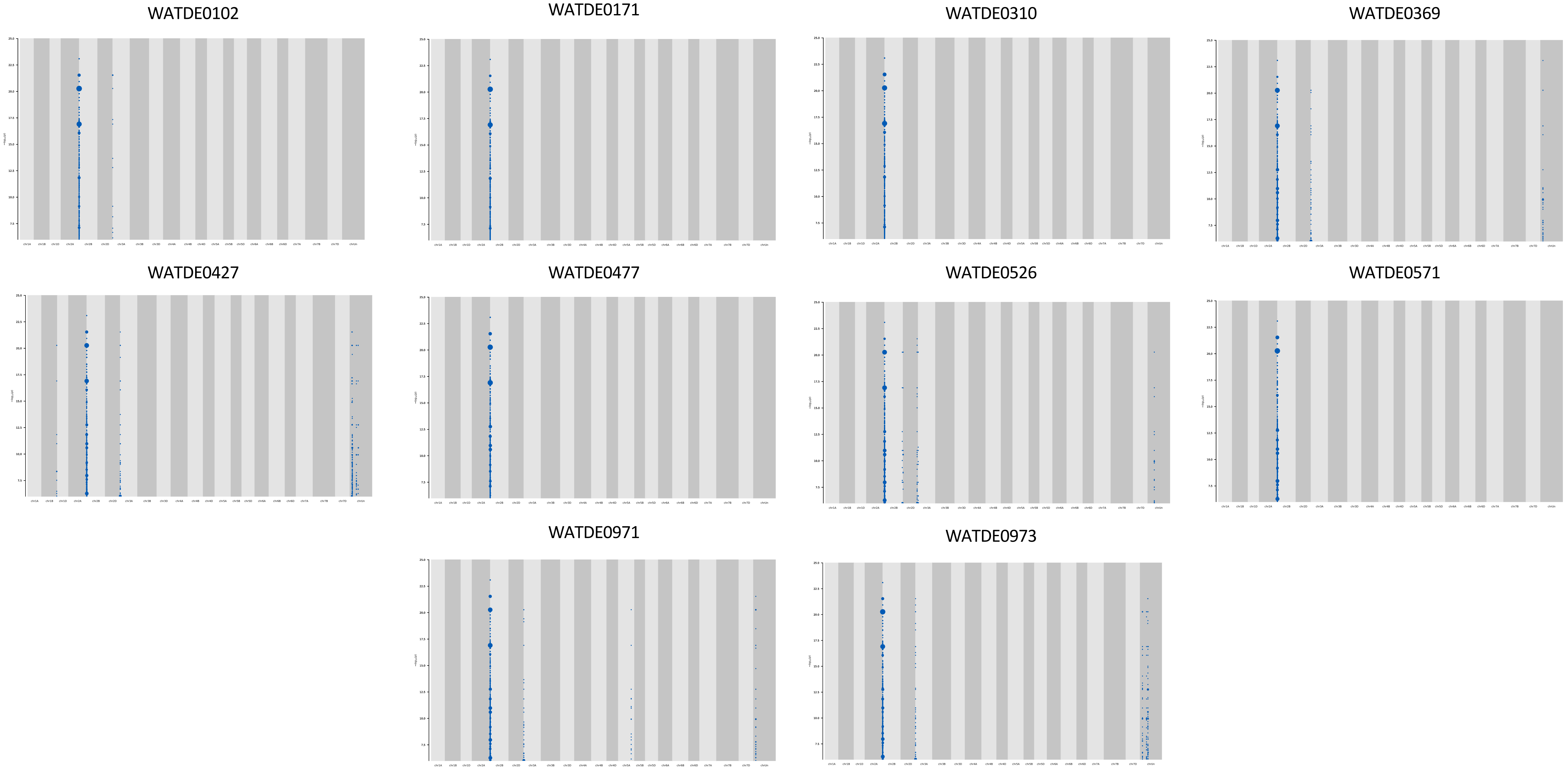

Figure S7 - *k*-mers associated with resistance to Py 15.1.018 mapped to SY-Mattis for the *k*-mer compliment of the ten Watkins accessions that have the chromosome 2A association peak. Phenotype data was collected at five days post inoculation. Points on the *y* axis depict *k*-mers positively associated with resistance in blue, and negatively associated with resistance in red. Point size is proportional to the number of *k*-mers. The association score is defined as the $-\log_{10}$ of the *P* value obtained using the likelihood ratio test for nested models (two-sided).

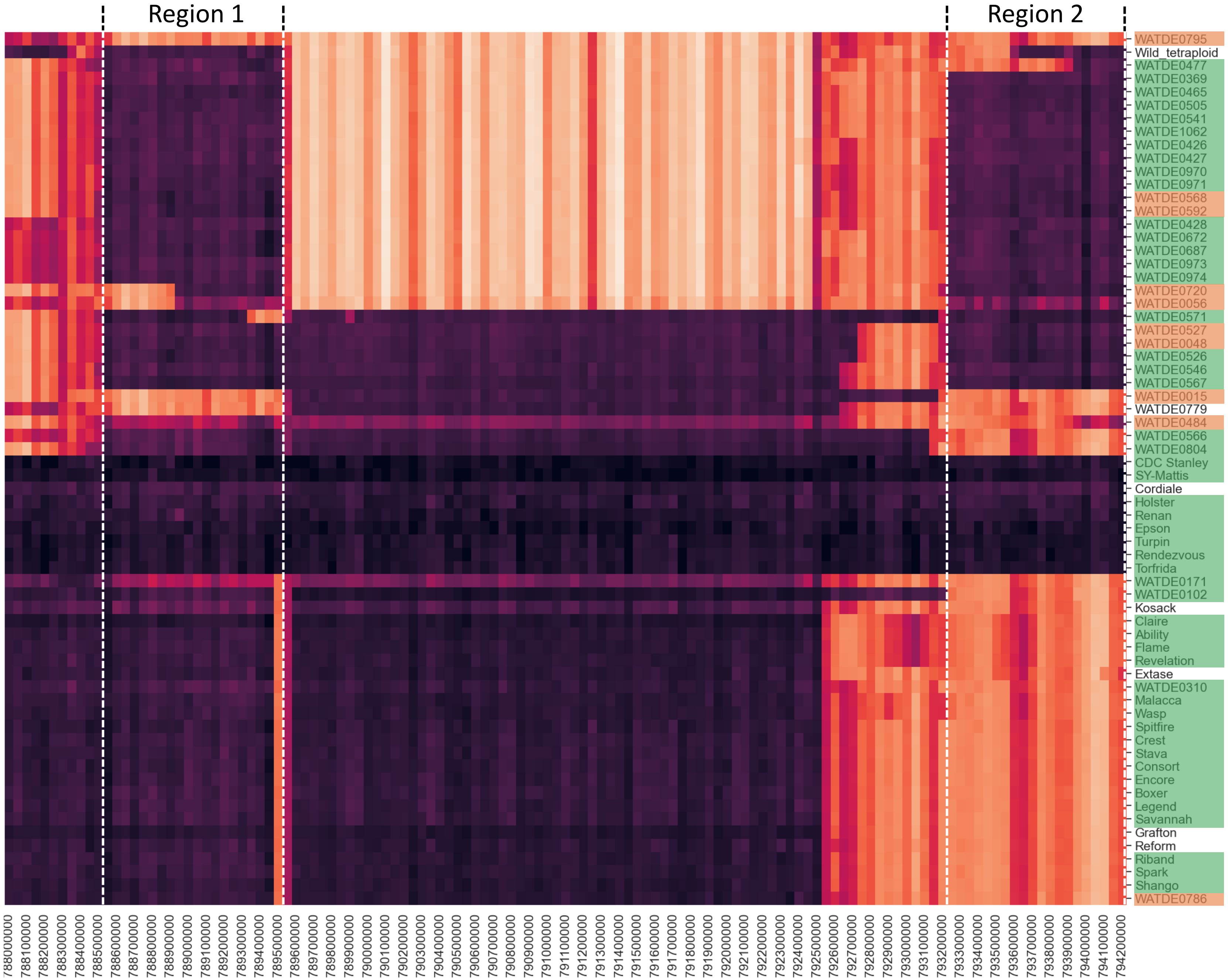

Figure S8 - Haplotype cluster heatmap for the chromosome 2A interval using SY-Mattis as the reference. Phenotype of an accession after inoculation with Py 15.1.018 is indicated by the colour used to highlight the label of that accession, (green = resistant (scores less than or equal to 3), yellow = intermediate (scores more than 3, less than 5) and orange = susceptible (scores equal to greater than 5)). The darker the colour within a 50 kb window, the more sequence identity that sequence has to SY-Mattis. Two blocks of similarity were observed, 'Region 1' (788,550,000 to 789,550,000) and 'Region 2' (793,250,000 to 794,250,000). Note that the 'Region 1' haplotype block extends approximately 250 kb upstream of the 5.3 Mb chromosome 2A interval (788.8 to 794.1 Mbp). The variations data are available from Zenodo under the DOI zenodo.org/record/8377192.

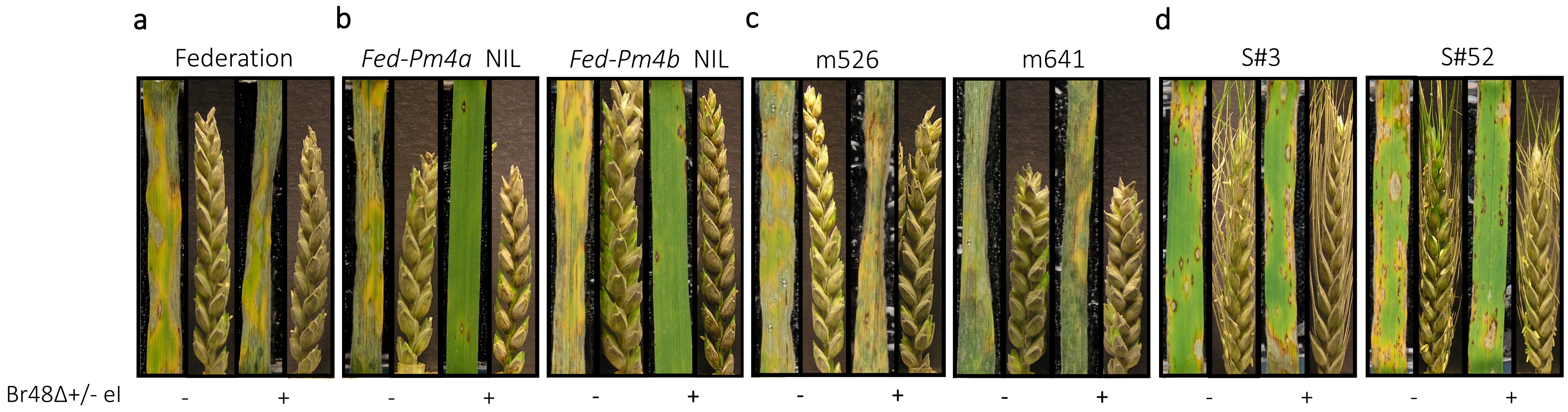

Figure S9 - Wheat blast detached leaf and spike assays for **a**, the susceptible genetic background Federation(Fed). **b**, *Fed-Pm4a* and *Fed-Pm4b* NILs. **c**, *Pm4b* EMS-induced mutants of *Fed-Pm4b* NIL. Leaves and spikes were inoculated with Br48ΔeI and Br48ΔeI+eI, denoted by '-' and '+', respectively. **d**, Sister lines originating from the same T_0 as the transgenic *Pm4b* over-expressors in the Bobwhite S26 background that have lost the transgene through segregation, S#3 and S#52 are sister lines to Nr#3 and Nr#52, respectively (Fig. 1e).

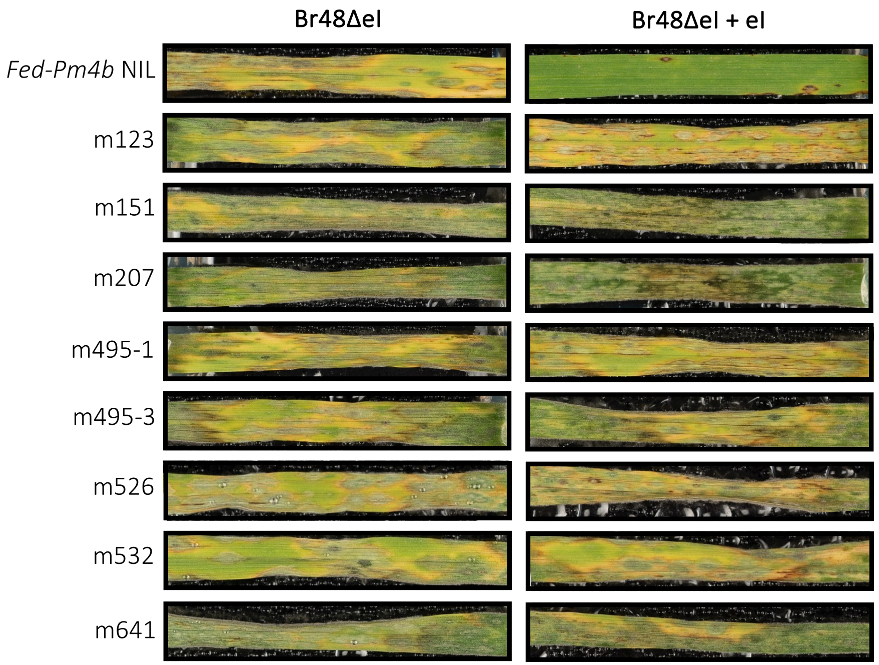

Figure S10 - Wheat blast detached leaf assays for *Pm4b* EMS-induced mutants of *Fed-Pm4b* NIL. Leaves were inoculated with Br48ΔeI and Br48ΔeI+eI.

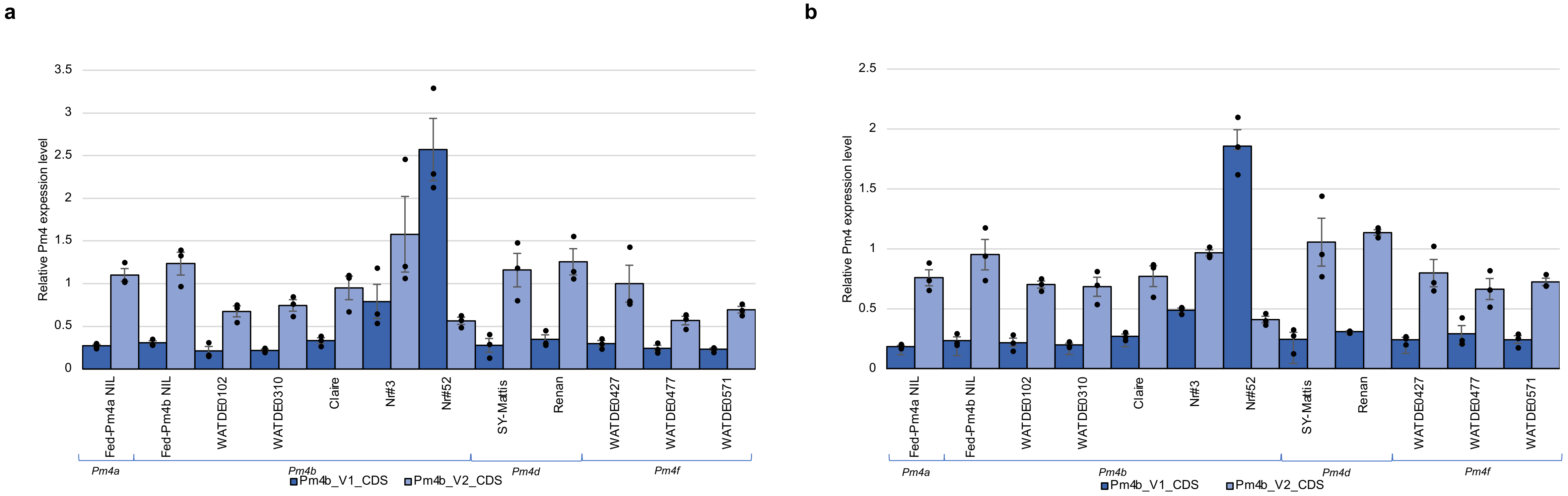

Figure S11 – Expression levels of Pm4b_V1_CDS (dark blue) and Pm4b_V2_CDS (light blue) in the spike relative to reference genes (a) ADP and (b) ZFL. The bars comprise the mean of three biological replicates, each with two technical replicates. Datapoints for the biological replicates are represented by black dots. 'Nr#' = Pm4b transgenics (over expressors). The *Pm4* allele present in each accession is shown underneath the brackets. Error bars show the standard error. Statistical analysis was performed using Genstat version 22.1 (VSN International 2022).

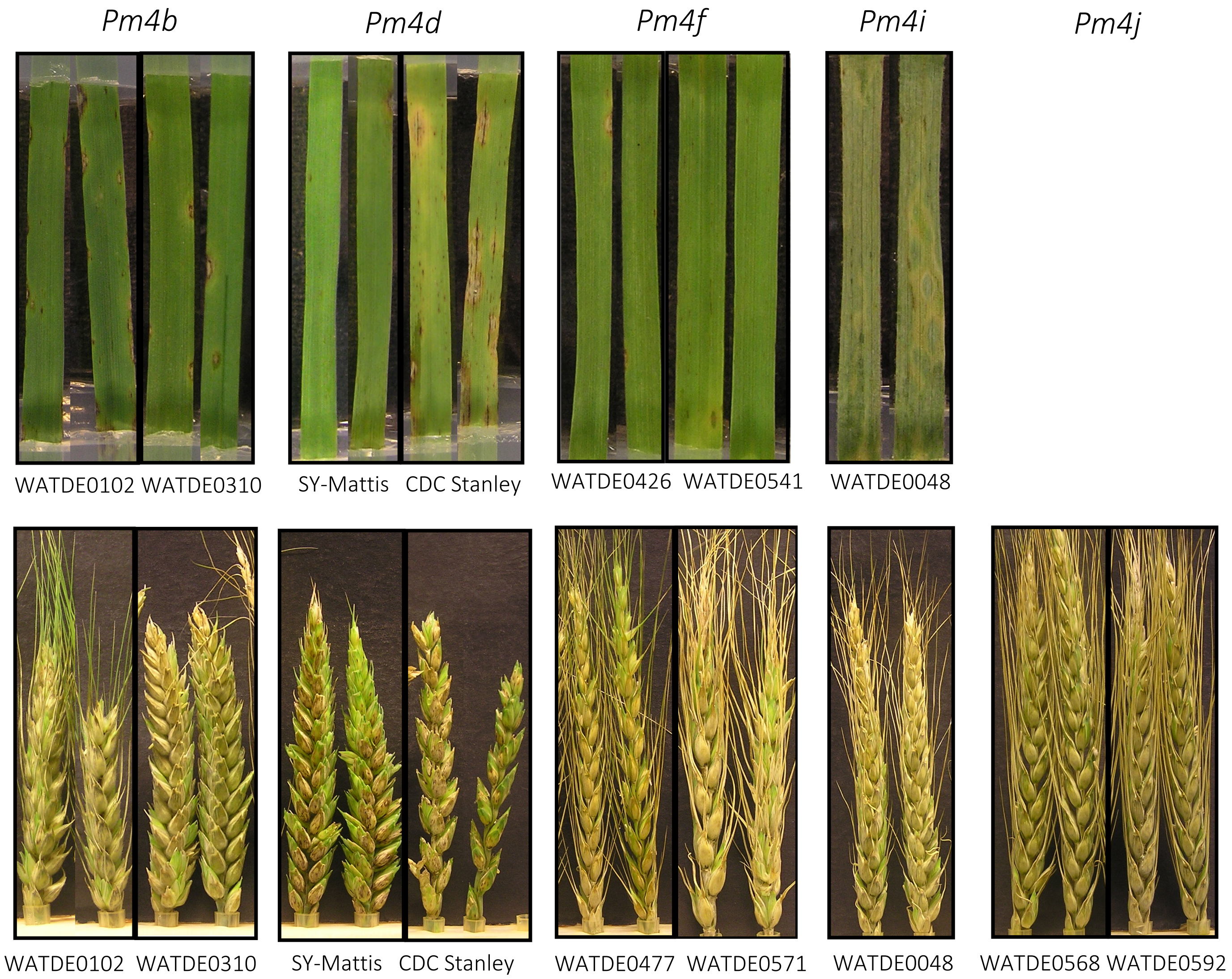

Figure S12 - Wheat blast detached leaf and spike assays for *Pm4* alleles inoculated with *Py 15.1.018* at 26 °C. Images were taken at five and four days post inoculation for leaves and spikes, respectively.

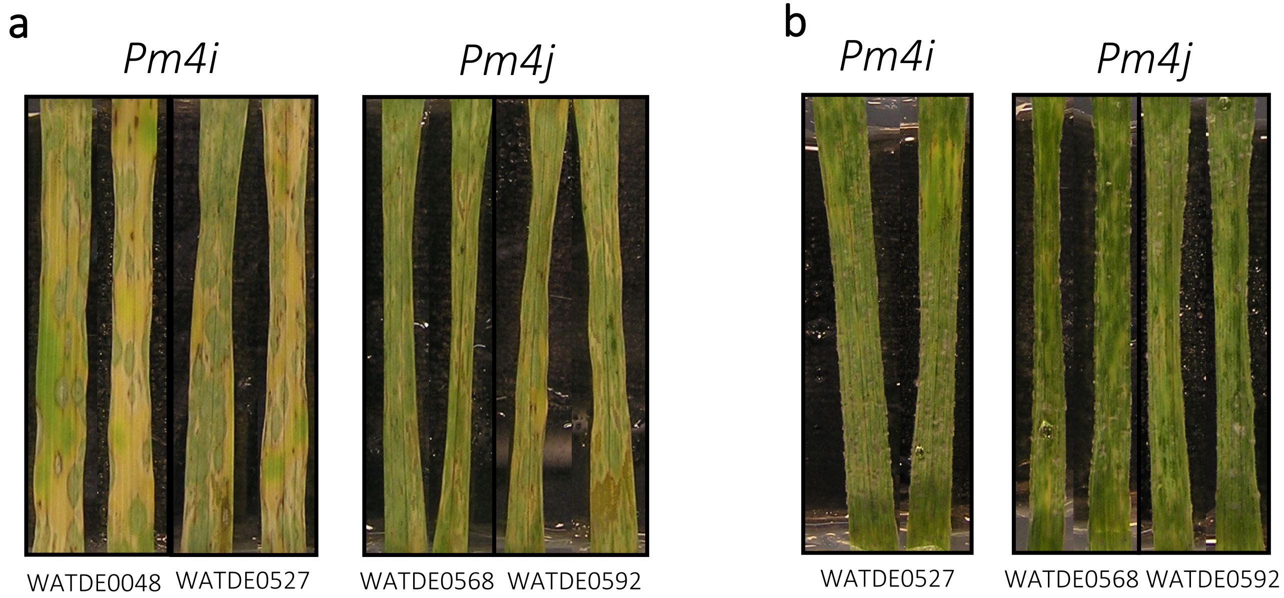

Figure S13 - Wheat blast detached leaf and spike assays for *Pm4i* and *Pm4j* alleles inoculated with a. NO6047+*AVR8* **b**. Py 15.1.018 at 22°C. Images were taken at five and six days post inoculation for NO6047+*AVR8* **and** Py 15.1.018 respectively.

Table S1 - List of blast resistance loci identified and mapped in wheat. Note only *Rwt3* and *Rwt4* have been cloned (Arora et al. 2023).

Gene	Source cultivar	Effective against	*Avr* genes	Chromosome	References
Rmg1 (Rwt4)	Norin 4	*Avena* isolate Br58	*PWT4*	1D (cloned)	(Takabayashi et al. 2002; Arora et al. 2023)
Rmg2	Thatcher	*Triticum* isolate Br48	Not known	7A	(Zhan et al. 2008)
Rmg3	Thatcher	*Triticum* isolate Br48	Not known	6D	(Zhan et al. 2008)
Rmg4	P168, Shin-chunaga, Norin 4, Norin 26, Norin 29	*Digitaria* isolate	Not known	4A	(Nga et al. 2009)
Rmg5	Red Egyptian and Salmon	*Digitaria* isolate	Not known	6D	(Nga et al. 2009)
Rmg6 (Rwt3)	Chinese Spring, Shin-chunaga and Norin 4	*Lolium* isolate TP2	*PWT3* (or *A1*)	1D (cloned)	(Vy et al. 2014; Arora et al. 2023)
Rmg7	St24, St17, St25	*Triticum* isolate Br48	*AVR-Rmg7*	2A	(Tagle et al. 2015)
Rmg8	S-615	*Triticum* isolate Br48	*AVR-Rmg8* (=*AVR-Rmg7*)	2B	(Anh et al. 2015; Anh et al. 2018)
RmgGR119	GR119	*Triticum* isolate Br48	Not known	-	(Wang et al. 2018)
2NS	VPM1	*Triticum* isolate Br48	Not known	2A	(Doussinault et al 1981)

Table S2 - The wheat cultivars with chromosome or scaffold-scale assemblies used in this study, their pedigree, origins and assembly type. Adapted from Supplementary Table 1 of Walkowiak et al. (2020). RQA: Reference Quality Pseudomolecule Assembly

Line	Pedigree	Growth Habit	Origin	Assembly Type
Mace	WYALKATCHEM/STYLET//WYALKATCHEM	Spring	Australia	RQA
LongReach Lancer	VI184/Chara//Chara/3/Lang	Spring	Australia	RQA
CDC Stanley	CDC Teal//EE8/Kenyon35//AC Barrie	Spring	Canada	RQA
CDC Landmark	Unity/Waskada//Alsen/Superb	Spring	Canada	RQA
Julius	Asketis/Drifter	Winter	Germany	RQA
Norin 61	Fukuoka Komugi 18/Shinchunaga	Facultative Spring	Japan	RQA
Arina*LrFor*	Arina*3/Forno	Winter	Switzerland	RQA
Jagger	KS-82-W-418/STEPHENS	Winter	USA	RQA
SY-Mattis	Apache/Intense	Winter	France	RQA
Cadenza	AXONA/TONIC	Spring	UK	Scaffold
Paragon	CSW-1724-19-5-69//Axona/Tonic	Spring	UK	Scaffold
Robigus	1366/Z-836	Winter	UK	Scaffold
Claire	WASP/FLAME	Winter	UK	Scaffold

Table S3 - *MoT* isolates used in this study.

Isolate code	Origin	Date collected	Source
BR32	Brazil	1991	Lesley Boyd, JIC
BR48	Brazil	1990	Lesley Boyd, JIC
NO6047	Brazil	2006	Diane Saunders, JIC
NO6047+*AVR8*	-	-	Diane Saunders, JIC
Py 15.1.018	Brazil	2015	Embrapa
BTJ4P-1	Bangladesh	2016	Tofazzal Islam, BSMRU, Bangladesh
BR48ΔeI	-	-	Yukio Tosa, Kobe University
BR48ΔeI+eI	-	-	Yukio Tosa, Kobe University
BR48ΔeI+eII	-	-	Yukio Tosa, Kobe University
BR48ΔeI+eII'	-	-	Yukio Tosa, Kobe University

Table S4 - Detached leaf phenotype data for the Watkins core panel screened with NO6047+*AVR8* and Py 15.1.018. JIC GRU Store code refers to the accession identifier in the John Innes Centre Germplasm Resources Unit (www.seedstor.ac.uk). Accessions with only 1 replicate were excluded from the 'Mean' columns.

JIC GRU store code	Detached leaf assay phenotype (Mean score at 5 dpi)								Contains 2A association peak
	NO6047+*AVR8*				Py 15.1.018				
	Rep 1	Rep 2	Rep 3	Mean	Rep 1	Rep 2	Rep 3	Mean	
WATDE0477	0	0	0	0.00	0	0	0	0.00	2A
WATDE0973	0	0	1	0.33	0	0	1	0.33	2A
WATDE0171	1	0	0	0.33	2	1	1	1.33	2A
WATDE0058	0	0	1	0.33	4	3	6	4.33	
WATDE0310	1	1	0	0.67	0	0	0	0.00	2A
WATDE0571	0	1	1	0.67	0	0	0	0.00	2A
WATDE0971	0	1	1	0.67	0	0	0	0.00	2A
WATDE0427	1	1	1	1.00	0	0	-	0.00	2A
WATDE0526	1	1	1	1.00	1	1	0	0.67	2A
WATDE0102	1	1	1	1.00	1	1	1	1.00	2A
WATDE0126	1	1	1	1.00	3	-	2	2.50	
WATDE0046	2	1	1	1.33	3	1	3	2.33	
WATDE0381	1	1	2	1.33	2	4	2	2.67	
WATDE0779	2	1	2	1.67	2	2	2	2.00	
WATDE0051	1	2	2	1.67	1	4	3	2.67	
WATDE0749	2	2	1	1.67	3	4	4	3.67	
WATDE0601	2	2	1	1.67	4	4	4	4.00	
WATDE0066	2	2	1	1.67	4	5	5	4.67	
WATDE0216	3	2	1	2.00	3	3	4	3.33	
WATDE0519	1	3	2	2.00	4	1	5	3.33	
WATDE0405	3	2	1	2.00	4	4	4	4.00	

WATDE0747	3	1	2	2.00	4	4	4	4.00	
WATDE0984	3	1	2	2.00	5	4	5	4.67	
WATDE0015	2	3	2	2.33	0	-	1	0.50	
WATDE0090	2	1	4	2.33	1	2	3	2.00	
WATDE0795	4	1	2	2.33	2	3	2	2.33	
WATDE0386	2	3	2	2.33	4	2	2	2.67	
WATDE0019	3	3	1	2.33	4	3	4	3.67	
WATDE0241	3	1	3	2.33	3	4	6	4.33	
WATDE0702	2	2	3	2.33	4	4	5	4.33	
WATDE0679	3	2	2	2.33	4	6	5	5.00	
WATDE0266	3	2	2	2.33	5	6	6	5.67	
WATDE0268	3	3	2	2.67	3	2	2	2.33	
WATDE0771	2	4	2	2.67	3	3	2	2.67	
WATDE0811	3	1	4	2.67	3	3	3	3.00	
WATDE0004	2	4	2	2.67	3	3	4	3.33	
WATDE0075	2	3	3	2.67	3	4	4	3.67	
WATDE0198	3	3	2	2.67	4	3	4	3.67	
WATDE0532	2	4	2	2.67	3	3	5	3.67	
WATDE0238	3	2	3	2.67	4	4	5	4.33	
WATDE0669	3	3	2	2.67	3	4	6	4.33	
WATDE0596	2	3	3	2.67	5	6	6	5.67	
WATDE0369	4	1	4	3.00	0	0	4	1.33	2A
WATDE0026	3	3	3	3.00	1	1	3	1.67	
WATDE0029	3	2	4	3.00	2	5	3	3.33	
WATDE0827	4	2	3	3.00	3	3	4	3.33	
WATDE0929	3	2	4	3.00	4	4	3	3.67	
WATDE0034	3	3	3	3.00	4	4	4	4.00	
WATDE0192	4	2	3	3.00	5	3	4	4.00	
WATDE0703	2	4	3	3.00	3	5	-	4.00	
WATDE0067	3	3	3	3.00	3	5	5	4.33	

WATDE0222	4	-	2	3.00	6	5	3	4.67
WATDE0305	3	3	3	3.00	5	4	5	4.67
WATDE0668	4	3	2	3.00	4	6	5	5.00
WATDE0751	4	3	2	3.00	5	5	5	5.00
WATDE0613	2	4	3	3.00	4	6	6	5.33
WATDE0758	3	2	4	3.00	6	5	6	5.67
WATDE0765	4	3	3	3.33	2	3	4	3.00
WATDE0003	3	3	4	3.33	4	3	4	3.67
WATDE0579	4	3	3	3.33	4	4	3	3.67
WATDE0898	3	4	3	3.33	3	5	3	3.67
WATDE0008	4	3	3	3.33	5	4	3	4.00
WATDE0581	3	4	3	3.33	4	4	4	4.00
WATDE0604	3	4	3	3.33	2	-	6	4.00
WATDE0819	4	2	4	3.33	6	3	3	4.00
WATDE0010	4	2	4	3.33	5	5	3	4.33
WATDE0049	3	3	4	3.33	4	5	4	4.33
WATDE0732	3	3	4	3.33	6	3	5	4.67
WATDE0457	3	4	3	3.33	5	5	5	5.00
WATDE0708	3	4	3	3.33	4	6	5	5.00
WATDE0011	4	3	3	3.33	5	6	5	5.33
WATDE0104	4	3	3	3.33	4	6	6	5.33
WATDE0611	3	4	3	3.33	6	6	5	5.67
WATDE0077	4	3	4	3.67	2	3	3	2.67
WATDE0050	4	4	3	3.67	3	2	5	3.33
WATDE0073	3	4	4	3.67	2	5	4	3.67
WATDE0770	3	4	4	3.67	4	3	5	4.00
WATDE0027	2	4	5	3.67	4	3	6	4.33
WATDE0542	3	4	4	3.67	4	4	5	4.33
WATDE0705	3	4	4	3.67	3	5	5	4.33
WATDE0001	4	3	4	3.67	5	-	4	4.50

WATDE0083	3	4	4	3.67	5	5	4	4.67
WATDE0005	4	4	3	3.67	5	5	5	5.00
WATDE0052	3	4	4	3.67	5	6	4	5.00
WATDE0072	2	4	5	3.67	4	5	6	5.00
WATDE0582	3	4	4	3.67	5	5	5	5.00
WATDE0042	3	4	4	3.67	5	6	5	5.33
WATDE0099	5	3	3	3.67	-	6	5	5.50
CIMCOG 47	4	4	3	3.67	6	6	5	5.67
WATDE0038	4	3	4	3.67	5	6	6	5.67
WATDE0133	3	2	6	3.67	6	-	6	6.00
WATDE0550	3	4	4	3.67	6	6	6	6.00
WATDE0651	3	3	5	3.67	6	6	6	6.00
WATDE0670	4	3	4	3.67	6	6	6	6.00
WATDE0086	4	4	4	4.00	2	-	4	3.00
WATDE0032	3	4	5	4.00	3	3	4	3.33
WATDE0070	4	4	4	4.00	3	4	3	3.33
WATDE0450	5	3	4	4.00	3	4	3	3.33
WATDE0762	3	4	5	4.00	3	4	3	3.33
WATDE0033	4	5	3	4.00	3	4	4	3.67
WATDE0074	4	4	4	4.00	6	3	2	3.67
WATDE0776	4	3	5	4.00	3	3	5	3.67
WATDE0025	5	3	4	4.00	2	5	5	4.00
WATDE0076	4	4	4	4.00	4	5	3	4.00
WATDE0725	3	5	4	4.00	6	3	3	4.00
WATDE0865	4	4	4	4.00	3	5	4	4.00
WATDE0392	4	4	4	4.00	3	6	4	4.33
WATDE0694	4	4	4	4.00	3	5	5	4.33
WATDE0336	5	3	4	4.00	6	4	4	4.67
WATDE0476	3	4	5	4.00	4	5	5	4.67
WATDE0843	4	4	4	4.00	6	3	5	4.67

WATDE0037	5	3	4	4.00	3	6	6	5.00
WATDE0737	4	4	4	4.00	5	6	4	5.00
WATDE0007	4	4	4	4.00	5	6	5	5.33
WATDE0040	5	3	4	4.00	6	5	5	5.33
WATDE0594	3	5	4	4.00	4	6	6	5.33
WATDE0509	3	4	5	4.00	5	6	6	5.67
WATDE0678	4	4	4	4.00	5	6	6	5.67
WATDE0107	3	4	5	4.00	6	6	6	6.00
WATDE0149	5	3	4	4.00	6	6	6	6.00
WATDE0063	4	3	5	4.00	6	-	-	-
WATDE0798	5	4	4	4.33	4	1	4	3.00
WATDE0773	4	5	4	4.33	4	3	3	3.33
WATDE0002	5	4	4	4.33	4	-	3	3.50
WATDE0009	4	5	4	4.33	4	3	4	3.67
WATDE0385	4	4	5	4.33	4	3	4	3.67
WATDE0156	5	5	3	4.33	3	4	5	4.00
WATDE0196	5	4	4	4.33	3	5	-	4.00
CIMCOG 32	3	5	5	4.33	3	6	4	4.33
WATDE0021	5	4	4	4.33	4	4	5	4.33
WATDE0518	4	5	4	4.33	5	4	4	4.33
WATDE0634	4	4	5	4.33	4	4	5	4.33
WATDE0892	4	5	4	4.33	3	4	6	4.33
WATDE0081	4	4	5	4.33	4	5	5	4.67
WATDE0180	4	4	5	4.33	4	5	5	4.67
WATDE0743	4	5	4	4.33	4	6	4	4.67
WATDE0020	4	5	4	4.33	5	5	5	5.00
WATDE0047	4	4	5	4.33	6	4	5	5.00
WATDE0096	4	4	5	4.33	5	5	5	5.00
WATDE0740	4	4	5	4.33	6	5	4	5.00
WATDE0991	4	4	5	4.33	5	4	6	5.00

WATDE0048	4	5	4	4.33	6	4	6	5.33
WATDE0664	4	4	5	4.33	6	5	5	5.33
WATDE1051	5	5	3	4.33	6	4	6	5.33
WATDE0022	5	4	4	4.33	6	5	6	5.67
WATDE0055	6	4	3	4.33	5	6	6	5.67
WATDE0335	5	4	4	4.33	6	5	6	5.67
WATDE0430	4	4	5	4.33	6	6	5	5.67
WATDE0035	4	4	5	4.33	6	6	6	6.00
WATDE0039	5	4	4	4.33	6	6	6	6.00
WATDE0101	4	4	5	4.33	6	6	6	6.00
WATDE0263	4	5	4	4.33	6	6	6	6.00
WATDE0521	4	4	5	4.33	6	6	6	6.00
WATDE0592	5	4	4	4.33	6	6	6	6.00
WATDE0882	5	4	4	4.33	6	6	-	6.00
WATDE0989	4	4	5	4.33	6	6	6	6.00
WATDE1012	3	5	5	4.33	6	6	6	6.00
WATDE0023	4	5	-	4.50	6	6	6	6.00
WATDE0071	3	6	5	4.67	2	-	-	2.00
WATDE0868	5	4	5	4.67	2	4	2	2.67
WATDE0761	4	5	5	4.67	2	4	3	3.00
WATDE0018	5	5	4	4.67	4	3	3	3.33
WATDE0816	4	4	6	4.67	3	4	-	3.50
WATDE0250	5	5	4	4.67	2	4	5	3.67
WATDE0808	4	5	5	4.67	4	3	4	3.67
WATDE0082	4	4	6	4.67	4	4	-	4.00
WATDE0089	5	4	5	4.67	6	3	3	4.00
WATDE0555	4	6	4	4.67	3	5	4	4.00
WATDE0068	4	5	5	4.67	3	4	6	4.33
WATDE0078	5	4	5	4.67	5	5	3	4.33
WATDE0294	5	5	4	4.67	4	5	4	4.33

WATDE0435	5	4	5	4.67	4	4	5	4.33
WATDE0558	5	4	5	4.67	4	5	4	4.33
CIMCOG 26	6	4	4	4.67	5	5	4	4.67
WATDE0030	5	5	4	4.67	4	5	5	4.67
WATDE0057	4	5	5	4.67	3	6	5	4.67
WATDE0445	5	5	4	4.67	2	6	6	4.67
WATDE0576	5	5	4	4.67	3	6	5	4.67
WATDE0831	5	4	5	4.67	4	4	6	4.67
WATDE0950	5	5	4	4.67	4	5	5	4.67
WATDE0013	6	3	5	4.67	6	4	5	5.00
WATDE0963	6	2	6	4.67	4	5	6	5.00
WATDE0024	5	4	5	4.67	5	5	6	5.33
WATDE0041	5	5	4	4.67	5	5	6	5.33
WATDE0249	6	4	4	4.67	6	5	5	5.33
WATDE0359	5	4	5	4.67	5	5	6	5.33
WATDE0588	4	4	6	4.67	5	5	6	5.33
WATDE0954	4	4	6	4.67	4	6	6	5.33
WATDE0456	5	4	5	4.67	5	6	-	5.50
WATDE0451	5	4	5	4.67	5	6	6	5.67
WATDE0646	4	5	5	4.67	6	5	6	5.67
WATDE0833	4	5	5	4.67	6	5	6	5.67
CIMCOG 03	6	4	4	4.67	6	6	6	6.00
CIMCOG 56	5	4	5	4.67	6	6	6	6.00
WATDE0092	5	4	5	4.67	6	6	6	6.00
WATDE0095	5	4	5	4.67	6	6	6	6.00
WATDE0228	5	5	4	4.67	6	6	6	6.00
WATDE0422	5	4	5	4.67	6	6	6	6.00
WATDE0459	4	5	5	4.67	6	6	6	6.00
WATDE1027	4	5	5	4.67	6	6	6	6.00
WATDE0290	5	5	-	5.00	4	3	3	3.33

WATDE0788	6	4	5	5.00	2	4	4	3.33
WATDE0253	5	4	6	5.00	2	4	5	3.67
WATDE0339	5	4	6	5.00	5	5	4	4.67
WATDE0919	6	4	5	5.00	5	5	4	4.67
WATDE0801	6	5	4	5.00	4	6	5	5.00
WATDE0863	5	5	5	5.00	5	4	6	5.00
Sup152 CIMCOG39	6	5	4	5.00	6	4	6	5.33
WATDE0031	4	5	6	5.00	6	5	5	5.33
WATDE0100	6	5	4	5.00	6	6	4	5.33
WATDE0714	4	6	5	5.00	4	6	6	5.33
WATDE0871	5	4	6	5.00	4	6	6	5.33
Weebil	5	5	5	5.00	6	4	6	5.33
WATDE0054	5	5	5	5.00	5	6	6	5.67
WATDE0612	5	4	6	5.00	6	6	5	5.67
WATDE0643	4	5	6	5.00	6	5	6	5.67
CIMCOG 12	6	5	4	5.00	6	6	6	6.00
Fielder	5	6	4	5.00	6	6	6	6.00
WATDE0113	6	4	5	5.00	6	6	6	6.00
WATDE0138	5	5	5	5.00	6	-	6	6.00
WATDE0215	5	4	6	5.00	6	6	6	6.00
WATDE0292	5	5	5	5.00	6	6	6	6.00
WATDE0639	5	5	5	5.00	6	6	6	6.00
WATDE0909	5	5	5	5.00	6	6	6	6.00
WATDE0993	5	5	5	5.00	6	6	6	6.00
Reedling	5	5	5	5.00	6	6	6	-
WATDE0727	4	5	6	5.00	6	-	-	-
WATDE0873	5	5	6	5.33	1	2	2	1.67
WATDE0069	6	5	5	5.33	3	-	-	3.00
WATDE0782	5	5	6	5.33	5	4	4	4.33
WATDE0934	5	6	5	5.33	4	5	4	4.33

WATDE0864	5	5	6	5.33	5	4	-	4.50
WATDE0056	6	5	5	5.33	2	6	6	4.67
WATDE0098	6	5	5	5.33	5	4	5	4.67
WATDE0108	5	6	5	5.33	4	4	6	4.67
WATDE0574	6	5	5	5.33	5	5	5	5.00
WATDE0861	5	6	5	5.33	4	5	6	5.00
WATDE0939	5	5	6	5.33	5	5	5	5.00
WATDE1052	6	5	5	5.33	6	4	5	5.00
WATDE0060	6	5	5	5.33	4	6	6	5.33
WATDE0112	6	5	5	5.33	6	5	5	5.33
WATDE0088	6	4	6	5.33	6	5	-	5.50
WATDE0016	4	6	6	5.33	5	6	6	5.67
WATDE0585	6	5	5	5.33	5	6	6	5.67
WATDE0857	6	6	4	5.33	6	5	6	5.67
Baj	6	5	5	5.33	6	6	6	6.00
Chinese Spring	6	5	5	5.33	6	6	6	6.00
Paragon	5	6	5	5.33	6	6	6	6.00
WATDE0091	5	6	5	5.33	6	6	6	6.00
WATDE0110	4	6	6	5.33	6	6	6	6.00
WATDE0116	5	6	5	5.33	6	6	6	6.00
WATDE0420	6	5	5	5.33	6	6	-	6.00
WATDE0534	5	5	6	5.33	-	6	6	6.00
WATDE0557	5	5	6	5.33	6	6	6	6.00
WATDE0609	4	6	6	5.33	6	6	6	6.00
WATDE0631	6	6	4	5.33	6	6	6	6.00
WATDE0661	6	4	6	5.33	-	-	6	6.00
WATDE0671	6	4	6	5.33	6	6	6	6.00
Waxwing	5	5	6	5.33	6	6	6	6.00
WATDE0064	6	5	5	5.33	6	6	6	-
WATDE0017	6	6	5	5.67	4	4	4	4.00

WATDE0028	6	6	5	5.67	3	6	4	4.33
WATDE0382	6	6	5	5.67	4	4	6	4.67
WATDE0461	6	5	6	5.67	2	6	6	4.67
WATDE0053	6	5	6	5.67	4	6	5	5.00
WATDE0308	6	6	5	5.67	5	4	6	5.00
WATDE0699	5	6	6	5.67	4	5	6	5.00
WATDE0061	6	5	6	5.67	6	4	6	5.33
WATDE0094	6	5	6	5.67	6	6	5	5.67
WATDE0106	5	6	6	5.67	5	6	6	5.67
WATDE0455	6	6	5	5.67	5	6	6	5.67
WATDE0479	5	6	6	5.67	6	6	5	5.67
WATDE0986	6	5	6	5.67	5	6	6	5.67
CIMCOG 49	6	5	6	5.67	6	-	6	6.00
CIMCOG 53	6	5	6	5.67	6	6	6	6.00
MISR1 CIMCOG33	6	6	5	5.67	6	6	6	6.00
WATDE0093	6	6	5	5.67	6	6	6	6.00
WATDE0097	5	6	6	5.67	6	6	6	6.00
WATDE0111	6	6	5	5.67	6	6	6	6.00
WATDE0394	6	5	6	5.67	6	6	6	6.00
WATDE0577	6	6	5	5.67	6	6	6	6.00
WATDE0617	5	6	6	5.67	6	6	6	6.00
WATDE0659	6	5	6	5.67	6	-	6	6.00
WATDE0883	5	6	6	5.67	6	6	6	6.00
WATDE1025	5	6	6	5.67	6	6	6	6.00
WATDE1030	6	5	6	5.67	6	6	6	6.00
WATDE0276	6	6	6	6.00	5	4	2	3.67
WATDE0278	6	6	6	6.00	4	4	4	4.00
WATDE0375	6	6	6	6.00	4	6	3	4.33
WATDE0774	6	6	6	6.00	5	5	3	4.33
WATDE0712	6	6	6	6.00	3	6	5	4.67

WATDE0791	6	6	6	6.00	5	5	5	5.00
WATDE0932	6	6	6	6.00	5	6	4	5.00
WATDE0937	6	6	6	6.00	5	6	4	5.00
WATDE0036	6	6	6	6.00	6	4	6	5.33
WATDE0262	6	6	6	6.00	4	6	6	5.33
WATDE0911	6	6	6	6.00	5	6	-	5.50
WATDE0105	6	6	6	6.00	6	6	5	5.67
WATDE0635	6	6	6	6.00	5	6	6	5.67
WATDE0835	6	6	6	6.00	6	6	5	5.67
WATDE0938	6	6	6	6.00	6	5	6	5.67
Pamyat Azieva	6	6	6	6.00	6	6	6	6.00
WATDE0080	6	6	6	6.00	6	6	6	6.00
WATDE0087	6	6	6	6.00	6	6	6	6.00
WATDE0114	6	6	6	6.00	6	6	6	6.00
WATDE0115	6	6	6	6.00	6	6	6	6.00
WATDE0117	6	6	6	6.00	6	6	6	6.00
WATDE0118	6	6	6	6.00	6	6	6	6.00
WATDE0396	6	6	6	6.00	6	6	6	6.00
WATDE0397	6	6	6	6.00	6	6	6	6.00
WATDE0486	6	6	6	6.00	6	6	6	6.00
WATDE0653	6	6	6	6.00	6	6	6	6.00
WATDE0888	6	6	6	6.00	6	6	6	6.00
WATDE0895	6	6	6	6.00	6	6	6	6.00
WATDE1026	6	6	6	6.00	6	6	6	6.00
WATDE1060	6	6	6	6.00	6	6	6	6.00
Pfau	6	6	6	6.00	-	-	6	-
Sears Synth Type	2	-	-	-	1	2	1	1.33
WATDE0062	5	4	5	-	3	1	2	2.00
Wyalkatchem	-	-	-	-	5	-	5	5.00

Table S5 - Additional Watkins cultivars used to verify the 2A resistance, their origins, growth habit and detached leaf (DLA) and spike (DSA) assay phenotype at five days post inoculation with isolate Py 15.1.018. Phenotype was predicted based on the presense of 'Region 1' or 'Region 2' following haplotype analysis.

GRU Store Code	Collection ID	Origin Country	Growth Habit	Predicted phenotype	Mean DLA score (5 dpi)	Mean DSA score (5 dpi)
WATDE0056	Wat1190433-1	India	Spring	Resistant	5.20	4.90
WATDE0426	Wat1190267-1	Spain	Spring	Resistant	1.00	0.70
WATDE0428	Wat1190269-1	Spain	Spring	Resistant	1.00	2.70
WATDE0465	Wat1190309-1	Iran	Winter	Resistant	1.20	-
WATDE0484	Wat1190320-3	China	Winter	Resistant	1.60	3.00
WATDE0505	Wat1190337-1	Hungary	Spring	Resistant	1.20	5.00
WATDE0527	Wat1190357-2	Yugoslavia	Spring	Resistant	5.00	5.70
WATDE0541	Wat1190369-2	Yugoslavia	Spring	Resistant	0.80	2.70
WATDE0546	Wat1190373-1	Iran	Spring	Resistant	0.60	2.30
WATDE0566	Wat1190389-1	Portugal	Spring	Resistant	1.20	2.00
WATDE0567	Wat1190390-1	Portugal	Spring	Resistant	1.20	4.00
WATDE0568	Wat1190391-1	Portugal	Spring	Resistant	5.40	6.00
WATDE0592	Wat1190412-1	India	Spring	Resistant	6.00	6.00
WATDE0672	Wat1190488-1	USSR	Winter	Resistant	1.20	2.50
WATDE0687	Wat1190500-1	Iraq	Winter	Resistant	0.80	3.00
WATDE0720	Wat1190525-1	India	Spring	Resistant	5.40	6.00
WATDE0122	Wat1190003-4	Iran	Spring	Susceptible	5.60	5.30
WATDE0141	Wat1190017-2	Spain	Winter	Susceptible	5.40	6.00
WATDE0152	Wat1190027-1	Australia	Spring	Susceptible	5.60	-
WATDE0162	Wat1190037-1	Poland	Winter	Susceptible	6.00	6.00

WATDE0175	Wat1190049-1	Spain	Spring	Susceptible	6.00	6.00
WATDE0185	Wat1190058-1	Portugal	Spring	Susceptible	5.60	-
WATDE0199	Wat1190068-1	Spain	Spring	Susceptible	5.80	-
WATDE0207	Wat1190075-1	Yugoslavia	Winter	Susceptible	6.00	5.70
WATDE0220	Wat1190086-2	India	Spring	Susceptible	4.60	-
WATDE0232	Wat1190097-1	Poland	Winter	Susceptible	6.00	4.80
WATDE0804	Wat1190601-1	Spain	Spring	Resistant	1.60	4.00
WATDE0970	Wat1190758-2	Italy	Spring	Resistant	1.00	2.70
WATDE0974	Wat1190761-1	USSR	Spring	Resistant	1.00	3.70
WATDE1062	Wat1190911-1	Hungary	Spring	Resistant	1.20	2.70

Table S6 - Adapted wheat varieties used in this study, along with their detached leaf assay phenotype using NO6047+*AVR8* and Py 15.1.018 isolates. Scores were taken at six days post inoculation.

Accession	JIC GRU store code	Detached leaf assay with NO6047+*AVR8*	Detached leaf assay with Py 15.1.018
Ability	WGED0562	2.33	1.00
Boxer	WGED0540	1.67	1.00
Claire	PANG0005	1.20	1.20
Consort	WGED0670	3.00	3.00
Cordiale	W10003	-	-
Crest	WGED0666	3.00	2.00
Encore	WGED0671	2.33	2.00
Extase	-	-	-
Flame	WGED0155	2.33	1.00
Grafton	-	-	-
Holster	WGED0663	3.00	3.00
Kosack	-	-	-
Legend	WGED0561	1.33	1.33
Malacca	WGED0639	3.33	2.00
SY-Mattis	PANG0015	1.80	1.20
Reform	-	-	-
Renan	WGED0248	2.00	0.80
Rendevous	-	2.60	1.80
Revelation	W10190	1.40	1.20
Riband	WGED0128	3.00	1.67
Savannah	WGED0280	2.67	1.67
Shango	WGED0713	2.33	1.67
Spark	WGED0159	0.67	1.33
Spitfire	WGED0712	2.00	1.67

CDC Stanley	PANG0004	2.60	1.20
Stava	WGED0211	1.67	1.00
SY-Epson	W10079	1.20	0.00
Torfrida	WGED0161	3.00	1.33
Turpin	WGED0629	3.67	1.67
Wasp	WGED0630	2.00	1.00

Table S7 - High confidence gene content of the SY-Mattis 2A interval, their location and function from the *de novo* genome annotation. Gene content of the 400kb SY-Mattis haplotype (78855000 to 788950000 kb) is highlighted in yellow.

Gene code	Coordinates (orientation)	Function
TraesSYM2A03G00828360	788728552 - 788738447(+)	Receptor-like kinase
TraesSYM2A03G00828370	788768076 - 788769438(+)	*unknown*
TraesSYM2A03G00828380	788811120 - 788813175(+)	*unknown*
TraesSYM2A03G00828390	788825472 - 788828016(+)	*unknown*
TraesSYM2A03G00828400	788828264 - 788832085(+)	methyltransferases superfamily protein
TraesSYM2A03G00828410	788833791 - 788837343(+)	RNase P 1
TraesSYM2A03G00828420	788837936 - 788839387(+)	STAY-GREEN LIKE, chloroplastic
TraesSYM2A03G00828440	788841216 - 788842285(+)	*unknown*
TraesSYM2A03G00828450	788845950 - 788849102(+)	resistance protein RGA2
TraesSYM2A03G00828460	788885045 - 788890732(+)	resistance protein RGA2
TraesSYM2A03G00828500	788960342 - 788962593(+)	*unknown*
TraesSYM2A03G00828540	789110507 - 789111037(+)	*unknown*
TraesSYM2A03G00828580	789151116 - 789153155(+)	*unknown*
TraesSYM2A03G00828590	789161156 - 789162436(+)	F-box/kelch-repeat protein
TraesSYM2A03G00828600	789188895 - 789193763(+)	resistance protein RGA2/ transposon TNT
TraesSYM2A03G00828640	789217066 - 789233090(+)	F-box/kelch-repeat protein
TraesSYM2A03G00828700	789264657 - 789265305(-)	protein 5NG4
TraesSYM2A03G00828720	789284536 - 789289459(+)	resistance protein RGA2
TraesSYM2A03G00828810	789390224 - 789396435(+)	resistance protein RGA2

TraesSYM2A03G00828830	789403251 - 789408104(+)	resistance protein RGA2
TraesSYM2A03G00828840	789408643 - 789409011(-)	peptidase subunit alpha
TraesSYM2A03G00828850	789416992 - 789419026(+)	phosphodiesterases superfamily protein
TraesSYM2A03G00828930	789745589 - 789748504(-)	4-like protein
TraesSYM2A03G00829020	790122656 - 790126161(-)	*unknown*
TraesSYM2A03G00829080	790381831 - 790387386(+)	*unknown*
TraesSYM2A03G00829120	790568231 - 790571224(-)	*unknown*
TraesSYM2A03G00829200	790955915 - 790959358(-)	*unknown*
TraesSYM2A03G00829250	791221535 - 791225005(+)	*unknown*
TraesSYM2A03G00829350	791855004 - 791858879(-)	*unknown*
TraesSYM2A03G00829450	792130947 - 792134542(+)	*unknown*
TraesSYM2A03G00829510	792400216 - 792404527(-)	N-methyltransferase SUVR4
TraesSYM2A03G00829520	792405026 - 792406692(+)	non-LTR retrotransposon
TraesSYM2A03G00829540	792410750 - 792414847(-)	glycosyltransferase subunit 1
TraesSYM2A03G00829560	792465474 - 792467366(-)	beta-1,6-N-acetylglucosaminyltransferase family protein
TraesSYM2A03G00829570	792472433 - 792473359(+)	*unknown*
TraesSYM2A03G00829580	792519361 - 792525805(-)	toxin-like protein Hfr-2
TraesSYM2A03G00829600	792527494 - 792528414(-)	*unknown*
TraesSYM2A03G00829610	792534358 - 792535178(+)	*unknown*
TraesSYM2A03G00829620	792554319 - 792557912(+)	domain-containing protein 78
TraesSYM2A03G00829630	792559869 - 792562916(+)	protein Rab-18
TraesSYM2A03G00829640	792660864 - 792662136(+)	*unknown*
TraesSYM2A03G00829650	792660869 - 792662507(-)	*unknown*
TraesSYM2A03G00829660	792670265 - 792673854(+)	resistance protein RGA2

TraesSYM2A03G00829670	792679164 - 792680963(+)	Receptor-like kinase family
TraesSYM2A03G00829710	792724770 - 792728735(-)	protein, putative, Ty1-copia
TraesSYM2A03G00829740	792765879 - 792768798(+)	*unknown*
TraesSYM2A03G00829750	792781806 - 792786738(+)	resistance protein RGA2
TraesSYM2A03G00829800	792879278 - 792881875(-)	Protease
TraesSYM2A03G00829810	792928596 - 792934980(+)	Peptidase
TraesSYM2A03G00829820	792935138 - 792938913(-)	transporter NIPA (DUF803)
TraesSYM2A03G00829830	792983207 - 792991299(+)	*unknown*
TraesSYM2A03G00829850	793062876 - 793066852(+)	Putative protein, Ty1-copia
TraesSYM2A03G00830090	793345006 - 793347118(+)	*unknown*
TraesSYM2A03G00830110	793359286 - 793361111(+)	domain-containing protein 78
TraesSYM2A03G00830160	793436071 - 793438232(-)	*unknown*
TraesSYM2A03G00830190	793470556 - 793474718(+)	ATP-dependent RNA helicase
TraesSYM2A03G00830210	793492346 - 793493598(+)	*unknown*
TraesSYM2A03G00830260	793585072 - 793585649(-)	*unknown*
TraesSYM2A03G00830280	793602733 - 793603198(-)	ATPase sarcoplasmic/ERCC-6-like protein
TraesSYM2A03G00830300	793618964 - 793626781(+)	excision repair protein
TraesSYM2A03G00830400	793840966 - 793844595(-)	*unknown*
TraesSYM2A03G00830410	793848007 - 793848435(+)	*unknown*
TraesSYM2A03G00830420	793853596 - 793855385(+)	*unknown*
TraesSYM2A03G00830430	793857902 - 793860706(-)	transcription factor 28
TraesSYM2A03G00830440	793864325 - 793867928(-)	transcription factor 28
TraesSYM2A03G00830450	793882890 - 793884880(+)	transcription factor 28
TraesSYM2A03G00830490	793986412 - 793987375(-)	*unknown*
TraesSYM2A03G00830530	794021318 - 794024342(-)	phosphodiesterases superfamily protein

TraesSYM2A03G00830550	794036223 - 794041272(-)	resistance protein RGA2 - related family
TraesSYM2A03G00830580	794071710 - 794073655(-)	putative protein, Mutator subclass
TraesSYM2A03G00830590	794082632 - 794085547(+)	protein putative, Ty1-copia
TraesSYM2A03G00830600	794135460 - 794136365(-)	SKIP23-like protein
TraesSYM2A03G00830620	794148636 - 794150360(+)	glucosyltransferase 1

Table S8 - Readcounts for the high confidence gene content of the 400kb SY-Mattis interval.

Gene code*	Coordinates (orientation)	Readcounts for dataset 1	Readcounts for dataset 2
TraesSYM2A03G00828360	788728552 - 788738447(+)	1224	1274
TraesSYM2A03G00828370	788768076 - 788769438(+)	0	0
TraesSYM2A03G00828380	788811120 - 788813175(+)	0	0
TraesSYM2A03G00828390	788825472 - 788828016(+)	0	0
TraesSYM2A03G00828400	788828264 - 788832085(+)	1264	1172
TraesSYM2A03G00828410	788833791 - 788837343(+)	2202	2326
TraesSYM2A03G00828420	788837936 - 788839387(+)	8930	6822
TraesSYM2A03G00828440	788841216 - 788842285(+)	0	0
TraesSYM2A03G00828450	788845950 - 788849102(+)	0	0
TraesSYM2A03G00828460	788885045 - 788890732(+)	106	128

Table S9 - Polymorphisms present in the nucleotide sequences of TraesSYM2A03G00828400, TraesSYM2A03G00828410, TraesSYM2A03G00828420, TraesSYM2A03G00828450 and TraesSYM2A03G00828460 for all adapted wheat varieties and Watkins accessions where alignments were generated. Sequences were mapped to SY-Mattis. Scores were taken at five days post inoculation from detached leaves.

			SY-Mattis Chr2A							
			TraesSYM2A03G00828400			*TraesSYM2A03G00828420*		*TraesSYM2A03G00828460*		
Cultivar	*NO6047+ AVR8*	Py 15.1.018	Exon 1 788,828,724	Exon 5 788,830,642	*TraesSYM2A03G00828410*	Exon 1 788,838,013	*TraesSYM2A03G00828450*	788,888,585	788,889,012	788,890,436
Ability	2.33	1.00	-	-	-	-	-	-	T	-
Claire	1.20	1.20	-	-	-	-	-	-	T	-
SY-Epson	1.20	0.00	-	-	-	-	-	-	-	-
Extase			-	-	-	-	-	-	T	-
Cordiale			-	-	-	-	-	-	-	-
Grafton			-	-	-	-	-	-	T	-
Flame	2.33	1.00	-	-	-	-	-	-	T	-
Malacca	3.33	2.00	-	-	-	-	-	-	T	-
Renan	2.00	0.80	-	-	-	-	-	-	-	-
Revelation	1.40	1.20	-	-	-	-	-	-	T	-
Riband	3.00	1.67	-	-	-	-	-	-	T	-
Shango	2.33	1.67	-	-	-	-	-	-	T	-
Spark	0.67	1.33	-	-	-	-	-	-	T	-
SY-Mattis	1.80	1.20	-	-	-	-	-	-	-	-
Wasp	2.00	1.00	-	-	-	-	-	-	T	-
WATDE0048	4.60	5.60	-	-	-	-	-	-	-	-
WATDE0102	2.60	2.20	-	-	-	-	-	-	T	-
WATDE0171	1.80	0.80	-	-	-	-	-	-	T	-
WATDE0310	1.20	0.80	-	-	-	-	-	-	T	-
WATDE0369	3.00	1.30	-	-	-	-	-	-	-	-
WATDE0426		1.00	-	-	-	-	-	T	-	-
WATDE0427	1.00	0.00	-	-	-	-	-	T	-	-
WATDE0428		1.00	-	-	-	-	-	T	-	-
WATDE0465		1.20	-	-	-	-	-	-	-	-
WATDE0477	0.00	0.00	-	A	-	G	-	-	-	-
WATDE0505		1.20	-	-	-	-	-	-	-	-
WATDE0526	1.00	0.67	-	-	-	-	-	-	-	-
WATDE0527	4.60	5.80	-	-	-	-	-	-	-	-
WATDE0541		0.80	-	-	-	-	-	-	-	-
WATDE0546		1.20	-	-	-	-	-	-	-	-
WATDE0566	1.60	2.20	-	-	-	G	-	-	-	-
WATDE0567		1.20	-	-	-	-	-	-	-	-
WATDE0568	5.60	5.80	-	-	-	-	-	-	-	-
WATDE0571	1.80	1.00	-	-	-	-	-	-	-	-
WATDE0592	4.60	5.60	-	-	-	-	-	-	-	-
WATDE0672		1.20	T	-	-	-	-	-	-	-
WATDE0687		0.80	T	-	-	-	-	-	-	-
WATDE0804	0.80	1.80	-	-	-	G	-	-	-	-
WATDE0970		1.00	T*	-	-	-	-	-	-	-
WATDE0971	0.67	0.00	-	-	-	-	-	-	-	-
WATDE0973	0.33	0.30	-	-	-	-	-	-	-	A
WATDE0974		1.00	-	-	-	-	-	-	-	A
WATDE1062		1.20	-	-	-	-	-	-	-	-

* only 1 read coverage

Table S10 - Non-synonymous polymorphisms present in the ORF of *Pm4* for all adapted wheat varieties and Watkins accessions where alignments were generated. The codon position for each polymorphism is shown in the third row. Claire and SY-Mattis were also included. Sequences were mapped to SY-Mattis. Scores were taken at five days post inoculation from detached leaves.

			TraesSYM2A03G00828360 (Pm4)														
			**EXON 1**			**EXON 3**			**EXON 7**								
	**Phenotype**		**A50E**			**E205K**			**W446X**			**V697V**			**A713G**		
**Cultivar**	**NO6047+ *AVR8***	**Py 15.1.018**	**ala - glu**			**glu - lys**			**trp - STOP**			**val - val**			**ala - gly**		
			G	C	A	G	A	A	T	G	G	G	T	C	G	C	C
**Ability**	2.33	1.00	-	-	-	-	-	-	-	-	-	-	-	G	-	G	-
**Claire**	1.20	1.20	-	-	-	-	-	-	-	-	-	-	-	G	-	G	-
**SY-Epson**	1.20	0.00	-	-	-	-	-	-	-	-	-	-	-		-		-
**Flame**	2.33	1.00	-	-	-	-	-	-	-	-	-	-	-	G	-	G	-
**Malacca**	3.33	2.00	-	-	-	-	-	-	-	-	-	-	-	G	-	G	-
**Renan**	2.00	0.80	-	-	-	-	-	-	-	-	-	-	-		-		-
**Revelation**	1.40	1.20	-	-	-	-	-	-	-	-	-	-	-	G	-	G	-
**Riband**	3.00	1.67	-	-	-	-	-	-	-	-	-	-	-	G	-	G	-
**Shango**	2.33	1.67	-	-	-	-	-	-	-	-	-	-	-	G	-	G	-
**Spark**	0.67	1.33	-	-	-	-	-	-	-	-	-	-	-	G	-	G	-
**SY-Mattis**	1.80	1.20	-	-	-	-	-	-	-	-	-	-	-	-	-	-	-
**Wasp**	2.00	1.00	-	-	-	-	-	-	-	-	-	-	-	G	-	G	-
**WATDE0048**	4.60	5.60	-	A	-	A	-	-	-	-	-	-	-	-	-	-	-
**WATDE0102**	2.60	2.20	-	-	-	-	-	-	-	-	-	-	-	G	-	G	-
**WATDE0171**	1.80	0.80	-	-	-	-	-	-	-	-	-	-	-	G	-	G	-
**WATDE0310**	1.20	0.80	-	-	-	-	-	-	-	-	-	-	-	G	-	G	-
**WATDE0369**	3.00	1.30	-	-	-	A	-	-	-	-	-	-	-	-	-	-	-
**WATDE0426**		1.00	-	-	-	A	-	-	-	-	-	-	-	-	-	-	-
**WATDE0427**	1.00	0.00	-	-	-	A	-	-	-	-	-	-	-	-	-	-	-
**WATDE0428**		1.00	-	-	-	A	-	-	-	-	-	-	-	-	-	-	-
**WATDE0465**		1.20	-	-	-	A	-	-	-	-	-	-	-	-	-	-	-
**WATDE0477**	0.00	0.00	-	-	-	A	-	-	-	-	-	-	-	-	-	-	-
**WATDE0505**		1.20	-	-	-	A	-	-	-	-	-	-	-	-	-	-	-
**WATDE0526**	1.00	0.67	-	-	-	A	-	-	-	-	-	-	-	-	-	-	-
**WATDE0527**	4.60	5.80	-	A	-	-	-	-	-	-	-	-	-	-	-	-	-
**WATDE0541**		0.80	-	-	-	A	-	-	-	-	-	-	-	-	-	-	-
**WATDE0546**		1.20	-	-	-	A	-	-	-	-	-	-	-	-	-	-	-
**WATDE0566**	1.60	2.20	-	-	-	A	-	-	-	-	-	-	-	-	-	-	-
**WATDE0567**		1.20	-	-	-	A	-	-	-	-	-	-	-	-	-	-	-
**WATDE0568**	5.60	5.80	-	-	-	A	-	-	-	A	-	-	-	-	-	-	-
**WATDE0571**	1.80	1.00	-	-	-	A	-	-	-	-	-	-	-	-	-	-	-
**WATDE0592**	4.60	5.60	-	-	-	A	-	-	-	A	-	-	-	-	-	-	-
**WATDE0672**		1.20	-	-	-	A	-	-	-	-	-	-	-	-	-	-	-
**WATDE0687**		0.80	-	-	-	A	-	-	-	-	-	-	-	-	-	-	-
**WATDE0804**	0.80	1.80	-	-	-	A	-	-	-	-	-	-	-	-	-	-	-
**WATDE0970**		1.00	-	-	-	A	-	-	-	-	-	-	-	-	-	-	-
**WATDE0971**	0.67	0.00	-	-	-	A	-	-	-	-	-	-	-	-	-	-	-
**WATDE0973**	0.33	0.30	-	-	-	A	-	-	-	-	-	-	-	-	-	-	-
**WATDE0974**		1.00	-	-	-	A	-	-	-	-	-	-	-	-	-	-	-
**WATDE1062**		1.20	-	-	-	A	-	-	-	-	-	-	-	-	-	-	-

Table S11 - Near-isogenic lines (NIL), EMS-induced mutant and overexpression lines used to validate the recognition of *AVR-Rmg8* by *Pm4.*

**Line**	**Description**	**DLA phenotype**				**Reference**
		**NO6047+*Avr8***	**Py 15.1.018**	**Br48ΔeI**	**Br48ΔeI+eI**	
**Bobwhite S26**	Susceptible background for overexpression transformants	S	S	S	S	-
**Federation**	Susceptible background for NILs	S	S	S	S	-
***Fed-Pm4a* NIL**	*Pm4a* from Khapli introgressed into Chancellor, then Federation	R	R	S	R	McIntosh and Bennet, 1979
***Fed-Pm4b* NIL**	*Pm4b* from *T. carthlicum* introgressed into W804, then Federation*. Resistant background for Pm4b* EMS mutants.	R	R	S	R	Briggle, 1966
***Pm4b*_mutant_123**	EMS mutant (G132D) in *Fed-Pm4b* NIL background	S	S	S	S	Sanchéz-Martin et al, 2021
***Pm4b*_mutant_151**	EMS mutant (P184L) in *Fed-Pm4b* NIL background	S	S	S	S	Sanchéz-Martin et al, 2021
***Pm4b*_mutant_207**	EMS mutant (D170N) in *Fed-Pm4b* NIL background	S	S	S	S	Sanchéz-Martin et al, 2021
***Pm4b*_mutant_495_1**	EMS mutant (Q274X) in *Fed-Pm4b* NIL background	S	S	S	S	Sanchéz-Martin et al, 2022
***Pm4b*_mutant_495_3**	EMS mutant (Q274X) in *Fed-Pm4b* NIL background	S	S	S	S	Sanchéz-Martin et al, 2021
***Pm4b*_mutant_526**	EMS mutant (R291K) in *Fed-Pm4b* NIL background	S	S	S	S	Sanchéz-Martin et al, 2021
***Pm4b*_mutant_532**	EMS mutant (G104E) in *Fed-Pm4b* NIL background	S	S	S	S	Sanchéz-Martin et al, 2021
***Pm4b*_mutant_641**	EMS mutant (G45E) in *Fed-Pm4b* NIL background	S	S	S	S	Sanchéz-Martin et al, 2021
***Pm4b*_Nr#3**	*Pm4b* overexpression line	R	R	S	R	Sanchéz-Martin et al, 2021
***Pm4b*_S#3**	Susceptible sister line to *Pm4b*_Nr#3	S	S	S	S	Sanchéz-Martin et al, 2021
***Pm4b*_Nr#52**	*Pm4b* overexpression line	R	R	S	R	Sanchéz-Martin et al, 2021
***Pm4b*_S#52**	Susceptible sister line to *Pm4b*_Nr#52	S	S	S	S	Sanchéz-Martin et al, 2021

Table S12 - Primers used within this study.

Name	Sequence (5' to 3')	Description	Function	Reference
GH414	TAGGTTGGAGAGATCACAACGA	F; Exon 5-6; 179 bp	qRT-PCR *Pm4*_V1 expression	Sanchéz-Martin et al, 2021
GH415	CTGAGGTAGAGGAGGCAACTT	R; Exon 5-6; 179 bp	qRT-PCR *Pm4*_V1 expression	Sanchéz-Martin et al, 2021
GH377	AGAGTGCAGAGACTTCAATCCA	F; Exon 5-7; 159 bp	qRT-PCR *Pm4*_V2 expression	Sanchéz-Martin et al, 2021
GH417	TTCTTCGTACCCAGCAGGTC	R; Exon 5-7; 159 bp	qRT-PCR *Pm4*_V2 expression	Sanchéz-Martin et al, 2021
GH094	TCTCATGGTTGGTCTCGATG	F; Exon 2; 80 bp	qRT-PCR reference gene ADP	Giménez et al, as referenced by Sanchéz-Martin et al, 2021
GH095	GGATGGTGGTGACGATCTCT	R; Exon 2; 80 bp	qRT-PCR reference gene ADP	Giménez et al, as referenced by Sanchéz-Martin et al, 2021
GH105	CAGGCATCTCACTGGAGACT	F; Exon 1; 79 bp	qRT-PCR reference gene ZFL	Sanchéz-Martin et al, 2021
GH106	TGGCATCTCTCTTGCTTCTG	R; Exon 1; 79 bp	qRT-PCR reference gene ZFL	Sanchéz-Martin et al, 2021
P1_F_hex	gaaggtcggagtcaacggatCAAGGCCAACTTCTACCGCT	F	*Pm4* presence/absence KASP (*Pm4*)	This study
P1_F_fam	gaaggtgaccaagttcatgctAAGGCCAACTTCTACCGCA	F	*Pm4* presence/absence KASP (*pm4*)	This study
P1_COM	ACTTGCAGATGCCGTCGA	R	*Pm4* presence/absence KASP	This study

Table S13 – Marker based prediction of the presence of *Pm4* within the Gediflux collection of North western European wheat cultivars. Cultivar information was proved by Luzie Wingen, JIC. Cultivars were genotyped using the '*Pm4* presence absence' KASP primer set (Table S12). In the '*Pm4* Genotype' column: 'Y:Y' indicates the cultivar contains an allele of *Pm4*; 'X:X' indicates the cultivar does not contain an allele of Pm4; '-' indicates insufficient/no DNA for genotyping. In the 'Country of origin' column, 'NL2000' indicates the accession was on the 'Great Britain national list 2000.

**Cultivar**	**Country of origin**	**Release decade**	***Pm4* Genotype**
Alba	Netherlands	1940	X:X
Holdfast	Great Britain	1940	X:X
Juliana	Great Britain	1940	X:X
Lovenik	Netherlands	1940	X:X
Mendel	Netherlands	1940	X:X
Redman	Great Britain	1940	X:X
Stedfast	Great Britain	1940	X:X
Blanco	Sweden	1950	-
Carstens 8	East Germany	1950	X:X
Dr Lassers Dickkopf	Austria	1950	X:X
Loosdorfer Austro Bankut Grannen	Austria	1950	X:X
Minster	Great Britain	1950	X:X
Peragis	East Germany	1950	X:X
Pilot (GB)	Great Britain	1950	X:X
Stam 101	Austria	1950	X:X
Strubes Dickkope	East Germany	1950	-

Svalov Kronen	East Germany	1950	X:X
Tassilo	Austria	1950	X:X
Admonter	Austria	1960	X:X
Apollo (NL)	Netherlands	1960	X:X
Cleo	Netherlands	1960	X:X
Dram Hofner Kolben	Austria	1960	X:X
Elite Leupeuple	Great Britain	1960	X:X
Felix	Netherlands	1960	-
Flevina	Netherlands	1960	X:X
Florian	East Germany	1960	-
Hubertusweisen	Austria	1960	X:X
Hybrid 46	Great Britain	1960	X:X
Ibis	Netherlands	1960	Y:Y
Norda	Belgium	1960	X:X
Pontus	Austria	1960	X:X
Probus	Austria	1960	X:X
Professor Marchal	Great Britain	1960	X:X
Rabe	East Germany	1960	-
Record	Austria	1960	-
Schweigers Taca	Austria	1960	X:X
Stella	Netherlands	1960	-
Thor	Great Britain	1960	X:X
Triumph	Austria	1960	X:X
Almus	West Germany	1970	-
Atou	Great Britain	1970	X:X
Benno	East Germany	1970	X:X
Bouquet	Great Britain	1970	X:X
Cama	Great Britain	1970	-

Clément	France	1970	-
Courtot	France	1970	Y:Y
Cyrano	Netherlands	1970	X:X
Danubius	Austria	1970	X:X
Diplomat	East Germany	1970	-
Extrem	Austria	1970	X:X
Fakir	West Germany	1970	Y:Y
Fanal	West Germany	1970	X:X
Flinor	Great Britain	1970	X:X
Hardi	France	1970	-
Kador	Great Britain	1970	X:X
Kawkas	West Germany	1970	X:X
Kinsman	Great Britain	1970	X:X
Kormoran	East Germany	1970	-
Kranich	Sweden	1970	X:X
Lely	Netherlands	1970	X:X
Maris Freeman	Great Britain	1970	X:X
Maris Nimrod	Great Britain	1970	X:X
Maris Ranger	Great Britain	1970	X:X
Mega	Great Britain	1970	X:X
Mironowskaja	West Germany	1970	X:X
Multiweiss	Austria	1970	X:X
Poros	West Germany	1970	X:X
Solid	Sweden	1970	-
Sportsman	Great Britain	1970	Y:Y
Starke 2	Sweden	1970	X:X
Top	France	1970	X:X
Walde	Sweden	1970	X:X

Winneton	West Germany	1970	X:X
Agron	Austria	1980	X:X
Albatross	Belgium	1980	X:X
Anja	Denmark	1980	X:X
Arkos	West Germany	1980	X:X
Avalon	Great Britain	1980	X:X
Beauchamp	France	1980	X:X
Bounty	Great Britain	1980	X:X
Brigand	Great Britain	1980	X:X
Brimstone	Great Britain	1980	-
Brock	Great Britain	1980	X:X
Calif	Germany	1980	X:X
Camp Rémy	France	1980	X:X
Capitole	Belgium	1980	-
Compal	West Germany	1980	Y:Y
David	Austria	1980	-
Fenman	Great Britain	1980	X:X
Festival	France	1980	X:X
Fidel	France	1980	X:X
Folke	Sweden	1980	X:X
Fontus	Austria	1980	-
Gamin	Belgium	1980	X:X
Granada	Netherlands	1980	X:X
Granta	Netherlands	1980	X:X
Helge	Sweden	1980	X:X
Hubertus	Austria	1980	-
Hustler	Great Britain	1980	X:X
Iena	Belgium	1980	X:X

Karat	Austria	1980	X:X
Longbow	Great Britain	1980	-
Martin	Austria	1980	X:X
Mission	Great Britain	1980	-
Moulin	France	1980	X:X
Norman	Great Britain	1980	-
Odeon	Belgium	1980	X:X
Perlo	Austria	1980	-
Pernel	France	1980	Y:Y
Rapier	Great Britain	1980	X:X
Regent	Austria	1980	X:X
Scipion	France	1980	X:X
Slejpner	Sweden	1980	X:X
Sperber	East Germany	1980	X:X
Stetson	Great Britain	1980	X:X
Taras	West Germany	1980	X:X
Titus	Austria	1980	X:X
Virtue	Great Britain	1980	X:X
Zemon	Belgium	1980	X:X
Abbot	Great Britain	1990	X:X
Altria	France	1990	-
Aztec	France	1990	X:X
Borenos	West Germany	1990	X:X
Brigadier	Great Britain	1990	Y:Y
Buchan	Great Britain	1990	Y:Y
Buzzard	Germany	1990	X:X
Cadenza	Great Britain	1990	X:X
Capo	Austria	1990	X:X

Cezanne	France	1990	X:X
Charger	France	1990	X:X
Claudius	Austria	1990	X:X
Contra	Germany	1990	Y:Y
Equinox	Great Britain	1990	X:X
Expert	Austria	1990	X:X
Faktor	Germany	1990	Y:Y
Flair	Germany	1990	X:X
Flame	Great Britain	1990	Y:Y
Florida	Denmark	1990	-
Genesis	Great Britain	1990	X:X
Georg	Austria	1990	X:X
Grief	Germany	1990	X:X
Haven	Great Britain	1990	X:X
Hunter	Great Britain	1990	X:X
Ibis (D)	Germany	1990	X:X
Ikarus	Austria	1990	-
Isengrain	France	1990	X:X
Kontrast	Germany	1990	X:X
Lindos	Austria	1990	Y:Y
Mikon	Germany	1990	X:X
Napier	Great Britain	1990	X:X
Obelisk	Germany	1990	X:X
Optimus	Austria	1990	X:X
Pallus	Germany	1990	X:X
Pastiche	Great Britain	1990	X:X
Pegassos	Germany	1990	X:X
Pepital	Denmark	1990	X:X

Renan	Austria	1990	Y:Y
Riband	Great Britain	1990	Y:Y
Ritmo	Germany	1990	X:X
Savannah	Great Britain	1990	Y:Y
Shamrock	Great Britain	1990	X:X
Sideral	France	1990	-
Silvius	Austria	1990	-
Spark	Great Britain	1990	Y:Y
Tambor	Austria	1990	X:X
Torfrida	Great Britain	1990	Y:Y
Toronto	Germany	1990	Y:Y
Trémie	France	1990	X:X
Zentra	Germany	1990	X:X
Ability	Great Britain (NL2000)	2000	Y:Y
Access	Great Britain (NL2000)	2000	Y:Y
Admiral	Great Britain (NL2000)	2000	X:X
Adroit	Great Britain (NL2000)	2000	Y:Y
Alcier	Great Britain (NL2000)	2000	Y:Y
Alert	Great Britain (NL2000)	2000	X:X
Ambassador	Great Britain (NL2000)	2000	X:X
Andante	Great Britain (NL2000)	2000	X:X
Anthem	Great Britain (NL2000)	2000	X:X
Apostle	Great Britain (NL2000)	2000	X:X
Aristocrat	Great Britain (NL2000)	2000	X:X
Assett	Great Britain (NL2000)	2000	-
Athlet	Great Britain (NL2000)	2000	X:X
Atla	Great Britain (NL2000)	2000	X:X
Attoll	Great Britain (NL2000)	2000	X:X

Avocet	Great Britain (NL2000)	2000	X:X
Axial	Great Britain (NL2000)	2000	X:X
Bandit	Great Britain (NL2000)	2000	X:X
Banner	Great Britain (NL2000)	2000	X:X
Baron	Great Britain (NL2000)	2000	X:X
Beaufort	Great Britain (NL2000)	2000	X:X
Belplaine	Great Britain (NL2000)	2000	X:X
Bercy	Great Britain (NL2000)	2000	X:X
Bert	Great Britain (NL2000)	2000	X:X
Biscay	Great Britain (NL2000)	2000	Y:Y
Blitz	Great Britain (NL2000)	2000	X:X
Bourbon	Great Britain (NL2000)	2000	X:X
Boxer	Great Britain (NL2000)	2000	Y:Y
Breval	Great Britain (NL2000)	2000	X:X
Brutus	Great Britain (NL2000)	2000	X:X
Bryden	Great Britain (NL2000)	2000	Y:Y
Bullet	Great Britain (NL2000)	2000	X:X
Buster	Great Britain (NL2000)	2000	-
Cambat	Great Britain (NL2000)	2000	-
Caprimus	Great Britain (NL2000)	2000	X:X
Captor	Great Britain (NL2000)	2000	X:X
Carolus	Great Britain (NL2000)	2000	X:X
Catamaran	Great Britain (NL2000)	2000	X:X
Caxton	Great Britain (NL2000)	2000	X:X
Cheetah	Great Britain (NL2000)	2000	X:X
Chianti	Great Britain (NL2000)	2000	Y:Y
Civic	Great Britain (NL2000)	2000	X:X
Claire	Great Britain (NL2000)	2000	Y:Y

Clove	Great Britain (NL2000)	2000	X:X
Club	Great Britain (NL2000)	2000	X:X
Cobalt	Great Britain (NL2000)	2000	X:X
Commodore	Great Britain (NL2000)	2000	X:X
Consort	Great Britain (NL2000)	2000	Y:Y
Contour	Great Britain (NL2000)	2000	X:X
Corinthian	Great Britain (NL2000)	2000	X:X
Corsaire	Great Britain (NL2000)	2000	X:X
Coxwain	Great Britain (NL2000)	2000	X:X
Creneau	Great Britain (NL2000)	2000	X:X
Crest	Great Britain (NL2000)	2000	Y:Y
Daphne	Great Britain (NL2000)	2000	X:X
Dean	Great Britain (NL2000)	2000	X:X
Deben	Great Britain (NL2000)	2000	Y:Y
Denver	Great Britain (NL2000)	2000	X:X
Depot	Great Britain (NL2000)	2000	X:X
Destroyer	Great Britain (NL2000)	2000	Y:Y
Diablo	Great Britain (NL2000)	2000	-
Dorby	Great Britain (NL2000)	2000	-
Drake	Great Britain (NL2000)	2000	X:X
Druid	Great Britain (NL2000)	2000	X:X
Dynamo	Great Britain (NL2000)	2000	X:X
Eagle	Great Britain (NL2000)	2000	-
Emblem	Great Britain (NL2000)	2000	Y:Y
Encore	Great Britain (NL2000)	2000	Y:Y
Erland	Great Britain (NL2000)	2000	X:X
Estica	Great Britain (NL2000)	2000	X:X
Estorial	Great Britain (NL2000)	2000	X:X

Fenda	Great Britain (NL2000)	2000	X:X
Feuvert	Great Britain (NL2000)	2000	X:X
Flash	Great Britain (NL2000)	2000	Y:Y
Fletum	Great Britain (NL2000)	2000	-
Focus	Great Britain (NL2000)	2000	X:X
Foreman	Great Britain (NL2000)	2000	X:X
Fortress	Great Britain (NL2000)	2000	X:X
Fresco	Great Britain (NL2000)	2000	X:X
Frista	Great Britain (NL2000)	2000	X:X
Fromendor	Great Britain (NL2000)	2000	X:X
Gallatea	Great Britain (NL2000)	2000	X:X
Galliard	Great Britain (NL2000)	2000	X:X
Gambit	Great Britain (NL2000)	2000	X:X
Gondola	Great Britain (NL2000)	2000	X:X
Governor	Great Britain (NL2000)	2000	X:X
Guardian	Great Britain (NL2000)	2000	X:X
Hanno	Great Britain (NL2000)	2000	X:X
Harrier	Great Britain (NL2000)	2000	Y:Y
Heinrich	Great Britain (NL2000)	2000	-
Hickory	Great Britain (NL2000)	2000	Y:Y
Holster	Great Britain (NL2000)	2000	Y:Y
Hudson	Great Britain (NL2000)	2000	Y:Y
Hussar	Great Britain (NL2000)	2000	-
Imola	Great Britain (NL2000)	2000	X:X
Jubilatka	Great Britain (NL2000)	2000	X:X
Kontiki	Great Britain (NL2000)	2000	X:X
Kronjewel	Great Britain (NL2000)	2000	Y:Y
Kyalami	Great Britain (NL2000)	2000	X:X

Lancelot	Great Britain (NL2000)	2000	X:X
Legend	Great Britain (NL2000)	2000	Y:Y
Leo	Great Britain (NL2000)	2000	X:X
Lynx	Great Britain (NL2000)	2000	X:X
Madrigal	Great Britain (NL2000)	2000	X:X
Magellan	Great Britain (NL2000)	2000	Y:Y
Malacca	Great Britain (NL2000)	2000	Y:Y
Mandate	Great Britain (NL2000)	2000	X:X
Mantle	Great Britain (NL2000)	2000	X:X
Mars	Great Britain (NL2000)	2000	X:X
Meteor	Great Britain (NL2000)	2000	X:X
Morell	Great Britain (NL2000)	2000	Y:Y
Motto	Great Britain (NL2000)	2000	X:X
Newhaven	Great Britain (NL2000)	2000	X:X
Norsman	Great Britain (NL2000)	2000	X:X
Option	Great Britain (NL2000)	2000	X:X
Orqual	Great Britain (NL2000)	2000	X:X
Ostara	Great Britain (NL2000)	2000	X:X
Parade	Great Britain (NL2000)	2000	X:X
Patience	Great Britain (NL2000)	2000	X:X
Peacock	Great Britain (NL2000)	2000	Y:Y
Piccadilly	Great Britain (NL2000)	2000	X:X
Pistol	Great Britain (NL2000)	2000	X:X
Poet	Great Britain (NL2000)	2000	X:X
Profet	Great Britain (NL2000)	2000	X:X
Profi	Great Britain (NL2000)	2000	Y:Y
Prospect	Great Britain (NL2000)	2000	X:X
Puma	Great Britain (NL2000)	2000	Y:Y

Raleigh	Great Britain (NL2000)	2000	X:X
Reaper	Great Britain (NL2000)	2000	X:X
Rebel	Great Britain (NL2000)	2000	-
Renard	Great Britain (NL2000)	2000	X:X
Rendezvous	Great Britain (NL2000)	2000	Y:Y
Renown	Great Britain (NL2000)	2000	X:X
Rhino	Great Britain (NL2000)	2000	Y:Y
Rialto	Great Britain (NL2000)	2000	X:X
Rifle	Great Britain (NL2000)	2000	X:X
Ritz	Great Britain (NL2000)	2000	X:X
Rocket	Great Britain (NL2000)	2000	X:X
Rooster	Great Britain (NL2000)	2000	Y:Y
Rostrum	Great Britain (NL2000)	2000	X:X
Rubens	Great Britain (NL2000)	2000	X:X
Russett	Great Britain (NL2000)	2000	Y:Y
Sabre	Great Britain (NL2000)	2000	X:X
Samson	Great Britain (NL2000)	2000	X:X
Sarek	Great Britain (NL2000)	2000	X:X
Sarsen	Great Britain (NL2000)	2000	Y:Y
Saxon	Great Britain (NL2000)	2000	X:X
Semper	Great Britain (NL2000)	2000	X:X
Sennet	Great Britain (NL2000)	2000	X:X
Shango	Great Britain (NL2000)	2000	Y:Y
Shannon	Great Britain (NL2000)	2000	X:X
Sickle	Great Britain (NL2000)	2000	Y:Y
Sirius	Great Britain (NL2000)	2000	X:X
Sitka	Great Britain (NL2000)	2000	X:X
Sniper	Great Britain (NL2000)	2000	X:X

Soleil	Great Britain (NL2000)	2000	X:X
Solstice	Great Britain (NL2000)	2000	X:X
Spice	Great Britain (NL2000)	2000	X:X
Spitfire	Great Britain (NL2000)	2000	Y:Y
Spray	Great Britain (NL2000)	2000	X:X
Squadron	Great Britain (NL2000)	2000	Y:Y
Stag	Great Britain (NL2000)	2000	X:X
Stallion	Great Britain (NL2000)	2000	X:X
Tallon	Great Britain (NL2000)	2000	X:X
Tandero	Great Britain (NL2000)	2000	X:X
Tanker	Great Britain (NL2000)	2000	X:X
Tara	Great Britain (NL2000)	2000	X:X
Tessa	Great Britain (NL2000)	2000	X:X
Texel	Great Britain (NL2000)	2000	X:X
Thunder	Great Britain (NL2000)	2000	X:X
Tilburi	Great Britain (NL2000)	2000	X:X
Tjalk	Great Britain (NL2000)	2000	X:X
Token	Great Britain (NL2000)	2000	X:X
Tomo	Great Britain (NL2000)	2000	Y:Y
Torch	Great Britain (NL2000)	2000	X:X
Toucan	Great Britain (NL2000)	2000	Y:Y
Trader	Great Britain (NL2000)	2000	X:X
Trafalgar	Great Britain (NL2000)	2000	X:X
Trawler	Great Britain (NL2000)	2000	X:X
Trend	Great Britain (NL2000)	2000	Y:Y
Turpin	Great Britain (NL2000)	2000	Y:Y
Vauntless	Great Britain (NL2000)	2000	-
Veritas	Great Britain (NL2000)	2000	X:X

Victo	Great Britain (NL2000)	2000	-
Vivant	Great Britain (NL2000)	2000	X:X
Vocal	Great Britain (NL2000)	2000	X:X
Voyage	Great Britain (NL2000)	2000	X:X
Warrior	Great Britain (NL2000)	2000	-
Wasp	Great Britain (NL2000)	2000	Y:Y
Welton	Great Britain (NL2000)	2000	X:X
Wizard	Great Britain (NL2000)	2000	X:X
Woodstock	Great Britain (NL2000)	2000	X:X
Wykenham	Great Britain (NL2000)	2000	Y:Y
Xi 19	Great Britain (NL2000)	2000	X:X
Yacht	Great Britain (NL2000)	2000	X:X
Zodiac	Great Britain (NL2000)	2000	X:X
Banco	-	-	X:X
Bledor	Belgium	-	X:X
Capitaine	-	-	X:X
Capitole	-	-	X:X
Caribo	-	-	X:X
Combat	-	-	X:X
Dauntless	-	-	Y:Y
Ergo	Sweden	-	X:X
Eroica	Sweden	-	X:X
Eroica II	Sweden	-	X:X
Ertus	Sweden	-	X:X
Jarl	Sweden	-	X:X
Meredien	Sweden	-	-
Odin	Sweden	-	-
Probstdorfer Stabil	-	-	X:X

Roi Albert	Belgium	-	X:X
Rufus	Belgium	-	X:X
Schreibers Sturmweizen	-	-	X:X
Skandia	Sweden	-	X:X
Stava	Sweden	-	Y:Y
Svale	Sweden	-	X:X
Svalov 0987	-	-	X:X
Terra	Sweden	-	X:X
Virgo	Sweden	-	X:X
Virtus	Sweden	-	X:X
William	Sweden	-	Y:Y
Flair Eigeno Nachbau	Germany?	?	X:X
Glicevka	Germany?	?	-
Bersee	Great Britain	1940-50	X:X
Criewener 152	East Germany	1940-50	X:X
Ebersbacher Weiss	East Germany	1940-50	X:X
Heine 4	East Germany	1940-50	X:X
Kadolzer	Austria	1940-50	X:X
Mahndorfer Tempo	East Germany	1940-50	X:X
Rimpaus Bastard 2	East Germany	1940-50	-
Rimpaus Bastard 2	-	1940-50	-
Rimpaus Braun	East Germany	1940-50	-
Ritzlhofer Neu	Austria	1940-50	X:X
Salzmunder Standard	East Germany	1940-50	X:X
Starling	Great Britain	1940-50	X:X
Svalon 0907	East Germany	1940-50	-
TscherWaks Bergrannter Machfeld	Austria	1940-50	X:X
Vague d'épis	France	1940-50	X:X

Vilmorlin 27	France	1940-50	X:X
Warden	Great Britain	1940-50	X:X
Cappelle Desprez	France	1950-60	X:X
Carstens		1950-60	X:X
Carstens	Netherlands	1950-60	X:X
Eros	West Germany	1950-60	X:X
Etoile de Choisy	France	1950-60	X:X
Flamingo	Great Britain	1950-60	X:X
Heine 7	East Germany	1950-60	X:X
Hochland	West Germany	1950-60	X:X
Leda	Netherlands	1950-60	X:X
Muck	West Germany	1950-60	X:X
Triumph (NC)	Netherlands	1950-60	X:X
Vilmorin 53	France	1950-60	X:X
Werla	East Germany	1950-60	X:X
Capitole	France	1960-70	-
Champlein	France	1960-70	X:X
Erla Kolben	Austria	1960-70	X:X
Joss Cambier	France	1960-70	X:X
Jubilar	East Germany	1960-70	X:X
Manella	Netherlands	1960-70	X:X
Maris Widgeon	Great Britain	1960-70	X:X
Moisson	France	1960-70	X:X
Orlando	West Germany	1960-70	X:X
Remois	France	1960-70	X:X
Starke	Sweden	1960-70	X:X
Tadorna	Netherlands	1960-70	X:X
Adam	Austria	1970-80	X:X

Alcedo	West Germany	1970-80	X:X
Aquila	Great Britain	1970-80	X:X
Armada	Great Britain	1970-80	Y:Y
Arminda	France	1970-80	X:X
Capiso	-	1970-80	-
Disponent	East Germany	1970-80	X:X
Flanders	Great Britain	1970-80	X:X
Hildur	Sweden	1970-80	X:X
Hobbit	Great Britain	1970-80	X:X
Holme	Sweden	1970-80	X:X
Lutin	France	1970-80	X:X
Mardler	Great Britain	1970-80	X:X
Maris Huntsman	Great Britain	1970-80	X:X
Micronowskata	West Germany	1970-80	X:X
Nautica	Netherlands	1970-80	X:X
Oenus	Austria	1970-80	X:X
Okapi	East Germany	1970-80	-
Talent	France	1970-80	X:X
Vuka	East Germany	1970-80	X:X
Apollo (D)	East Germany	1980-90	X:X
Beaver	Great Britain	1980-90	X:X
Escoria	Belgium	1980-90	X:X
Galahad	Great Britain	1980-90	X:X
Gawain	Denmark	1980-90	X:X
Hereward	Great Britain	1980-90	X:X
Hornet	Great Britain	1980-90	X:X
Kanzler	East Germany	1980-90	X:X
Kosak	Sweden	1980-90	Y:Y

Mercia	Great Britain	1980-90	X:X
Miras	West Germany	1980-90	X:X
Recital	France	1980-90	-
Regina	West Germany	1980-90	X:X
Rektor	East Germany	1980-90	X:X
Soissons	France	1980-90	X:X
Thesee	France	1980-90	-
Urban	Denmark	1980-90	-
Cama	Belgium	-	X:X
Celesta	Belgium	-	X:X
Chinese Spring	China	-	X:X
Clovis	Belgium	-	Y:Y
Hesbinion	Belgium	-	X:X
Jason	Belgium	-	X:X
Marco	Belgium	-	X:X
Marisa	Belgium	-	X:X
Mina	Belgium	-	X:X
Mutant Odeon	Belgium	-	X:X
Mutant Odeon II	Belgium	-	X:X
Orestis	Germany	-	X:X
Pony	Belgium	-	X:X
Prima	Belgium	-	X:X
Stella	Belgium	-	-

Table S14 - Watkins accessions and adapted wheat varieties tested within this study that carry *Pm4.* Scores were taken from detached leaves at six-days post inoculation.

Line	*Pm4* allele	NO6047+*AVR8* (6 dpi)	Py 15.1.018 (6 dpi)
Ability	*Pm4b*	2.33	1.00
Claire		1.20	1.20
Flame		2.33	1.00
Malacca		3.33	2.00
Revelation		1.40	1.20
Riband		3.00	1.67
Shango		2.33	1.67
Spark		0.67	1.33
Wasp		2.00	1.00
WATDE0102		2.60	2.20
WATDE0171		1.80	0.80
WATDE0310		1.20	0.80
SY-Epson*	*Pm4d*	1.20	0.00
Renan*		2.00	0.80
SY-Mattis*		1.80	1.20
CDC Stanley*		2.60	1.20
WATDE0369	*Pm4f*	3.00	1.30
WATDE0426			1.00
WATDE0427		1.00	0.00
WATDE0428			1.00

WATDE0465			1.20
WATDE0477		0.00	0.00
WATDE0505			1.20
WATDE0526		1.00	0.67
WATDE0541			0.80
WATDE0546			1.20
WATDE0566		1.60	2.20
WATDE0567			1.20
WATDE0571		1.80	1.00
WATDE0672			1.20
WATDE0687			0.80
WATDE0804		0.80	1.80
WATDE0970			1.00
WATDE0971		0.67	0.00
WATDE0973		0.33	0.30
WATDE0974			1.00
WATDE1062			1.20
WATDE0048	*Pm4i*	4.60	5.60
WATDE0527		4.60	5.80
WATDE0568	*Pm4j*	5.60	5.80
WATDE0592		4.60	5.60

* contains the *Ae. ventricosa* 2NS translocation

Table S15 - BR48 isogenic series detached leaf and spike assay scores. () indicate which *Pm4* allele the line contains. Scores were taken at five or six days post inoculation.

Accession (*Pm4* allele)	Mean detached leaf assay score				Detached spike assay	
	**BR48ΔeI**	**BR48ΔeI+eI**	**BR48ΔeI+eII**	**BR48ΔeI+eII'**	**BR48ΔeI**	**BR48ΔeI+eI**
Bobwhite	4.00	4.00	4.25	5.75	6.00	6.00
Federation	5.75	5.50	5.50	6.00	6.00	6.00
Fed-Pm4a-NIL	5.75	1.75	0.75	1.75	6.00	5.00
Fed-Pm4b-NIL	5.75	1.25	1.50	2.75	6.00	4.00
Nr#3	3.50	1.25	1.75	1.50	6.00	5.67
Nr#52	4.00	1.25	1.00	2.25	5.67	2.00
S3	3.75	4.00	4.25	5.00	6.00	6.00
S52	3.50	3.75	3.75	3.50	6.00	6.00
*Pm4b*_Mut_207	5.50	6.00	5.50	6.00	6.00	6.00
*Pm4b*_Mut_495-3	5.50	5.50	6.00	6.00	6.00	6.00
*Pm4b*_Mut_526	4.25	4.25	4.00	4.25	6.00	6.00
*Pm4b*_Mut_641	5.50	3.50	4.75	4.50	6.00	6.00
WATDE0102(*Pm4b*)	1.50	0.50	0.00	0.75	-	-
WATDE0310(*Pm4b*)	3.75	1.25	1.00	0.25	-	-
SY-Mattis(*Pm4d*)	3.75	1.25	0.00	1.00	-	-
CDC Stanley(*Pm4d*)	3.25	1.00	0.75	1.50	-	-
VPM1(*Pm4d*)	3.50	1.00	0.25	1.25	-	-
WATDE0427(*Pm4f*)	3.75	1.50	1.25	1.00	-	-
WATDE0477(*Pm4f*)	4.50	1.25	0.25	2.00	-	-
WATDE0571(*Pm4f*)	4.50	0.75	0.50	1.25	-	-
WATDE0048(*Pm4i*)	2.00	1.75	1.00	2.50	-	-

WATDE0527(*Pm4i*)	2.75	1.75	1.00	3.50	-	-
WATDE0568(*Pm4j*)	4.75	4.50	4.75	5.75	-	-
WATDE0592(*Pm4j*)	5.50	5.25	5.00	5.75	-	-
WW-093(*Pm4g*)	2.75	3.00	3.25	4.25	-	-
WW-213(*Pm4g*)	3.75	3.50	4.00	5.25	-	-
WW-470(*Pm4g*)	5.00	4.50	5.50	5.75	-	-
WW-474(*Pm4h*)	3.00	1.63	1.75	2.13	-	-

Table S16 - Comparison of disease phenotypes of the different *Pm4* alleles against *MoT* and *Bgt* isolates. *MoT* phenotypes were assigned by comparing the detached leaf phenotype data for isolate BR48ΔeI relative to isolates BR48ΔeI+eI, BR48ΔeI+eII and BR48ΔeI+eII' for each *Pm4* allele. *Bgt* phenotype data was taken from Supplementary table 5 from Sánchez-Martín et al. 2021. The four *Bgt* isolates that were avirulent on *Fed-Pm4a*-NIL and *Fed-Pm4b*-NIL were assumed to contain *Avr-Pm4*. The consensus phenotype for the different *Pm4* alleles against the *Bgt* isolates is recorded. 'R' = resistant; 'MR' = moderate resistance; 'S' = susceptible; 'R/S' = some accessions carrying a given allele showed either resistant or susceptible phenotypes so a consensus phenotype could not be given.

	MoT				***Bgt***			
	**BR48ΔeI**	**BR48ΔeI+eI**	**BR48ΔeI+eII**	**BR48ΔeI+eII'**	**Bgt94202**	**Bgt96224**	**Bgt97223**	**Bgt97266**
Pm4a	S	R	R	R	R	R	R	R
Pm4b	S	R	R	R	R	R	R	R
Pm4d	S	R	R	R	R	R	R	R
Pm4f	S	R	R	R	S	S	S	R/S
Pm4g	S	S	S	S	S	S	S	R/S
Pm4h	S	R	R	R	R	R	R	R
Pm4i	MR	R	R	MR	no data			
Pm4j	S	S	S	S				

Table S17 - Marker based prediction of the presence of *Pm4* within a selection of 565 CIMMYT wheat lines. Cultivars were genotyped for *Pm4* using the '*Pm4* presence/absence' KASP primer set (Table S12) and the 2NS translocation originating from wheat line VPM1 using marker set CIMwMAS0004 (Helguera et al. 2003). In the 'AVR-Rmg8-R/S' column: 'T:T' indicates the line contains an allele of *Pm4*; 'A:A' indicates the line does not contain an allele of *Pm4*. In the '2NS' column: 'T:T' indicates the line contains the VPM1 translocation; 'C:C' indicates the line does not contain the VPM1 translocation; 'C:T' indicates the line is heterozygous for this marker. The 'CIMwMAS' codes correspond to the CIMMYT marker ID.

SampleID	Pedigree	Marker set	
		AVR-Rmg8-R/S	2NS
		CIMwMAS1217	CIMwMAS0004
BW22GS018742	NINGA #1	A:A	T:T
BW22GS018743	BORL14//BECARD/QUAIU #1	A:A	T:T
BW22GS018744	MUCUY*2//SUP152/BAJ #1	A:A	T:T
BW22GS018745	BORL14//SUP152/FRNCLN/3/KASUKO	A:A	T:T
BW22GS018746	BORL14/9/WAXWING/7/TNMU/6/CEP80111/CEP81165/5/IAC5/4/YKT406/3/AG/ASN//ATR/8/ATTILA/3*BCN//BAV 92/3/TILHI/4/SHA7/VEE#5//ARIV92/10/KASUKO	A:A	T:T
BW22GS018747	MUNAL #1/CHIPAK//KASUKO	A:A	T:T
BW22GS018748	AKEPA/BOKOTA/3/BORL14//KFA/2*KACHU	A:A	T:T
BW22GS018749	CHIPAK*2/4/KACHU/3/WHEAR//2*PRL/2*PASTOR	A:A	T:T
BW22GS018750	CHIPAK*2/4/KACHU/3/WHEAR//2*PRL/2*PASTOR	A:A	T:T
BW22GS018751	MUCUY/3/KACHU//KIRITATI/2*TRCH/4/MOKUE #1	A:A	T:T
BW22GS018752	WHEAR//2*PRL/2*PASTOR/3/QUAIU #1/4/SWSR22T.B.//TACUPETO F2001*2/BRAMBLING/3/2*TACUPETO F2001*2/BRAMBLING/5/BORL14	A:A	T:T
BW22GS018753	KISKADEE #1/5/KAUZ*2/MNV//KAUZ/3/MILAN/4/BAV92/6/WHEAR//2*PRL/2*PASTOR/7/MUTUS/DANPHE #1/4/C80.1/3*BATAVIA//2*WBLL1/3/C80.1/3*QT4522//2*PASTOR/8/MUNAL #1/CIRO16	A:A	T:T
BW22GS018754	ISENGRAIN/KBIRD//MUNAL #1/3/SUP152/KENYA SUNBIRD/4/FRNCLN*2/KINGBIRD #1	A:A	T:T
BW22GS018755	KACHU/SAUAL/3/TACUPETO F2001/BRAMBLING//KIRITATI*2/4/FRET2/TUKURU//FRET2/3/MUNAL #1	A:A	T:T
BW22GS018756	TRCH/3/ROLF07/YANAC//TACUPETO F2001/BRAMBLING/4/PRL/2*PASTOR/5/BORL14/6/KASUKO	A:A	T:T
BW22GS018757	TRCH/7/TUKURU//BAV92/RAYON/6/NG8201/KAUZ/4/SHA7//PRL/VEE#6/3/FASAN/5/MILAN/KAUZ/8/CIRO16/9/BORL14/10/MOKUE #1	A:A	T:T
BW22GS018758	NL971*2/4/HUW234+LR34/PRINIA//INQALAB 91*2/KUKUNA/3/FRET2*2/SHAMA*2/5/MUCUY	A:A	T:T
BW22GS018759	FRANCOLIN #1/3/PBW343*2/KUKUNA*2//YANAC/4/KINGBIRD #1//INQALAB 91*2/TUKURU*2/5/MUNAL #1	A:A	T:T

BW22GS018760	FRANCOLIN #1/3/PBW343*2/KUKUNA*2//YANAC/4/KINGBIRD #1//INQALAB 91*2/TUKURU*2/5/KINGBIRD #1//INQALAB 91*2/TUKURU	A:A	T:T
BW22GS018761	FRANCOLIN #1/3/PBW343*2/KUKUNA*2//YANAC/4/KINGBIRD #1//INQALAB 91*2/TUKURU*2/5/KINGBIRD #1//INQALAB 91*2/TUKURU	A:A	T:T
BW22GS018762	FRANCOLIN #1/3/PBW343*2/KUKUNA*2//YANAC/4/KINGBIRD #1//INQALAB 91*2/TUKURU*2/5/CHIPAK	A:A	T:T
BW22GS018763	CAL/NH//H567.71/3/SERI/4/CAL/NH//H567.71/5/2*KAUZ/6/WH576/7/WH 542/8/WAXWING/9/ATTILA*2/PBW65//PIHA/3/ATTILA/2*PASTOR/10/UP2338*2/KKTS*2//YANAC/11/BORL14/12 /KASUKO	A:A	T:T
BW22GS018764	ATTILA*2/PBW65/5/CNO79//PF70354/MUS/3/PASTOR/4/BAV92/6/KINGBIRD #1/7/COPIO*2/8/BORL14	A:A	T:T
BW22GS018765	KACHU/SUP152//KASUKO/3/KASUKO	A:A	T:T
BW22GS018766	KASUKO*2/4/BECARD/AKURI*2/3/PBW343*2/KUKUNA*2//FRTL/PIFED	A:A	T:T
BW22GS018767	KASUKO*2/CHIPAK	A:A	T:T
BW22GS018768	KASUKO*2/CHIPAK	A:A	T:T
BW22GS018769	KIRITATI//PRL/2*PASTOR/5/OASIS/SKAUZ//4*BCN/3/PASTOR/4/KAUZ*2/YACO//KAUZ/6/KIRITATI//PRL/2*PASTOR /7/PBW343*2/KUKUNA*2//FRTL/PIFED*2/8/KASUKO	A:A	T:T
BW22GS018770	BORL14/MOKUE #1	A:A	T:T
BW22GS018771	BORL14/7/KFA/2*KACHU/5/WBLL1*2/4/BABAX/LR42//BABAX/3/BABAX/LR42//BABAX/6/KFA/2*KACHU	A:A	T:T
BW22GS018772	BORL14/KASUKO	A:A	T:T
BW22GS018773	BORL14/KASUKO	A:A	T:T
BW22GS018774	BORL14/KASUKO	A:A	T:T
BW22GS018775	BORL14/KASUKO	A:A	T:T
BW22GS018776	BORL14/6/KSW/SAUAL//SAUAL/3/TRCH/HUIRIVIS #1/5/UP2338*2/SHAMA/3/MILAN/KAUZ//CHIL/CHUM18/4/UP2338*2/SHAMA	A:A	T:T
BW22GS018777	BORL14/6/KSW/SAUAL//SAUAL/3/TRCH/HUIRIVIS #1/5/UP2338*2/SHAMA/3/MILAN/KAUZ//CHIL/CHUM18/4/UP2338*2/SHAMA	A:A	T:T
BW22GS018778	BORL14/6/KSW/SAUAL//SAUAL/3/TRCH/HUIRIVIS #1/5/UP2338*2/SHAMA/3/MILAN/KAUZ//CHIL/CHUM18/4/UP2338*2/SHAMA	A:A	T:T
BW22GS018779	BORL14/3/KFA/2*KACHU//KACHU/KIRITATI	A:A	T:T
BW22GS018780	MUNAL #1/KASUKO	A:A	T:T
BW22GS018781	KINGBIRD #1//INQALAB 91*2/TUKURU/3/KASUKO	A:A	T:T
BW22GS018782	KINGBIRD #1//INQALAB 91*2/TUKURU/3/KASUKO	A:A	T:T
BW22GS018783	KINGBIRD #1//INQALAB 91*2/TUKURU/3/KASUKO	A:A	T:T
BW22GS018784	KINGBIRD #1//INQALAB 91*2/TUKURU/6/KSW/SAUAL//SAUAL/3/TRCH/HUIRIVIS #1/5/UP2338*2/SHAMA/3/MILAN/KAUZ//CHIL/CHUM18/4/UP2338*2/SHAMA	A:A	T:T

BW22GS018785	CHIPAK/KASUKO	A:A	T:T
BW22GS018786	MUCUY/3/BORL14*2//KFA/2*KACHU	A:A	T:T
BW22GS018787	KACHU//KIRITATI/2*TRCH/3/KASUKO	A:A	T:T
BW22GS018788	CHIBIA//PRLII/CM65531/3/FISCAL/4/DANPHE #1/5/CHIBIA//PRLII/CM65531/3/MISR 2/6/KASUKO	A:A	T:T
BW22GS018789	CHIBIA//PRLII/CM65531/3/FISCAL/4/DANPHE #1/5/CHIBIA//PRLII/CM65531/3/MISR 2/6/KASUKO	A:A	T:T
BW22GS018790	TAM200/PASTOR//TOBA97/3/FRNCLN/4/WHEAR//2*PRL/2*PASTOR/5/KASUKO	A:A	T:T
BW22GS018791	TAM200/PASTOR//TOBA97/3/FRNCLN/4/WHEAR//2*PRL/2*PASTOR/5/KASUKO	A:A	T:T
BW22GS018792	SUP152/FRNCLN//KASUKO	A:A	T:T
BW22GS018793	MUNAL*2/WESTONIA//MOKUE #1	A:A	C:T
BW22GS018794	MUNAL*2/WESTONIA//KASUKO	A:A	C:C
BW22GS018795	MUNAL*2/CHONTE//KASUKO	A:A	T:T
BW22GS018796	MUNAL*2/CHONTE//KASUKO	A:A	T:T
BW22GS018797	KACHU*2/3/ND643//2*PRL/2*PASTOR/4/KASUKO	A:A	T:T
BW22GS018798	BAJ #1*2/5/SW89.5277/BORL95//SKAUZ/3/PRL/2*PASTOR/4/HEILO/6/KASUKO	A:A	T:T
BW22GS018799	KACHU/3/WHEAR//2*PRL/2*PASTOR/4/KASUKO	A:A	T:T
BW22GS018800	KACHU/3/WHEAR//2*PRL/2*PASTOR/4/MOKUE #1	A:A	T:T
BW22GS018801	KACHU*2/3/PBW343*2/KUKUNA//PBW343*2/KUKUNA/4/BORL14*2//KFA/2*KACHU	A:A	T:T
BW22GS018802	SHA7//PRL/VEE#6/3/FASAN/4/HAAS8446/2*FASAN/5/CBRD/KAUZ/6/MILAN/AMSEL/7/FRET2*2/KUKUNA/8/TRAP #1/BOW/3/VEE/PJN//2*TUI/4/BAV92/RAYON/5/KACHU #1/9/COPIO/10/SHORTENED SR26 TRANSLOCATION//2*WBLL1*2/KKTS/3/BECARD	A:A	T:T
BW22GS018803	TACUPETO F2001/BRAMBLING/5/NAC/TH.AC//3*PVN/3/MIRLO/BUC/4/2*PASTOR*2/6/WAXWING/SRTU//WAXWING/KIRITATI/7/MOKUE #1	A:A	T:T
BW22GS018804	PBW343*2/KUKUNA*2//KITE/3/ATTILA*2/PBW65*2//YANAC/4/SAUAL/YANAC//SAUAL/6/WAXWING/KIRITATI*2/3/C80.1/3*BATAVIA//2*WBLL1/4/COPIO/5/ND643//2*ATTILA*2/PASTOR/3/WBLL1*2/KURUKU/4/WBLL1*2/BRAMBLING	A:A	T:T
BW22GS018805	BAVIS #1*2/4/PASTOR//HXL7573/2*BAU/3/SOKOLL/WBLL1/5/BORL14	A:A	C:C
BW22GS018806	NELOKI/4/ATTILA*2/PBW65//PIHA/3/ATTILA/2*PASTOR/8/TACUPETO F2001/6/CNDO/R143//ENTE/MEXI_2/3/AEGILOPS SQUARROSA (TAUS)/4/WEAVER/5/PASTOR/7/ROLF07/9/KASUKO	A:A	T:T
BW22GS018807	PARUS/FRANCOLIN #1/4/MUU #1//PBW343*2/KUKUNA/3/MUU/5/BORL14*2//KFA/2*KACHU	A:A	T:T
BW22GS018808	WHEAR//2*PRL/2*PASTOR/3/WAXBI/4/COPIO/5/NELOKI*2//KACHU/KIRITATI	A:A	T:T
BW22GS018809	WBLL1*2/KKTS//PASTOR/KUKUNA/3/KINGBIRD #1//INQALAB 91*2/TUKURU/5/KAUZ//ALTAR 84/AOS/3/MILAN/KAUZ/4/SAUAL/6/MOKUE #1	A:A	T:T

BW22GS018810	WBLL1*2/KKTS//PASTOR/KUKUNA/3/KINGBIRD #1//INQALAB 91*2/TUKURU/5/KAUZ//ALTAR 84/AOS/3/MILAN/KAUZ/4/SAUAL/6/MOKUE #1	A:A	T:T
BW22GS018811	WBLL1*2/KKTS//PASTOR/KUKUNA/3/KINGBIRD #1//INQALAB 91*2/TUKURU/5/KAUZ//ALTAR 84/AOS/3/MILAN/KAUZ/4/SAUAL/6/MOKUE #1	A:A	T:T
BW22GS018812	WBLL1*2/KKTS//PASTOR/KUKUNA/3/KINGBIRD #1//INQALAB 91*2/TUKURU/5/KAUZ//ALTAR 84/AOS/3/MILAN/KAUZ/4/SAUAL/6/MOKUE #1	A:A	T:T
BW22GS018813	WBLL1*2/KKTS//PASTOR/KUKUNA/3/KINGBIRD #1//INQALAB 91*2/TUKURU/5/KAUZ//ALTAR 84/AOS/3/MILAN/KAUZ/4/SAUAL/6/KASUKO	A:A	T:T
BW22GS018814	WBLL1*2/KKTS//PASTOR/KUKUNA/3/KINGBIRD #1//INQALAB 91*2/TUKURU/5/KAUZ//ALTAR 84/AOS/3/MILAN/KAUZ/4/SAUAL/6/BORL14*2//KFA/2*KACHU	A:A	T:T
BW22GS018815	WBLL1*2/KKTS//PASTOR/KUKUNA/3/KINGBIRD #1//INQALAB 91*2/TUKURU/5/KAUZ//ALTAR 84/AOS/3/MILAN/KAUZ/4/SAUAL/6/BORL14*2//KFA/2*KACHU	A:A	T:T
BW22GS018816	WBLL1*2/KKTS//PASTOR/KUKUNA/3/KINGBIRD #1//INQALAB 91*2/TUKURU/5/KAUZ//ALTAR 84/AOS/3/MILAN/KAUZ/4/SAUAL/6/KUTZ//KFA/2*KACHU	A:A	T:T
BW22GS018817	AMUR*2/CIRO16//KASUKO	A:A	T:T
BW22GS018818	FRET2/KUKUNA//FRET2/3/YANAC/4/FRET2/KIRITATI/5/2*UP2338*2/SHAMA/3/MILAN/KAUZ//CHIL/CHUM18/4/UP2338*2/SHAMA/6/KACHU/3/WHEAR//2*PRL/2*PASTOR	A:A	T:T
BW22GS018819	FRET2/KUKUNA//FRET2/3/YANAC/4/FRET2/KIRITATI/5/2*UP2338*2/SHAMA/3/MILAN/KAUZ//CHIL/CHUM18/4/UP2338*2/SHAMA/6/BORL14*2//KFA/2*KACHU	A:A	T:T
BW22GS018820	FRET2/KUKUNA//FRET2/3/YANAC/4/FRET2/KIRITATI/5/2*UP2338*2/SHAMA/3/MILAN/KAUZ//CHIL/CHUM18/4/UP2338*2/SHAMA/6/BORL14*2//KFA/2*KACHU	A:A	T:T
BW22GS018821	FRET2/KUKUNA//FRET2/3/YANAC/4/FRET2/KIRITATI/5/2*UP2338*2/SHAMA/3/MILAN/KAUZ//CHIL/CHUM18/4/UP2338*2/SHAMA/6/BORL14*2//KFA/2*KACHU	A:A	T:T
BW22GS018822	TACUPETO F2001/6/CNDO/R143//ENTE/MEXI_2/3/AEGILOPS SQUARROSA (TAUS)/4/WEAVER/5/PASTOR/7/ROLF07*2/8/SAUAL/YANAC//SAUAL/9/WBLL1*2/KKTS//PASTOR/KUKUNA/3/KINGBIRD #1//INQALAB 91*2/TUKURU/5/KAUZ//ALTAR 84/AOS/3/MILAN/KAUZ/4/SAUAL	A:A	T:T
BW22GS018823	WADER/KASUKO	A:A	T:T
BW22GS018824	WADER/4/KACHU//WBLL1*2/BRAMBLING*2/3/KACHU/KIRITATI	A:A	T:T
BW22GS018825	KFA/5/REH/HARE//2*BCN/3/CROC_1/AE.SQUARROSA (213)//PGO/4/HUITES/6/REH/HARE//2*BCN/3/CROC_1/AE.SQUARROSA (213)//PGO/4/HUITES/7/BOKOTA/8/BOKOTA/9/KFA/2*KACHU//KACHU/KIRITATI	A:A	T:T
BW22GS018826	BECARD/AKURI/4/WBLL1*2/BRAMBLING//JUCHI/3/WBLL1*2/BRAMBLING/5/KASUKO	A:A	T:T
BW22GS018827	KSW/SAUAL//SAUAL/3/2*BORL14	A:A	T:T
BW22GS018828	KSW/SAUAL//SAUAL/3/2*BORL14	A:A	T:T

BW22GS018829	KSW/SAUAL//SAUAL/3/2*BORL14	A:A	T:T
BW22GS018830	KSW/SAUAL//SAUAL/3/2*BORL14	A:A	T:T
BW22GS018831	KUTZ//KFA/2*KACHU/3/NADI	A:A	T:T
BW22GS018832	MOKUE #1/CHIPAK	A:A	T:T
BW22GS018833	MOKUE #1/CHIPAK	A:A	T:T
BW22GS018834	MOKUE #1/CHIPAK	A:A	T:T
BW22GS018835	BECARD/QUAIU #1//BORL14/3/BORL14*2//KFA/2*KACHU	A:A	T:T
BW22GS018836	SITE/MO//PASTOR/3/TILHI/4/MUNAL #1/5/MUNAL/6/MUCUY/7/MOKUE #1	A:A	T:T
BW22GS018837	NADI#1/3/PBW343*2/KUKUNA*2//FRTL/PIFED/4/NADI#2/5/FRANCOLIN #1/3/PBW343*2/KUKUNA*2//YANAC/4/KINGBIRD #1//INQALAB 91*2/TUKURU	A:A	T:T
BW22GS018838	NADI#1/3/PBW343*2/KUKUNA*2//FRTL/PIFED/4/NADI#2/5/FRANCOLIN #1/3/PBW343*2/KUKUNA*2//YANAC/4/KINGBIRD #1//INQALAB 91*2/TUKURU	A:A	T:T
BW22GS018839	NAINA #2/KASUKO	A:A	T:T
BW22GS018840	KACHU #1/3/C80.1/3*BATAVIA//2*WBLL1/4/KACHU/8/TACUPETO F2001/6/CNDO/R143//ENTE/MEXI_2/3/AEGILOPS SQUARROSA (TAUS)/4/WEAVER/5/PASTOR/7/ROLF07/9/KFA/2*KACHU/10/KASUKO	A:A	T:T
BW22GS018841	TACUPETO F2001/6/CNDO/R143//ENTE/MEXI_2/3/AEGILOPS SQUARROSA (TAUS)/4/WEAVER/5/PASTOR/7/ROLF07*2/8/SAUAL/YANAC//SAUAL/9/KASUKO	A:A	T:T
BW22GS018842	BABAX/LR42//BABAX/3/ER2000/5/BABAX/LR39//BABAX*2/4/KABY/BAV92/3/CROC_1/AE.SQUARROSA (224)//OPATA/6/KUTZ//KFA/2*KACHU	A:A	T:T
BW22GS018843	BAJ #1*2/PREMIO//MOKUE #1	A:A	T:T
BW22GS018844	PBW343*2/KUKUNA//PARUS/3/PBW343*2/KUKUNA/4/BAJ #1/AKURI/5/MOKUE #1	A:A	T:T
BW22GS018845	TACUPETO F2001/BRAMBLING//KACHU/8/REH/HARE//2*BCN/3/CROC_1/AE.SQUARROSA (213)//PGO/4/HUITES/5/T.DICOCCON PI94624/AE.SQUARROSA (409)//BCN/6/REH/HARE//2*BCN/3/CROC_1/AE.SQUARROSA (213)//PGO/4/HUITES/7/MUTUS/9/SUP152//WBLL1*2/BRAMBLING*2/3/KSW/SAUAL//SA	A:A	T:T
BW22GS018846	TACUPETO F2001/BRAMBLING//KACHU/8/REH/HARE//2*BCN/3/CROC_1/AE.SQUARROSA (213)//PGO/4/HUITES/5/T.DICOCCON PI94624/AE.SQUARROSA (409)//BCN/6/REH/HARE//2*BCN/3/CROC_1/AE.SQUARROSA (213)//PGO/4/HUITES/7/MUTUS/9/SUP152//WBLL1*2/BRAMBLING*2/3/KSW/SAUAL//SA	A:A	T:T
BW22GS018847	TACUPETO F2001/BRAMBLING//KACHU/8/REH/HARE//2*BCN/3/CROC_1/AE.SQUARROSA (213)//PGO/4/HUITES/5/T.DICOCCON PI94624/AE.SQUARROSA (409)//BCN/6/REH/HARE//2*BCN/3/CROC_1/AE.SQUARROSA (213)//PGO/4/HUITES/7/MUTUS/9/SUP152//WBLL1*2/BRAMBLING*2/3/KSW/SAUAL//SA	A:A	T:T
BW22GS018848	SUP152/BAJ #1/3/KINGBIRD #1//INQALAB 91*2/TUKURU/8/ATTILA/3*BCN//BAV92/3/TILHI/4/SUP152/5/SUP152/6/KFA/2*KACHU/7/ATTILA/3*BCN//BAV92 /3/PASTOR/4/TACUPETO F2001*2/BRAMBLING/5/PAURAQ	A:A	T:T

BW22GS018849	WBLL1*2/BRAMBLING//VORB/FISCAL/3/BECARD/4/ABLEU/5/KASUKO	A:A	T:T
BW22GS018850	BECARD/FRNCLN//BORL14/3/BORL14*2//KFA/2*KACHU	A:A	T:T
BW22GS018851	BORL14*2/FITIS/4/KACHU/3/WHEAR//2*PRL/2*PASTOR	A:A	T:T
BW22GS018852	BORL14*2/3/KBIRD//WBLL1*2/KURUKU/4/KASUKO	A:A	T:T
BW22GS018853	BORL14*2/3/WBLL1*2/TUKURU//CROSBILL #1/4/MOKUE #1	A:A	T:T
BW22GS018854	BORL14*2/3/WBLL1*2/TUKURU//CROSBILL #1/4/MOKUE #1	A:A	T:T
BW22GS018855	BORL14*2/3/WBLL1*2/TUKURU//CROSBILL #1/4/MOKUE #1	A:A	T:T
BW22GS018856	BORL14*2/3/WBLL1*2/TUKURU//CROSBILL #1/4/MOKUE #1	A:A	T:T
BW22GS018857	BORL14*2/3/WBLL1*2/TUKURU//CROSBILL #1/4/KASUKO	A:A	T:T
BW22GS018858	BORL14*2//KFA/2*KACHU/3/KASUKO	A:A	T:T
BW22GS018859	BORL14*2//BECARD/QUAIU #1/3/KASUKO	A:A	T:T
BW22GS018860	WBLL1*2/BRAMBLING//CHYAK*2/3/KINGBIRD #1//INQALAB 91*2/TUKURU/4/BORL14*2//KFA/2*KACHU	A:A	T:T
BW22GS018861	GRACK/CHYAK/6/ROLF07*2/5/FCT/3/GOV/AZ//MUS/4/DOVE/BUC/7/KACHU/3/WHEAR//2*PRL/2*PASTOR	A:A	T:T
BW22GS018862	GRACK/CHYAK/6/ROLF07*2/5/FCT/3/GOV/AZ//MUS/4/DOVE/BUC/7/KASUKO	A:A	T:T
BW22GS018863	SUP152/HUIRIVIS #1//2*BORL14/3/KASUKO	A:A	T:T
BW22GS018864	SUP152/HUIRIVIS #1//2*BORL14/3/KASUKO	A:A	T:T
BW22GS018865	BECARD/AKURI/3/KACHU//WBLL1*2/BRAMBLING/4/MUTUS/AKURI/5/MOKUE #1	A:A	T:T
BW22GS018866	BECARD/AKURI/3/KACHU//WBLL1*2/BRAMBLING/4/MUTUS/AKURI/5/BORL14*2//KFA/2*KACHU	A:A	T:T
BW22GS018867	KSW/SAUAL//SAUAL/3/TRCH/HUIRIVIS #1/5/UP2338*2/SHAMA/3/MILAN/KAUZ//CHIL/CHUM18/4/UP2338*2/SHAMA/6/KASUKO	A:A	T:T
BW22GS018868	KSW/SAUAL//SAUAL/3/TRCH/HUIRIVIS #1/5/UP2338*2/SHAMA/3/MILAN/KAUZ//CHIL/CHUM18/4/UP2338*2/SHAMA/6/KSW/SAUAL//SAUAL/3/TRCH/HUIRIVIS #1/5/UP2338*2/SHAMA/3/MILAN/KAUZ//CHIL/CHUM18/4/UP2338*2/SHAMA	A:A	T:T
BW22GS018869	KSW/SAUAL//SAUAL/3/TRCH/HUIRIVIS #1/5/UP2338*2/SHAMA/3/MILAN/KAUZ//CHIL/CHUM18/4/UP2338*2/SHAMA/6/PRL/2*PASTOR//KACHU	A:A	T:T
BW22GS018870	KSW/SAUAL//SAUAL/3/TRCH/HUIRIVIS #1/5/UP2338*2/SHAMA/3/MILAN/KAUZ//CHIL/CHUM18/4/UP2338*2/SHAMA/6/WBLL1*2/KKTS//PASTOR/KUKUNA/3/KINGBIRD #1//INQALAB 91*2/TUKURU/5/KAUZ//ALTAR 84/AOS/3/MILAN/KAUZ/4/SAUAL	A:A	T:T

BW22GS018871	KSW/SAUAL//SAUAL/3/TRCH/HUIRIVIS #1/5/UP2338*2/SHAMA/3/MILAN/KAUZ//CHIL/CHUM18/4/UP2338*2/SHAMA/6/WBLL1*2/KKTS//PASTOR/KUKUNA/3/KINGBIRD #1//INQALAB 91*2/TUKURU/5/KAUZ//ALTAR 84/AOS/3/MILAN/KAUZ/4/SAUAL	A:A	T:T
BW22GS018872	WBLL1*2/KURUKU//HEILO/3/WBLL1*2/KURUKU/4/SUP152/BAJ #1/5/SUP152/BAJ #1/6/KASUKO	A:A	T:T
BW22GS018873	SUP152/BAJ #1/4/BAJ #1/3/KIRITATI//ATTILA*2/PASTOR/5/SUP152/BAJ #1/6/KSW/SAUAL//SAUAL/3/TRCH/HUIRIVIS #1/5/UP2338*2/SHAMA/3/MILAN/KAUZ//CHIL/CHUM18/4/UP2338*2/SHAMA	A:A	T:T
BW22GS018874	KACHU #1/KIRITATI//KACHU*2/3/GRACK/CHYAK/4/KASUKO	A:A	T:T
BW22GS018875	TUKURU//BAV92/RAYON/3/MUNAL #1/4/2*KFA/2*KACHU/5/BORL14*2//KFA/2*KACHU	A:A	T:T
BW22GS018876	BORL14/MUNAL #1//MOKUE #1	A:A	T:T
BW22GS018877	BORL14/MUNAL #1//MOKUE #1	A:A	T:T
BW22GS018878	KACHU//WBLL1*2/BRAMBLING/3/KACHU/KIRITATI/4/NELOKI*2//KACHU/KIRITATI	A:A	T:T
BW22GS018879	FRANCOLIN #1*2/HAWFINCH #1//2*MUCUY/4/MUTUS*2/KINGBIRD #1/3/KSW/SAUAL//SAUAL	A:A	T:T
BW22GS018880	SUP152/QUAIU #2//BECARD/QUAIU #1/6/KSW/SAUAL//SAUAL/3/TRCH/HUIRIVIS #1/5/UP2338*2/SHAMA/3/MILAN/KAUZ//CHIL/CHUM18/4/UP2338*2/SHAMA	A:A	T:T
BW22GS018881	SUP152//WBLL1*2/BRAMBLING*2/3/KSW/SAUAL//SAUAL/4/KASUKO	A:A	T:T
BW22GS018882	ALD/CEP75630//CEP75234/PT7219/3/BUC/BJY/4/MILAN/5/3*BORL14	A:A	T:T
BW22GS018883	ALD/CEP75630//CEP75234/PT7219/3/BUC/BJY/4/MILAN/5/3*MUCUY	A:A	T:T
BW22GS018884	BORL14/MOKUE #1//KASUKO	A:A	T:T
BW22GS018885	BORL14/MOKUE #1//KASUKO	A:A	T:T
BW22GS018886	AKEPA/KASUKO//KASUKO	A:A	T:T
BW22GS018887	AKEPA/KASUKO//KASUKO	A:A	T:T
BW22GS018888	BOKOTA*2/MOKUE #1	A:A	T:T
BW22GS018889	BOKOTA*2/MOKUE #1	A:A	T:T
BW22GS018890	BOKOTA*2/MOKUE #1	A:A	T:T
BW22GS018891	WHEAR/VIVITSI//WHEAR/3/PANDORA INIA/4/BORL14*2//KFA/2*KACHU/5/BORL14*2//MUNAL #1/FRANCOLIN #1	A:A	T:T
BW22GS018892	BLOUK #1/4/WHEAR/KUKUNA/3/C80.1/3*BATAVIA//2*WBLL1/5/MUNAL #1*2/6/MOKUE #1	A:A	T:T
BW22GS018893	BLOUK #1/4/WHEAR/KUKUNA/3/C80.1/3*BATAVIA//2*WBLL1/5/MUNAL #1/6/KASUKO/7/KASUKO	A:A	T:T
BW22GS018894	SUP152/FRNCLN//KASUKO/3/BORL14*2//MUNAL #1/FRANCOLIN #1	A:A	T:T
BW22GS018895	BAVIS//ATTILA*2/PBW65/3/2*NELOKI*2//KACHU/KIRITATI	A:A	T:T
BW22GS018896	GLADIUS/3/2*KA/NAC//TRCH/4/KUTZ//KFA/2*KACHU/5/KUTZ//KFA/2*KACHU	A:A	T:T

BW22GS018897	MELON//FILIN/MILAN/3/FILIN/4/PRINIA/PASTOR//HUITES/3/MILAN/OTUS//ATTILA/3*BCN/5/MELON//FILIN/MILAN/3/FILIN/6/BORL14*2//KFA/2*KACHU/7/ROLF07*2/DIAMONDBIRD//TRCH/HUIRIVIS #1/3/BORL14	A:A	T:T
BW22GS018898	SOKOLL/3/PASTOR//HXL7573/2*BAU/4/MASSIV/PPR47.89C/5/2*BORL14*2//KFA/2*KACHU	A:A	T:T
BW22GS018899	ROLF07/YANAC//TACUPETO F2001/BRAMBLING/5/KAUZ//ALTAR 84/AOS/3/MILAN/KAUZ/4/SAUAL/6/2*BORL14//KFA/2*KACHU	A:A	T:T
BW22GS018900	PRL/2*PASTOR//KACHU*2/3/BORL14*2//KFA/2*KACHU	A:A	T:T
BW22GS018901	SUP152/CIRO16*2//KASUKO	A:A	T:T
BW22GS018902	BAV92//IRENA/KAUZ/3/HUITES/4/PVN/5/CIRO16/6/2*MOKUE #1	A:A	T:T
BW22GS018903	UP2338*2/SHAMA/3/MILAN/KAUZ//CHIL/CHUM18/4/UP2338*2/SHAMA/5/COPIO*2/6/SHORTENED SR26 TRANSLOCATION//2*WBLL1*2/KKTS/3/BECARD	A:A	T:T
BW22GS018904	UP2338*2/SHAMA/3/MILAN/KAUZ//CHIL/CHUM18/4/UP2338*2/SHAMA/5/COPIO*2/6/SHORTENED SR26 TRANSLOCATION//2*WBLL1*2/KKTS/3/BECARD	A:A	T:T
BW22GS018905	COPIO/5/UP2338*2/SHAMA/3/MILAN/KAUZ//CHIL/CHUM18/4/UP2338*2/SHAMA/6/2*KACHU//WBLL1*2/BRAMBLING/3/KACHU/KIRITATI	A:A	T:T
BW22GS018906	FRANCOLIN #1/3/PBW343*2/KUKUNA*2//YANAC/4/KINGBIRD #1//INQALAB 91*2/TUKURU/5/KASUKO/6/KASUKO	A:A	T:T
BW22GS018907	FRANCOLIN #1/3/PBW343*2/KUKUNA*2//YANAC/4/KINGBIRD #1//INQALAB 91*2/TUKURU/5/BORL14*2//KFA/2*KACHU/6/BORL14*2//MUNAL #1/FRANCOLIN #1	A:A	T:T
BW22GS018908	NELOKI/5/FRET2/KUKUNA//FRET2/3/TNMU/4/FRET2*2/SHAMA/6/KINGBIRD #1//INQALAB 91*2/TUKURU/7/KASUKO/8/KASUKO	A:A	T:T
BW22GS018909	TACUPETO F2001/BRAMBLING/5/NAC/TH.AC//3*PVN/3/MIRLO/BUC/4/2*PASTOR*2/6/WAXWING/SRTU//WAXWING/KIRITATI/7/KUTZ//KFA/2*KACHU/8/ATTILA/3*BCN//BAV92/3/PASTOR/4/TACUPETO F2001*2/BRAMBLING/5/PAURAQ/6/KFA/2*KACHU	A:A	T:T
BW22GS018910	TACUPETO F2001/BRAMBLING/5/NAC/TH.AC//3*PVN/3/MIRLO/BUC/4/2*PASTOR*2/6/WAXWING/SRTU//WAXWING/KIRITATI/7/2*SUP152//WBLL1*2/BRAMBLING*2/3/KSW/SAUAL//SAUAL	NA	T:T
BW22GS018911	TACUPETO F2001/BRAMBLING/5/NAC/TH.AC//3*PVN/3/MIRLO/BUC/4/2*PASTOR*2/6/WAXWING/SRTU//WAXWING/KIRITATI/7/2*SUP152//WBLL1*2/BRAMBLING*2/3/KSW/SAUAL//SAUAL	A:A	T:T
BW22GS018912	SAUAL/MUTUS*2//CIRO16*2/3/MOKUE #1	A:A	T:T
BW22GS018913	SAUAL/MUTUS*2//CIRO16*2/3/MOKUE #1	A:A	T:T
BW22GS018914	SAUAL/MUTUS*2//CIRO16*2/3/MOKUE #1	A:A	T:T
BW22GS018915	SAUAL/MUTUS*2//CIRO16*2/3/MOKUE #1	A:A	T:T
BW22GS018916	SAUAL/MUTUS*2//CIRO16*2/3/MOKUE #1	A:A	T:T

BW22GS018917	QUAIU #2/BAVIS #1//2*KASUKO	A:A	T:T
BW22GS018918	NELOKI/4/ATTILA*2/PBW65//PIHA/3/ATTILA/2*PASTOR/8/TACUPETO F2001/6/CNDO/R143//ENTE/MEXI_2/3/AEGILOPS SQUARROSA (TAUS)/4/WEAVER/5/PASTOR/7/ROLF07/9/2*KASUKO	A:A	T:T
BW22GS018919	NELOKI/4/ATTILA*2/PBW65//PIHA/3/ATTILA/2*PASTOR/8/TACUPETO F2001/6/CNDO/R143//ENTE/MEXI_2/3/AEGILOPS SQUARROSA (TAUS)/4/WEAVER/5/PASTOR/7/ROLF07/9/2*KASUKO	A:A	T:T
BW22GS018920	SHA7/VEE#5//ARIV92/3/PBW343*2/KUKUNA/4/2*VARIS/MISR 2/3/FRET2/KUKUNA//FRET2/5/2*KASUKO	A:A	T:T
BW22GS018921	WBLL1*2/KKTS//PASTOR/KUKUNA/3/KINGBIRD #1//INQALAB 91*2/TUKURU/5/KAUZ//ALTAR 84/AOS/3/MILAN/KAUZ/4/SAUAL*2/6/BORL14*2//KFA/2*KACHU	A:A	T:T
BW22GS018922	BECARD/AKURI*2/3/KINGBIRD #1//INQALAB 91*2/TUKURU*2/4/NELOKI*2//KACHU/KIRITATI	A:A	T:T
BW22GS018923	KFA/5/REH/HARE//2*BCN/3/CROC_1/AE.SQUARROSA (213)//PGO/4/HUITES/6/REH/HARE//2*BCN/3/CROC_1/AE.SQUARROSA (213)//PGO/4/HUITES/7/BOKOTA/8/BOKOTA/9/BORL14*2//KFA/2*KACHU/10/BORL14*2//KFA/2*KACHU	A:A	T:T
BW22GS018924	KFA/5/REH/HARE//2*BCN/3/CROC_1/AE.SQUARROSA (213)//PGO/4/HUITES/6/REH/HARE//2*BCN/3/CROC_1/AE.SQUARROSA (213)//PGO/4/HUITES/7/BOKOTA/8/BOKOTA*2/9/KACHU*2/3/ND643//2*PRL/2*PASTOR	A:A	T:T
BW22GS018925	BECARD/AKURI/4/WBLL1*2/BRAMBLING//JUCHI/3/WBLL1*2/BRAMBLING/5/KASUKO/6/KASUKO	A:A	T:T
BW22GS018926	KASUKO*2/3/PRL/2*PASTOR//KACHU	A:A	T:T
BW22GS018927	KUTZ//KFA/2*KACHU/3/NADI/5/NADI#1/3/PBW343*2/KUKUNA*2//FRTL/PIFED/4/NADI#2	A:A	T:T
BW22GS018928	BECARD/QUAIU #1//BORL14/3/2*BORL14*2//KFA/2*KACHU	A:A	T:T
BW22GS018929	AMUR/3/KINGBIRD #1//INQALAB 91*2/TUKURU/4/AMUR*2/5/BORL14*2//KFA/2*KACHU	A:A	T:T
BW22GS018930	WAXWING/KIRITATI*2/3/C80.1/3*BATAVIA//2*WBLL1/4/COPIO/5/ND643//2*ATTILA*2/PASTOR/3/WBLL1*2/KURUKU/4/WBLL1*2/BRAMBLING/6/BORL14/7/KASUKO	A:A	T:T
BW22GS018931	WAXWING/KIRITATI*2/3/C80.1/3*BATAVIA//2*WBLL1/4/COPIO/5/ND643//2*ATTILA*2/PASTOR/3/WBLL1*2/KURUKU/4/WBLL1*2/BRAMBLING/6/BORL14/7/KASUKO	A:A	T:T
BW22GS018932	WBLL1*2/BRAMBLING//JUCHI/5/KIRITATI/4/2*BAV92//IRENA/KAUZ/3/HUITES/6/WBLL1*2/BRAMBLING//KACHU*2/7/KUTZ//KFA/2*KACHU	A:A	T:T
BW22GS018933	LIVINGSTON/6/2*MTRWA92.161/PRINIA/5/SERI*3//RL6010/4*YR/3/PASTOR/4/BAV92*2/7/KASUKO	A:A	T:T
BW22GS018934	LIVINGSTON/6/2*MTRWA92.161/PRINIA/5/SERI*3//RL6010/4*YR/3/PASTOR/4/BAV92*2/7/SUP152//WBLL1*2/BRAMBLING*2/3/KSW/SAUAL//SAUAL	A:A	T:T

BW22GS018935	C80.1/3*BATAVIA//2*WBLL1/5/REH/HARE//2*BCN/3/CROC_1/AE.SQUARROSA (213)//PGO/4/HUITES/6/PBW343*2/KUKUNA*2//FRTL/PIFED/7/C80.1/3*BATAVIA//2*WBLL1/5/REH/HARE//2*BCN/3/CROC_1/AE.SQUARROSA (213)//PGO/4/HUITES/8/KASUKO/9/BORL14*2//MUNAL #1/FRANCOLIN #1	A:A	T:T
BW22GS018936	TACUPETO F2001/6/CNDO/R143//ENTE/MEXI_2/3/AEGILOPS SQUARROSA (TAUS)/4/WEAVER/5/PASTOR/7/ROLF07*2/8/SAUAL/YANAC//SAUAL*2/9/KASUKO	A:A	T:T
BW22GS018937	TACUPETO F2001/BRAMBLING//KACHU/8/REH/HARE//2*BCN/3/CROC_1/AE.SQUARROSA (213)//PGO/4/HUITES/5/T.DICOCCON PI94624/AE.SQUARROSA (409)//BCN/6/REH/HARE//2*BCN/3/CROC_1/AE.SQUARROSA (213)//PGO/4/HUITES/7/MUTUS*2/9/BORL14*2//KFA/2*KACHU	A:A	T:T
BW22GS018938	TACUPETO F2001/BRAMBLING//KACHU/8/REH/HARE//2*BCN/3/CROC_1/AE.SQUARROSA (213)//PGO/4/HUITES/5/T.DICOCCON PI94624/AE.SQUARROSA (409)//BCN/6/REH/HARE//2*BCN/3/CROC_1/AE.SQUARROSA (213)//PGO/4/HUITES/7/MUTUS*2/9/BORL14*2//KFA/2*KACHU	A:A	T:T
BW22GS018939	TACUPETO F2001/BRAMBLING//KACHU/8/REH/HARE//2*BCN/3/CROC_1/AE.SQUARROSA (213)//PGO/4/HUITES/5/T.DICOCCON PI94624/AE.SQUARROSA (409)//BCN/6/REH/HARE//2*BCN/3/CROC_1/AE.SQUARROSA (213)//PGO/4/HUITES/7/MUTUS*2/9/BORL14*2//KFA/2*KACHU	A:A	T:T
BW22GS018940	TACUPETO F2001/BRAMBLING//KACHU/8/REH/HARE//2*BCN/3/CROC_1/AE.SQUARROSA (213)//PGO/4/HUITES/5/T.DICOCCON PI94624/AE.SQUARROSA (409)//BCN/6/REH/HARE//2*BCN/3/CROC_1/AE.SQUARROSA (213)//PGO/4/HUITES/7/MUTUS*2/9/BORL14*2//KFA/2*KACHU	A:A	T:T
BW22GS018941	TACUPETO F2001/BRAMBLING//KACHU/8/REH/HARE//2*BCN/3/CROC_1/AE.SQUARROSA (213)//PGO/4/HUITES/5/T.DICOCCON PI94624/AE.SQUARROSA (409)//BCN/6/REH/HARE//2*BCN/3/CROC_1/AE.SQUARROSA (213)//PGO/4/HUITES/7/MUTUS*2/9/SUP152//WBLL1*2/BRAMBLING*2/3/KSW/SAUAL//	A:A	T:T
BW22GS018942	PBW343*2/KUKUNA*2//FRTL/PIFED/3/ABLEU*2/4/WBLL1*2/KUKUNA//KIRITATI/2*TRCH/3/BAJ #1/AKURI	A:A	T:T
BW22GS018943	FRANCOLIN #1//WBLL1*2/KURUKU/3/WBLL1*2/BRAMBLING//CHYAK*2/4/SUP152//WBLL1*2/BRAMBLING*2/3/KSW/SAUAL//SAUAL	A:A	T:T
BW22GS018944	BORL14*2/MUNAL #1/4/SHORTENED SR26 TRANSLOCATION//2*WBLL1*2/KKTS/3/BECARD/5/BORL14*2/MUNAL #1	A:A	T:T
BW22GS018945	CIANO M2018/4/KACHU*2/3/ND643//2*PRL/2*PASTOR/5/BORL14*2//KFA/2*KACHU	A:A	T:T
BW22GS018946	BLOUK #1/MUNAL/3/WBLL1*2/SHAMA//BAJ #1/4/SUP152/BAJ #1/5/KASUKO/6/KASUKO	A:A	T:T
BW22GS018947	PRL/2*PASTOR*2//SKAUZ/BAV92/3/2*BECARD//ND643/2*WBLL1*2/4/KUTZ*2//KFA/2*KACHU	A:A	T:T

BW22GS018948	SUP152/BLOUK #1/3/PRL/2*PASTOR*2//VORB/4/SUP152/BLOUK #1*2/5/BORL14*2//KFA/2*KACHU	A:A	C:T
BW22GS018949	BECARD/AKURI/3/KACHU//WBLL1*2/BRAMBLING/4/MUTUS/AKURI*2/5/BORL14*2//KFA/2*KACHU	A:A	T:T
BW22GS018950	BECARD/AKURI/4/WBLL1*2/BRAMBLING//JUCHI/3/WBLL1*2/BRAMBLING/5/BOKOTA/6/KASUKO/7/KASUKO	A:A	T:T
BW22GS018951	KACHU/BECARD//WBLL1*2/BRAMBLING/4/FRET2/TUKURU//FRET2/3/MUNAL #1*2/6/KSW/SAUAL//SAUAL/3/TRCH/HUIRIVIS #1/5/UP2338*2/SHAMA/3/MILAN/KAUZ//CHIL/CHUM18/4/UP2338*2/SHAMA	A:A	T:T
BW22GS018952	KACHU/BECARD//WBLL1*2/BRAMBLING/4/FRET2/TUKURU//FRET2/3/MUNAL #1*2/6/KSW/SAUAL//SAUAL/3/TRCH/HUIRIVIS #1/5/UP2338*2/SHAMA/3/MILAN/KAUZ//CHIL/CHUM18/4/UP2338*2/SHAMA	A:A	T:T
BW22GS018953	ONIX/KBIRD//BORL14/3/ONIX/KBIRD/4/KUTZ//KFA/2*KACHU/5/KUTZ//KFA/2*KACHU	A:A	T:T
BW22GS018954	KACHU #1/3/T.DICOCCON PI94624/AE.SQUARROSA (409)//BCN/4/2*KACHU/5/MUTUS*2/TECUE #1/6/MUTUS*2/TECUE #1*2/7/MOKUE #1	A:A	T:T
BW22GS018955	MOKUE*2/7/KACHU #1/3/T.DICOCCON PI94624/AE.SQUARROSA (409)//BCN/4/2*KACHU/5/MUTUS*2/TECUE #1/6/MUTUS*2/TECUE #1	A:A	T:T
BW22GS018956	SUP152/QUAIU #2//BECARD/QUAIU #1/3/KUTZ*2//KFA/2*KACHU/4/KUTZ//KFA/2*KACHU	A:A	T:T
BW22GS018957	SUP152/QUAIU #2//BECARD/QUAIU #1/7/ATTILA/3*BCN//BAV92/3/PASTOR/4/TACUPETO F2001*2/BRAMBLING/5/PAURAQ/6/KFA/2*KACHU/8/KFA/2*KACHU*2//MISR 1	A:A	T:T
BW22GS018958	SUP152//WBLL1*2/BRAMBLING*2/3/KSW/SAUAL//SAUAL/4/BORL14*2//KFA/2*KACHU/5/BORL14*2//KFA/2*KAC HU	A:A	T:T
BW22GS018959	SUP152//WBLL1*2/BRAMBLING*2/3/KSW/SAUAL//SAUAL*2/8/ATTILA/3*BCN//BAV92/3/TILHI/4/SUP152/5/SUP1 52/6/KFA/2*KACHU/7/ATTILA/3*BCN//BAV92/3/PASTOR/4/TACUPETO F2001*2/BRAMBLING/5/PAURAQ	A:A	T:T
BW22GS018960	BECARD//ND643/2*WBLL1*2/3/KSW/SAUAL//SAUAL*2/4/KFA/2*KACHU*2//SUP152	A:A	T:T
BW22GS018961	MACE*2/3/KFA/2*KACHU*2//SUP152	A:A	T:T
BW22GS018962	SI-K56/9/BECARD #1/8/BOW/VEE/5/ND/VG9144//KAL/BB/3/YACO/4/CHIL/6/CASKOR/3/CROC_1/AE.SQUARROSA (224)//OPATA/7/PASTOR//MILAN/KAUZ/3/BAV92/10/ATTILA/3*BCN//BAV92/3/TILHI/4/SUP152/5/SUP152/6/KFA /2*KACHU/7/ATTILA/3*BCN//BAV92/3/PASTOR/4/TACUPETO F2001*2/B	A:A	T:T
BW22GS018963	BORL14*2//BECARD/QUAIU #1/3/MOKUE #1	A:A	T:T
BW22GS018964	KAKURU #1	A:A	T:T
BW22GS019306	BONSU	A:A	C:C
BW22GS019307	BECARD//ND643/2*WBLL1*2/3/BORL14	A:A	T:T

BW22GS019308	BORL14*2/BLANCA GRANDE 515	A:A	T:T
BW22GS019309	MUNAL #1/CHIPAK//KASUKO	A:A	T:T
BW22GS019310	KAKURU/KASUKO//KASUKO	A:A	T:T
BW22GS019311	KACHU*2/3/ND643//2*PRL/2*PASTOR*2/4/PRL/2*PASTOR*2//VORB	A:A	T:T
BW22GS019312	KACHU*2/3/ND643//2*PRL/2*PASTOR*2/4/PRL/2*PASTOR*2//VORB	A:A	T:T
BW22GS019313	PBW343*2/KUKUNA//PBW343*2/KUKUNA/3/WBLL1*2/SHAMA//KACHU/4/KASUKO/5/KASUKO	A:A	T:T
BW22GS019314	SAUAL*2/6/CNDO/R143//ENTE/MEXI_2/3/AEGILOPS SQUARROSA (TAUS)/4/WEAVER/5/2*PASTOR/7/PBW343*2/KUKUNA*2//FRTL/PIFED/8/BORL14/9/KASUKO	A:A	T:T
BW22GS019315	SAUAL*2/6/CNDO/R143//ENTE/MEXI_2/3/AEGILOPS SQUARROSA (TAUS)/4/WEAVER/5/2*PASTOR/7/PBW343*2/KUKUNA*2//FRTL/PIFED/8/BORL14/9/KASUKO	A:A	T:T
BW22GS019316	FRANCOLIN #1/3/PBW343*2/KUKUNA*2//YANAC/4/KINGBIRD #1//INQALAB 91*2/TUKURU*2/5/PRL/2*PASTOR*2//VORB	A:A	T:T
BW22GS019317	SAUAL/MUTUS/4/KACHU #1//WBLL1*2/KUKUNA/3/BRBT1*2/KIRITATI/5/2*KACHU/SAUAL*2//COPIO	A:A	T:T
BW22GS019318	SAUAL/MUTUS/4/KACHU #1//WBLL1*2/KUKUNA/3/BRBT1*2/KIRITATI/5/2*KACHU/SAUAL*2//COPIO	A:A	T:T
BW22GS019319	KASUKO/NADI#2//KASUKO	A:A	NA
BW22GS019320	KASUKO/NADI#2//KASUKO	A:A	T:T
BW22GS019321	KASUKO*2/5/MUTUS/DANPHE #1/4/C80.1/3*BATAVIA//2*WBLL1/3/C80.1/3*QT4522//2*PASTOR	A:A	T:T
BW22GS019322	KASUKO/4/CIRO16*2/3/MUU #1/SAUAL//MUU/5/KASUKO	A:A	T:T
BW22GS019323	KASUKO/4/CIRO16*2/3/MUU #1/SAUAL//MUU/5/KASUKO	A:A	T:T
BW22GS019324	BORL14/KASUKO	A:A	T:T
BW22GS019325	CHIPAK/4/SUP152//WBLL1*2/BRAMBLING*2/3/KSW/SAUAL//SAUAL	A:A	T:T
BW22GS019326	KACHU//KIRITATI/2*TRCH/3/KASUKO	A:A	T:T
BW22GS019327	KACHU//KIRITATI/2*TRCH/3/KASUKO	A:A	T:T
BW22GS019328	BABAX/LR42//BABAX*2/3/SHAMA/4/WAXWING*2/KRONSTAD F2004/5/KASUKO	A:A	T:T
BW22GS019329	KACHU*2/3/ND643//2*PRL/2*PASTOR/4/KASUKO	A:A	T:T
BW22GS019330	PRL/2*PASTOR//KACHU/4/KACHU*2/3/ND643//2*PRL/2*PASTOR	A:A	T:T
BW22GS019331	PRL/2*PASTOR//KACHU/3/KASUKO	A:A	T:T
BW22GS019332	PRL/2*PASTOR//KACHU/3/KASUKO	A:A	T:T
BW22GS019333	SUP152/CIRO16//KASUKO	A:A	T:T
BW22GS019334	NELOKI/5/FRET2/KUKUNA//FRET2/3/TNMU/4/FRET2*2/SHAMA/6/KINGBIRD #1//INQALAB 91*2/TUKURU/7/KASUKO	A:A	T:T

BW22GS019335	PARUS/FRANCOLIN #1/4/MUU #1//PBW343*2/KUKUNA/3/MUU/5/KASUKO	A:A	T:T
BW22GS019336	BECARD/AKURI*2/3/PBW343*2/KUKUNA*2//FRTL/PIFED/4/SUP152//WBLL1*2/BRAMBLING*2/3/KSW/SAUAL//SAUAL	A:A	T:T
BW22GS019337	BECARD/AKURI*2/3/PBW343*2/KUKUNA*2//FRTL/PIFED/4/SUP152//WBLL1*2/BRAMBLING*2/3/KSW/SAUAL//SAUAL	A:A	T:T
BW22GS019338	CTRIGO/5/KAUZ//ALTAR 84/AOS/3/KAUZ/4/SW94.15464/6/2*UP2338*2/SHAMA/3/MILAN/KAUZ//CHIL/CHUM18/4/UP2338*2/SHAMA/7/KASUKO	A:A	T:T
BW22GS019339	UP2338*2/SHAMA/3/MILAN/KAUZ//CHIL/CHUM18/4/UP2338*2/SHAMA/5/UP2338*2/VIVITSI/3/FRET2/TUKURU//FRET2/4/MISR 1/6/KASUKO	A:A	T:T
BW22GS019340	KACHU/KIRITATI//BORL14/3/KASUKO	A:A	T:T
BW22GS019341	TC870344/GUI//TEMPORALERA M 87/AGR/3/2*WBLL1/8/BOW/VEE/5/ND/VG9144//KAL/BB/3/YACO/4/CHIL/6/CASKOR/3/CROC_1/AE.SQUARROSA (224)//OPATA/7/PASTOR//MILAN/KAUZ/3/BAV92/9/KASUKO	A:A	T:T
BW22GS019342	TC870344/GUI//TEMPORALERA M 87/AGR/3/2*WBLL1/8/BOW/VEE/5/ND/VG9144//KAL/BB/3/YACO/4/CHIL/6/CASKOR/3/CROC_1/AE.SQUARROSA (224)//OPATA/7/PASTOR//MILAN/KAUZ/3/BAV92/9/KASUKO	A:A	T:T
BW22GS019343	KFA/2*KACHU/4/WBLL1*2/KURUKU//KRONSTAD F2004/3/WBLL1*2/BRAMBLING/5/KASUKO	A:A	T:T
BW22GS019344	KFA/2*KACHU/4/WBLL1*2/KURUKU//KRONSTAD F2004/3/WBLL1*2/BRAMBLING/5/SUP152//WBLL1*2/BRAMBLING*2/3/KSW/SAUAL//SAUAL	A:A	T:T
BW22GS019345	KFA/2*KACHU/4/WBLL1*2/KURUKU//KRONSTAD F2004/3/WBLL1*2/BRAMBLING/5/KUTZ//KFA/2*KACHU	A:A	T:T
BW22GS019346	TACUPETO F2001/BRAMBLING//KIRITATI/3/FRANCOLIN #1/BLOUK #1/4/FRANCOLIN #1/BLOUK #1/5/SHORTENED SR26 TRANSLOCATION//2*WBLL1*2/KKTS/3/BECARD	A:A	T:T
BW22GS019347	TACUPETO F2001/6/CNDO/R143//ENTE/MEXI_2/3/AEGILOPS SQUARROSA (TAUS)/4/WEAVER/5/PASTOR/7/ROLF07*2/8/SAUAL/YANAC//SAUAL/9/KASUKO	A:A	T:T
BW22GS019348	TACUPETO F2001/6/CNDO/R143//ENTE/MEXI_2/3/AEGILOPS SQUARROSA (TAUS)/4/WEAVER/5/PASTOR/7/ROLF07*2/8/SAUAL/YANAC//SAUAL/9/KASUKO	A:A	T:T
BW22GS019349	TACUPETO F2001/6/CNDO/R143//ENTE/MEXI_2/3/AEGILOPS SQUARROSA (TAUS)/4/WEAVER/5/PASTOR/7/ROLF07*2/8/SAUAL/YANAC//SAUAL/9/SUP152//WBLL1*2/BRAMBLING*2/3/KSW/SAUAL//SAUAL	A:A	T:T
BW22GS019350	TACUPETO F2001/6/CNDO/R143//ENTE/MEXI_2/3/AEGILOPS SQUARROSA (TAUS)/4/WEAVER/5/PASTOR/7/ROLF07*2/8/SAUAL/YANAC//SAUAL/9/SUP152//WBLL1*2/BRAMBLING*2/3/KSW/SAUAL//SAUAL	A:A	T:T

BW22GS019351	TACUPETO F2001/6/CNDO/R143//ENTE/MEXI_2/3/AEGILOPS SQUARROSA (TAUS)/4/WEAVER/5/PASTOR/7/ROLF07*2/8/SAUAL/YANAC//SAUAL/9/SUP152//WBLL1*2/BRAMBLING*2/3/KSW/SAUAL//SAUAL	A:A	T:T
BW22GS019352	SAUAL/MUTUS/4/KACHU #1//WBLL1*2/KUKUNA/3/BRBT1*2/KIRITATI/5/BORL14//KFA/2*KACHU	A:A	T:T
BW22GS019353	SAUAL/MUTUS/4/KACHU #1//WBLL1*2/KUKUNA/3/BRBT1*2/KIRITATI/5/KUTZ//KFA/2*KACHU	A:A	T:T
BW22GS019354	SAUAL/MUTUS/4/KACHU #1//WBLL1*2/KUKUNA/3/BRBT1*2/KIRITATI/5/KUTZ//KFA/2*KACHU	A:A	T:T
BW22GS019355	SAUAL/MUTUS/4/KACHU #1//WBLL1*2/KUKUNA/3/BRBT1*2/KIRITATI/5/KUTZ//KFA/2*KACHU	A:A	T:T
BW22GS019356	KASUKO/MOKUE #1	A:A	T:T
BW22GS019357	ND643//2*ATTILA*2/PASTOR/3/WBLL1*2/KURUKU/4/WBLL1*2/BRAMBLING/6/BABAX/LR42//BABAX*2/3/KUKUNA/4/CROSBILL #1/5/BECARD/7/KASUKO	A:A	T:T
BW22GS019358	ND643//2*ATTILA*2/PASTOR/3/WBLL1*2/KURUKU/4/WBLL1*2/BRAMBLING/6/BABAX/LR42//BABAX*2/3/KUKUNA/4/CROSBILL #1/5/BECARD/7/KASUKO	A:A	T:T
BW22GS019359	WBLL1*2/KUKUNA//KIRITATI/3/WBLL1*2/KUKUNA/4/KINGBIRD #1//INQALAB 91*2/TUKURU/5/WBLL1*2/BRAMBLING//KACHU/6/KASUKO	A:A	T:T
BW22GS019360	WBLL1*2/BRAMBLING//JUCHI/3/KINGBIRD #1//INQALAB 91*2/TUKURU/4/WBLL1*2/BRAMBLING//KACHU/5/KASUKO	A:A	T:T
BW22GS019361	FRANCOLIN #1//WBLL1*2/KURUKU/3/WBLL1*2/BRAMBLING//CHYAK/4/SUP152//WBLL1*2/BRAMBLING*2/3/KSW/SAUAL//SAUAL	A:A	C:C
BW22GS019362	FRANCOLIN #1//WBLL1*2/KURUKU/3/WBLL1*2/BRAMBLING//CHYAK/4/SUP152//WBLL1*2/BRAMBLING*2/3/KSW/SAUAL//SAUAL	A:A	C:C
BW22GS019363	SUP152/BAJ #1//KFA/2*KACHU/10/KACHU #1/3/C80.1/3*BATAVIA//2*WBLL1/4/KACHU/8/TACUPETO F2001/6/CNDO/R143//ENTE/MEXI_2/3/AEGILOPS SQUARROSA (TAUS)/4/WEAVER/5/PASTOR/7/ROLF07/9/KFA/2*KACHU	A:A	T:T
BW22GS019364	SUP152/BAJ #1//KFA/2*KACHU/10/KACHU #1/3/C80.1/3*BATAVIA//2*WBLL1/4/KACHU/8/TACUPETO F2001/6/CNDO/R143//ENTE/MEXI_2/3/AEGILOPS SQUARROSA (TAUS)/4/WEAVER/5/PASTOR/7/ROLF07/9/KFA/2*KACHU	A:A	T:T

BW22GS019365	SUP152/BAJ #1//KFA/2*KACHU/10/KACHU #1/3/C80.1/3*BATAVIA//2*WBLL1/4/KACHU/8/TACUPETO F2001/6/CNDO/R143//ENTE/MEXI_2/3/AEGILOPS SQUARROSA (TAUS)/4/WEAVER/5/PASTOR/7/ROLF07/9/KFA/2*KACHU	A:A	T:T
BW22GS019366	KACHU/BECARD//WBLL1*2/BRAMBLING/3/KACHU/KINDE/4/KASUKO	A:A	T:T
BW22GS019367	BORL14*2/FITIS//KASUKO	A:A	T:T
BW22GS019368	BORL14*2/FITIS//KASUKO	A:A	T:T
BW22GS019369	GRACK/CHYAK/6/ROLF07*2/5/FCT/3/GOV/AZ//MUS/4/DOVE/BUC/7/SUP152//WBLL1*2/BRAMBLING*2/3/KSW/SAUAL//SAUAL	A:A	T:T
BW22GS019370	MUTUS//WBLL1*2/BRAMBLING/3/WBLL1*2/BRAMBLING/4/KFA/2*KACHU/5/KASUKO	A:A	T:T
BW22GS019371	KACHU #1/KIRITATI//KACHU*2/3/GRACK/CHYAK/4/KASUKO	A:A	T:T
BW22GS019372	KUTZ//KFA/2*KACHU/3/KASUKO	A:A	T:T
BW22GS019373	KUTZ//KFA/2*KACHU/4/SUP152//WBLL1*2/BRAMBLING*2/3/KSW/SAUAL//SAUAL	A:A	T:T
BW22GS019374	MUNAL/WESTONIA//SUP152/BAJ #1/3/MOKUE #1	A:A	T:T
BW22GS019375	MUNAL/WESTONIA//SUP152/BAJ #1/4/SUP152//WBLL1*2/BRAMBLING*2/3/KSW/SAUAL//SAUAL	A:A	T:T
BW22GS019376	MUTUS//WBLL1*2/BRAMBLING/3/WBLL1*2/BRAMBLING/4/CHYAK1/GRACK/5/KASUKO	A:A	T:T
BW22GS019377	MUTUS//WBLL1*2/BRAMBLING/3/WBLL1*2/BRAMBLING*2/4/KACHU/KIRITATI/5/KUTZ//KFA/2*KACHU	A:A	T:T
BW22GS019378	ROLF07/HUIRIVIS #1//TACUPETO F2001*2/KIRITATI/3/TRCH/HUIRIVIS #1/4/BORL14/5/MOKUE #1	A:A	T:T
BW22GS019379	Pavon 76	A:A	T:T
BW22GS019380	Pavon 76	A:A	T:T
BW22GS019381	KACHU//KIRITATI/2*TRCH/3/KASUKO/4/KASUKO	A:A	T:T
BW22GS019382	BAVIS/NAVJ07/4/2*SUP152//WBLL1*2/BRAMBLING*2/3/KSW/SAUAL//SAUAL	A:A	T:T
BW22GS019383	BONSU/4/2*SUP152//WBLL1*2/BRAMBLING*2/3/KSW/SAUAL//SAUAL	A:A	T:T
BW22GS019384	MELON//FILIN/MILAN/3/FILIN/4/TRCH/SRTU//KACHU/5/KASUKO/6/KASUKO	A:A	T:T
BW22GS019385	TILHI/SOKOLL*2//KINGBIRD #1/7/KFA/2*KACHU/5/WBLL1*2/4/BABAX/LR42//BABAX/3/BABAX/LR42//BABAX/6/KFA/2*KACHU/8/BORL14*2//MUNAL #1/FRANCOLIN #1	A:A	T:T
BW22GS019386	MUTUS//ND643/2*WBLL1*2/3/ONIX/KBIRD*2//KFA/2*KACHU	A:A	T:T
BW22GS019387	BAACH/KASUKO//KASUKO	A:A	T:T
BW22GS019388	FRANCOLIN #1/3/PBW343*2/KUKUNA*2//YANAC/4/KINGBIRD #1//INQALAB 91*2/TUKURU/5/2*SUP152//WBLL1*2/BRAMBLING*2/3/KSW/SAUAL//SAUAL	A:A	T:T
BW22GS019389	TACUPETO F2001/BRAMBLING/5/NAC/TH.AC//3*PVN/3/MIRLO/BUC/4/2*PASTOR*2/6/WAXWING/SRTU//WAXWING/KIRITATI/7/2*KASUKO	A:A	T:T

BW22GS019390	TACUPETO F2001/BRAMBLING/5/NAC/TH.AC//3*PVN/3/MIRLO/BUC/4/2*PASTOR*2/6/WAXWING/SRTU//WAXWING/KIRITATI/7/2*KASUKO	A:A	T:T
BW22GS019391	TACUPETO F2001/BRAMBLING/5/NAC/TH.AC//3*PVN/3/MIRLO/BUC/4/2*PASTOR*2/6/WAXWING/SRTU//WAXWING/KIRITATI/7/2*KASUKO	A:A	T:T
BW22GS019392	KAUZ//ALTAR 84/AOS/3/MILAN/KAUZ/4/SAUAL/5/TRCH/SRTU//KACHU/6/KACHU/SAUAL/7/2*SUP152//WBLL1*2/BRAMBLING*2/3/KSW/SAUAL//SAUAL	A:A	T:T
BW22GS019393	SAUAL/MUTUS//KINGBIRD #1/3/SAUAL/MUTUS/4/2*KASUKO	A:A	T:T
BW22GS019394	NELOKI/4/ATTILA*2/PBW65//PIHA/3/ATTILA/2*PASTOR/8/TACUPETO F2001/6/CNDO/R143//ENTE/MEXI_2/3/AEGILOPS SQUARROSA (TAUS)/4/WEAVER/5/PASTOR/7/ROLF07/9/2*KASUKO	A:A	T:T
BW22GS019395	C80.1/3*BATAVIA//2*WBLL1/5/REH/HARE//2*BCN/3/CROC_1/AE.SQUARROSA (213)//PGO/4/HUITES/6/PBW343*2/KUKUNA*2//FRTL/PIFED/7/C80.1/3*BATAVIA//2*WBLL1/5/REH/HARE//2*BCN/3/CROC_1/AE.SQUARROSA (213)//PGO/4/HUITES/8/2*SUP152//WBLL1*2/BRAMBLING*2/3/KSW/SAUAL//S	A:A	T:T
BW22GS019396	TACUPETO F2001/6/CNDO/R143//ENTE/MEXI_2/3/AEGILOPS SQUARROSA (TAUS)/4/WEAVER/5/PASTOR/7/ROLF07*2/8/SAUAL/YANAC//SAUAL/9/MOKUE #1/10/TACUPETO F2001/6/CNDO/R143//ENTE/MEXI_2/3/AEGILOPS SQUARROSA (TAUS)/4/WEAVER/5/PASTOR/7/ROLF07*2/8/SAUAL/YANAC//SAUAL	A:A	T:T
BW22GS019397	TACUPETO F2001/6/CNDO/R143//ENTE/MEXI_2/3/AEGILOPS SQUARROSA (TAUS)/4/WEAVER/5/PASTOR/7/ROLF07*2/8/SAUAL/YANAC//SAUAL/9/MOKUE #1/10/TACUPETO F2001/6/CNDO/R143//ENTE/MEXI_2/3/AEGILOPS SQUARROSA (TAUS)/4/WEAVER/5/PASTOR/7/ROLF07*2/8/SAUAL/YANAC//SAUAL	A:A	T:T
BW22GS019398	TACUPETO F2001/6/CNDO/R143//ENTE/MEXI_2/3/AEGILOPS SQUARROSA (TAUS)/4/WEAVER/5/PASTOR/7/ROLF07*2/8/SAUAL/YANAC//SAUAL*2/9/KASUKO	A:A	T:T
BW22GS019399	TACUPETO F2001/6/CNDO/R143//ENTE/MEXI_2/3/AEGILOPS SQUARROSA (TAUS)/4/WEAVER/5/PASTOR/7/ROLF07*2/8/SAUAL/YANAC//SAUAL*2/9/KASUKO	A:A	T:T
BW22GS019400	KASUKO*2/MOKUE #1	A:A	T:T
BW22GS019401	KASUKO*2/3/SUP152/QUAIU #2//BECARD/QUAIU #1	A:A	T:T
BW22GS019402	TACUPETO F2001/BRAMBLING//KACHU/8/REH/HARE//2*BCN/3/CROC_1/AE.SQUARROSA (213)//PGO/4/HUITES/5/T.DICOCCON PI94624/AE.SQUARROSA (409)//BCN/6/REH/HARE//2*BCN/3/CROC_1/AE.SQUARROSA (213)//PGO/4/HUITES/7/MUTUS*2/9/BORL14*2//KFA/2*KACHU	A:A	T:T

BW22GS019403	KIRITATI/WBLL1//2*BLOUK #1*2/3/BECARD/QUAIU #1/4/SUP152//WBLL1*2/BRAMBLING*2/3/KSW/SAUAL//SAUAL/5/BECARD//ND643/2*WBLL1/3/KSW/SAUAL//SAUAL	A:A	C:C
BW22GS019404	BLOUK #1/MUNAL/3/WBLL1*2/SHAMA//BAJ #1/4/SUP152/BAJ #1/5/2*SUP152//WBLL1*2/BRAMBLING*2/3/KSW/SAUAL//SAUAL	A:A	T:T
BW22GS019405	BECARD/AKURI/4/WBLL1*2/BRAMBLING//JUCHI/3/WBLL1*2/BRAMBLING/5/BOKOTA/6/KASUKO/7/KASUKO	A:A	T:T
BW22GS019406	KUTZ//KFA/2*KACHU/4/2*SUP152//WBLL1*2/BRAMBLING*2/3/KSW/SAUAL//SAUAL	A:A	T:T
BW22GS019407	SUP152//WBLL1*2/BRAMBLING*2/3/KSW/SAUAL//SAUAL*2/4/NELOKI*2//KACHU/KIRITATI	A:A	T:T
BW22GS019408	SUP152//WBLL1*2/BRAMBLING*2/3/KSW/SAUAL//SAUAL/4/BORL14*2//KFA/2*KACHU/5/BORL14*2//KFA/2*KACHU	A:A	T:T
BW22GS019409	SUP152//WBLL1*2/BRAMBLING*2/3/KSW/SAUAL//SAUAL*2/4/PRL/2*PASTOR//KACHU	A:A	T:T
BW22GS019410	SUP152//WBLL1*2/BRAMBLING*2/3/KSW/SAUAL//SAUAL*2/4/CIRO16/2*BORL14	A:A	T:T
BW22GS019411	SUP152//WBLL1*2/BRAMBLING*2/3/KSW/SAUAL//SAUAL*2/4/CIRO16/2*BORL14	A:A	T:T
BW22GS019412	BECARD//ND643/2*WBLL1*2/3/KSW/SAUAL//SAUAL*2/4/MOKUE #1	A:A	T:T
BW22GS019413	KINDE*2/SOLALA/3/UP2338*2/KKTS*2//YANAC/4/UP2338*2/SHAMA//2*BAJ #1*2/5/FRANCOLIN #1/3/PBW343*2/KUKUNA*2//YANAC/4/KINGBIRD #1//INQALAB 91*2/TUKURU	A:A	T:T
BW22GS019414	T.DICOCCUM ABD/AE.SQUARROSA (895)/7/TRAP#1/BOW/3/VEE/PJN//2*TUI/4/BAV92/RAYON/5/KACHU #1/6/TOBA97/PASTOR/3/T.DICOCCON PI94624/AE.SQUARROSA (409)//BCN/4/BL 1496/MILAN/3/CROC_1/AE.SQUARROSA (205)//KAUZ/9/REH/HARE//2*BCN/3/CROC_1/AE.SQUARROSA (213)//PGO	A:A	T:T
BW22GS019415	EARLY SPELT/AE.SQUARROSA (895)//MANKU/3/MOKUE	A:A	T:T
BW22GS019416	CCB09H083/2*MOKUE #1	A:A	T:T
BW22GS019417	BERGAMO/4/WHEAR/VIVITSI//WHEAR/3/KIRITATI/2*TRCH/5/KACHU//WBLL1*2/BRAMBLING/3/KACHU/KIRITATI	A:A	T:T
BW22GS019418	NADI#2/KASUKO	A:A	T:T
BW22GS019419	KACHU//KIRITATI/2*TRCH/3/KASUKO	A:A	T:T
BW22GS019420	CIRO16/3/TRCH/SRTU//KACHU/4/KASUKO	A:A	T:T
BW22GS019421	PARUS/FRANCOLIN #1/4/MUU #1//PBW343*2/KUKUNA/3/MUU/5/KACHU*2/3/ND643//2*PRL/2*PASTOR	A:A	T:T
BW22GS019422	BABAX/LR42//BABAX/3/ER2000/5/W15.92/4/PASTOR//HXL7573/2*BAU/3/WBLL1/6/SUP152//WBLL1*2/BRAMBLING*2/3/KSW/SAUAL//SAUAL	A:A	T:T
BW22GS019423	SUP152//WBLL1*2/BRAMBLING*2/3/KSW/SAUAL//SAUAL/4/BORL14*2//KFA/2*KACHU	A:A	T:T
BW22GS019424	MACE*2/4/KACHU*2/3/ND643//2*PRL/2*PASTOR	A:A	T:T

BW22GS019425	SOKOLL/3/PASTOR//HXL7573/2*BAU*2/6/OASIS/5*BORL95/5/CNDO/R143//ENTE/MEXI75/3/AE.SQ/4/2*OCI	A:A	C:C
BW22GS019426	NAINA #1	A:A	T:T
BW22GS019427	BAVIS #1//ND643/2*WBLL1/3/BORL14	A:A	T:T
BW22GS019428	AMUR*2/CHIPAK	A:A	NA
BW22GS019429	MUCUY/5/PBW65/2*PASTOR/3/KIRITATI//PBW65/2*SERI.1B/4/DANPHE #1/6/MOKUE #1	A:A	T:T
BW22GS019430	MUCUY/3/KACHU//KIRITATI/2*TRCH/4/MOKUE #1	A:A	T:T
BW22GS019431	MUCUY/3/KACHU//KIRITATI/2*TRCH/4/MOKUE #1	A:A	T:T
BW22GS019432	MUCUY/3/KACHU//KIRITATI/2*TRCH/4/MOKUE #1	NA	T:T
BW22GS019433	KACHU*2/3/ND643//2*PRL/2*PASTOR*2/4/CHIPAK	A:A	T:T
BW22GS019434	MELON//FILIN/MILAN/3/FILIN/4/TRCH/SRTU//KACHU/5/BORL14/6/BORL14//KFA/2*KACHU	A:A	T:T
BW22GS019435	SAUAL/YANAC//SAUAL/5/UP2338*2/SHAMA/3/MILAN/KAUZ//CHIL/CHUM18/4/UP2338*2/SHAMA/6/BORL14/7/ BABAX/LR42//BABAX/3/ER2000/5/W15.92/4/PASTOR//HXL7573/2*BAU/3/WBLL1	A:A	T:T
BW22GS019436	FRANCOLIN #1/3/PBW343*2/KUKUNA*2//YANAC/4/KINGBIRD #1//INQALAB 91*2/TUKURU*2/5/BORL14	A:A	T:T
BW22GS019437	FRANCOLIN #1/3/PBW343*2/KUKUNA*2//YANAC/4/KINGBIRD #1//INQALAB 91*2/TUKURU*2/5/BORL14	A:A	T:T
BW22GS019438	FRANCOLIN #1/3/PBW343*2/KUKUNA*2//YANAC/4/KINGBIRD #1//INQALAB 91*2/TUKURU*2/5/BORL14	A:A	T:T
BW22GS019439	FRANCOLIN #1/3/PBW343*2/KUKUNA*2//YANAC/4/KINGBIRD #1//INQALAB 91*2/TUKURU*2/5/MUNAL #1	A:A	T:T
BW22GS019440	FRANCOLIN #1/3/PBW343*2/KUKUNA*2//YANAC/4/KINGBIRD #1//INQALAB 91*2/TUKURU*2/5/KINGBIRD #1//INQALAB 91*2/TUKURU	A:A	T:T
BW22GS019441	FRANCOLIN #1/3/PBW343*2/KUKUNA*2//YANAC/4/KINGBIRD #1//INQALAB 91*2/TUKURU*2/5/KINGBIRD #1//INQALAB 91*2/TUKURU	A:A	T:T
BW22GS019442	FRANCOLIN #1/3/PBW343*2/KUKUNA*2//YANAC/4/KINGBIRD #1//INQALAB 91*2/TUKURU*2/5/NADI#2	A:A	C:T
BW22GS019443	FRANCOLIN #1/3/PBW343*2/KUKUNA*2//YANAC/4/KINGBIRD #1//INQALAB 91*2/TUKURU*2/5/MUCUY	A:A	T:T
BW22GS019444	FRANCOLIN #1/3/PBW343*2/KUKUNA*2//YANAC/4/KINGBIRD #1//INQALAB 91*2/TUKURU*2/5/BECARD/CHYAK	A:A	T:T
BW22GS019445	KACHU/SAUAL/4/VARIS/MISR 2/3/FRET2/KUKUNA//FRET2/5/KACHU/SAUAL/6/BORL14/7/KASUKO	A:A	T:T
BW22GS019446	PASTOR//HXL7573/2*BAU/3/SOKOLL/WBLL1/6/2*OASIS/5*BORL95/5/CNDO/R143//ENTE/MEXI75/3/AE.SQ/4/2* OCI*2/7/NADI#2	A:A	T:T

BW22GS019447	PASTOR//HXL7573/2*BAU/3/SOKOLL/WBLL1/6/2*OASIS/5*BORL95/5/CNDO/R143//ENTE/MEXI75/3/AE.SQ/4/2*OCI*2/7/NADI#2	A:A	T:T
BW22GS019448	CHIPAK/MOKUE #1	A:A	T:T
BW22GS019449	MUCUY/3/KFA/2*KACHU*2//SUP152	A:A	T:T
BW22GS019450	BLOUK #1/4/WHEAR/KUKUNA/3/C80.1/3*BATAVIA//2*WBLL1/5/MUNAL #1/6/KASUKO	A:A	T:T
BW22GS019451	BLOUK #1/4/WHEAR/KUKUNA/3/C80.1/3*BATAVIA//2*WBLL1/5/MUNAL #1/6/KASUKO	A:A	T:T
BW22GS019452	SUP152/FRNCLN/5/FRANCOLIN #1/3/PBW343*2/KUKUNA*2//YANAC/4/KINGBIRD #1//INQALAB 91*2/TUKURU	A:A	T:T
BW22GS019453	SUP152/FRNCLN//KASUKO	A:A	T:T
BW22GS019454	MUNAL*2/WESTONIA//KASUKO	A:A	T:T
BW22GS019455	MUNAL*2/WESTONIA/3/KUTZ//KFA/2*KACHU	A:A	T:T
BW22GS019456	MUNAL*2/WESTONIA/3/KUTZ//KFA/2*KACHU	A:A	C:C
BW22GS019457	SWSR22T.B./KACHU//2*KACHU/8/ATTILA/3*BCN//BAV92/3/TILHI/4/SUP152/5/SUP152/6/KFA/2*KACHU/7/ATTILA/3*BCN//BAV92/3/PASTOR/4/TACUPETO F2001*2/BRAMBLING/5/PAURAQ	A:A	T:T
BW22GS019458	SHORTENED SR26 TRANSLOCATION//2*WBLL1*2/KKTS/3/BECARD/4/SWSR22T.B.//TACUPETO F2001*2/BRAMBLING/3/2*TACUPETO F2001*2/BRAMBLING	A:A	T:T
BW22GS019459	SHORTENED SR26 TRANSLOCATION//2*WBLL1*2/KKTS/3/BECARD/4/SWSR22T.B.//TACUPETO F2001*2/BRAMBLING/3/2*TACUPETO F2001*2/BRAMBLING	A:A	T:T
BW22GS019460	SHORTENED SR26 TRANSLOCATION//2*WBLL1*2/KKTS/3/BECARD/4/KASUKO	A:A	T:T
BW22GS019461	SWSR22T.B.//TACUPETO F2001*2/BRAMBLING/3/2*TACUPETO F2001*2/BRAMBLING/4/KASUKO	A:A	T:T
BW22GS019462	BAJ #1*2/KISKADEE #1//KASUKO	A:A	T:T
BW22GS019463	BAVIS//ATTILA*2/PBW65/3/KASUKO	A:A	T:T
BW22GS019464	BAVIS//ATTILA*2/PBW65/3/KASUKO	A:A	T:T
BW22GS019465	GLADIUS/3/2*KA/NAC//TRCH/4/KUTZ//KFA/2*KACHU	A:A	T:T
BW22GS019466	GLADIUS/3/2*KA/NAC//TRCH/4/KUTZ//KFA/2*KACHU	A:A	T:T
BW22GS019467	VENDA/4/SUP152//WBLL1*2/BRAMBLING*2/3/KSW/SAUAL//SAUAL	A:A	C:T
BW22GS019468	BECARD//ND643/2*WBLL1/3/SWSR22T.B./2*BLOUK #1//WBLL1*2/KURUKU	A:A	T:T
BW22GS019469	BECARD//ND643/2*WBLL1/3/SWSR22T.B./2*BLOUK #1//WBLL1*2/KURUKU	A:A	T:T
BW22GS019470	KACHU*2/3/ND643//2*PRL/2*PASTOR/4/KASUKO	A:A	T:T
BW22GS019471	KACHU*2/3/ND643//2*PRL/2*PASTOR/4/MOKUE #1	A:A	T:T
BW22GS019472	KACHU*2/3/ND643//2*PRL/2*PASTOR/4/MOKUE #1	A:A	T:T
BW22GS019473	KACHU*2/3/ND643//2*PRL/2*PASTOR/4/MOKUE #1	A:A	T:T
BW22GS019474	KACHU*2/3/ND643//2*PRL/2*PASTOR/4/MOKUE #1	A:A	T:T

BW22GS019475	KACHU*2/3/ND643//2*PRL/2*PASTOR/4/MOKUE #1	A:A	T:T
BW22GS019476	KACHU*2/3/ND643//2*PRL/2*PASTOR/4/MOKUE #1	A:A	T:T
BW22GS019477	KACHU*2/3/ND643//2*PRL/2*PASTOR/4/MOKUE #1	A:A	T:T
BW22GS019478	KACHU*2/3/ND643//2*PRL/2*PASTOR/4/MOKUE #1	A:A	T:T
BW22GS019479	PRL/2*PASTOR//KACHU/4/KACHU*2/3/ND643//2*PRL/2*PASTOR	A:A	T:T
BW22GS019480	PRL/2*PASTOR//KACHU/3/MOKUE #1	A:A	T:T
BW22GS019481	PRL/2*PASTOR//KACHU/3/MOKUE #1	A:A	T:T
BW22GS019482	PRL/2*PASTOR//KACHU/3/MOKUE #1	A:A	T:T
BW22GS019483	PRL/2*PASTOR//KACHU/3/KASUKO	A:A	T:T
BW22GS019484	PRL/2*PASTOR//KACHU/3/BORL14*2//KFA/2*KACHU	A:A	T:T
BW22GS019485	FRANCOLIN #1/BAJ #1//MOKUE #1	A:A	T:T
BW22GS019486	SUP152/CIRO16//KASUKO	A:A	T:T
BW22GS019487	COPIO/5/UP2338*2/SHAMA/3/MILAN/KAUZ//CHIL/CHUM18/4/UP2338*2/SHAMA/6/KSW/SAUAL//SAUAL/3/TRCH/HUIRIVIS #1/5/UP2338*2/SHAMA/3/MILAN/KAUZ//CHIL/CHUM18/4/UP2338*2/SHAMA	A:A	T:T
BW22GS019488	SHA7//PRL/VEE#6/3/FASAN/4/HAAS8446/2*FASAN/5/CBRD/KAUZ/6/MILAN/AMSEL/7/FRET2*2/KUKUNA/8/TRAP #1/BOW/3/VEE/PJN//2*TUI/4/BAV92/RAYON/5/KACHU #1/9/COPIO/10/SHORTENED SR26 TRANSLOCATION//2*WBLL1*2/KKTS/3/BECARD	A:A	T:T
BW22GS019489	WBLL1*2/4/YACO/PBW65/3/KAUZ*2/TRAP//KAUZ/5/KACHU #1*2/6/FRET2/KUKUNA//FRET2/3/TNMU/4/FRET2*2/SHAMA/7/KASUKO	A:A	T:T
BW22GS019490	WBLL1*2/4/YACO/PBW65/3/KAUZ*2/TRAP//KAUZ/5/KACHU #1*2/6/FRET2/KUKUNA//FRET2/3/TNMU/4/FRET2*2/SHAMA/8/CNO79//PF70354/MUS/3/PASTOR/4/BAV92*2/5/HAR311/6/PBW343*2/KUKUNA*2//FRTL/PIFED/7/CNO79//PF70354/MUS/3/PASTOR/4/BAV92*2/5/HAR311	A:A	T:T
BW22GS019491	WBLL1*2/4/YACO/PBW65/3/KAUZ*2/TRAP//KAUZ/5/KACHU #1*2/6/FRET2/KUKUNA//FRET2/3/TNMU/4/FRET2*2/SHAMA/8/CNO79//PF70354/MUS/3/PASTOR/4/BAV92*2/5/HAR311/6/PBW343*2/KUKUNA*2//FRTL/PIFED/7/CNO79//PF70354/MUS/3/PASTOR/4/BAV92*2/5/HAR311	A:A	T:T
BW22GS019492	WBLL1*2/4/YACO/PBW65/3/KAUZ*2/TRAP//KAUZ/5/KACHU #1*2/6/FRET2/KUKUNA//FRET2/3/TNMU/4/FRET2*2/SHAMA/8/CNO79//PF70354/MUS/3/PASTOR/4/BAV92*2/5/HAR311/6/PBW343*2/KUKUNA*2//FRTL/PIFED/7/CNO79//PF70354/MUS/3/PASTOR/4/BAV92*2/5/HAR311	A:A	T:T
BW22GS019493	TACUPETO F2001/BRAMBLING/5/NAC/TH.AC//3*PVN/3/MIRLO/BUC/4/2*PASTOR*2/6/TRCH/SRTU//KACHU/7/PRL/2*PASTOR//KACHU	A:A	T:T

BW22GS019494	TACUPETO F2001/BRAMBLING/5/NAC/TH.AC//3*PVN/3/MIRLO/BUC/4/2*PASTOR*2/6/WAXWING/SRTU//WAXWING/KIRITATI/7/KUTZ//KFA/2*KACHU	A:A	T:T
BW22GS019495	KACHU/SAUAL*2/3/TACUPETO F2001/BRAMBLING//KIRITATI/4/KACHU*2/3/ND643//2*PRL/2*PASTOR	A:A	T:T
BW22GS019496	KACHU/SAUAL*2/3/TACUPETO F2001/BRAMBLING//KIRITATI/4/BORL14*2//KFA/2*KACHU	A:A	T:T
BW22GS019497	KACHU/SAUAL*2/4/ATTILA*2/PBW65//PIHA/3/ATTILA/2*PASTOR/5/BORL14*2//KFA/2*KACHU	A:A	T:T
BW22GS019498	SAUAL/MUTUS//KINGBIRD #1/3/SAUAL/MUTUS/4/KASUKO	A:A	T:T
BW22GS019499	SAUAL/MUTUS//KINGBIRD #1/3/SAUAL/MUTUS/4/KASUKO	A:A	T:T
BW22GS019500	SAUAL/MUTUS//KINGBIRD #1/3/SAUAL/MUTUS/4/BORL14*2//KFA/2*KACHU	A:A	T:T
BW22GS019501	FRET2/KUKUNA//FRET2/3/PARUS/4/FRET2*2/SHAMA*2/5/WBLL1/KUKUNA//TACUPETO F2001/3/UP2338*2/VIVITSI/6/KASUKO	A:A	T:T
BW22GS019502	FRET2/KUKUNA//FRET2/3/PARUS/4/FRET2*2/SHAMA*2/5/WBLL1/KUKUNA//TACUPETO F2001/3/UP2338*2/VIVITSI/6/KASUKO	A:A	T:T
BW22GS019503	FRET2/KUKUNA//FRET2/3/PARUS/4/FRET2*2/SHAMA*2/5/WBLL1/KUKUNA//TACUPETO F2001/3/UP2338*2/VIVITSI/6/KASUKO	A:A	T:T
BW22GS019504	PBW343*2/KUKUNA*2//KITE/3/ATTILA*2/PBW65*2//YANAC/4/SAUAL/YANAC//SAUAL/6/WAXWING/KIRITATI*2/3/C80.1/3*BATAVIA//2*WBLL1/4/COPIO/5/ND643//2*ATTILA*2/PASTOR/3/WBLL1*2/KURUKU/4/WBLL1*2/BRAMBLING	A:A	T:T
BW22GS019505	PARUS/FRANCOLIN #1/4/MUU #1//PBW343*2/KUKUNA/3/MUU/5/KASUKO	A:A	T:T
BW22GS019506	WBLL1*2/KKTS//PASTOR/KUKUNA/3/KINGBIRD #1//INQALAB 91*2/TUKURU/5/KAUZ//ALTAR 84/AOS/3/MILAN/KAUZ/4/SAUAL/6/MOKUE #1	A:A	T:T
BW22GS019507	AMUR*2/CIRO16//KASUKO	A:A	T:T
BW22GS019508	ROLF07/YANAC//TACUPETO F2001/BRAMBLING*2/5/UP2338*2/SHAMA/3/MILAN/KAUZ//CHIL/CHUM18/4/UP2338*2/SHAMA/6/BORL14	A:A	T:T
BW22GS019509	FRET2/KUKUNA//FRET2/3/YANAC/4/FRET2/KIRITATI/5/2*UP2338*2/SHAMA/3/MILAN/KAUZ//CHIL/CHUM18/4/UP2338*2/SHAMA/6/BORL14*2//KFA/2*KACHU	A:A	T:T
BW22GS019510	WBLL1/3/STAR//KAUZ/STAR/4/BAV92/RAYON/5/TRAP#1/BOW/3/VEE/PJN//2*TUI/4/BAV92/RAYON*2/8/TACUPETO F2001/6/CNDO/R143//ENTE/MEXI_2/3/AEGILOPS SQUARROSA (TAUS)/4/WEAVER/5/PASTOR/7/ROLF07/9/SUP152//WBLL1*2/BRAMBLING*2/3/KSW/SAUAL//SAUAL	A:A	T:T
BW22GS019511	BECARD/AKURI*2/3/PBW343*2/KUKUNA*2//FRTL/PIFED/4/SUP152//WBLL1*2/BRAMBLING*2/3/KSW/SAUAL//SAUAL	A:A	T:T
BW22GS019512	TACUPETO F2001/6/CNDO/R143//ENTE/MEXI_2/3/AEGILOPS SQUARROSA (TAUS)/4/WEAVER/5/PASTOR/7/ROLF07*2/8/SAUAL/YANAC//SAUAL/9/KASUKO	A:A	T:T

BW22GS019513	WADER/4/KACHU//WBLL1*2/BRAMBLING*2/3/KACHU/KIRITATI	A:A	T:T
BW22GS019514	WADER/4/KACHU//WBLL1*2/BRAMBLING*2/3/KACHU/KIRITATI	A:A	T:T
BW22GS019515	BECARD/AKURI/4/WBLL1*2/BRAMBLING//JUCHI/3/WBLL1*2/BRAMBLING/5/KUTZ//KFA/2*KACHU	A:A	C:C
BW22GS019516	MOKUE #1/3/BORL14*2//KFA/2*KACHU	A:A	T:T
BW22GS019517	MOKUE #1/3/BORL14*2//KFA/2*KACHU	A:A	T:T
BW22GS019518	MOKUE #1/3/BORL14*2//KFA/2*KACHU	A:A	C:C
BW22GS019519	MOKUE #1/3/KACHU/BECARD//WBLL1*2/BRAMBLING	A:A	C:T
BW22GS019520	SITE/MO//PASTOR/3/TILHI/4/MUNAL #1/5/MUNAL/6/MUCUY/7/MOKUE #1	A:A	T:T
BW22GS019521	QUAIU #1/5/KIRITATI/4/2*BAV92//IRENA/KAUZ/3/HUITES/6/BECARD/QUAIU #1/7/SUP152//WBLL1*2/BRAMBLING*2/3/KSW/SAUAL//SAUAL	A:A	C:T
BW22GS019522	NADI#1/3/PBW343*2/KUKUNA*2//FRTL/PIFED/4/NADI#2/5/FRANCOLIN #1/3/PBW343*2/KUKUNA*2//YANAC/4/KINGBIRD #1//INQALAB 91*2/TUKURU	A:A	T:T
BW22GS019523	NAINA #2/3/PRL/2*PASTOR//KACHU	A:A	T:T
BW22GS019524	KFA/2*KACHU/4/WBLL1*2/KURUKU//KRONSTAD F2004/3/WBLL1*2/BRAMBLING/5/KUTZ//KFA/2*KACHU	A:A	NA
BW22GS019525	TACUPETO F2001/BRAMBLING//KIRITATI/3/FRANCOLIN #1/BLOUK #1/4/FRANCOLIN #1/BLOUK #1/5/SHORTENED SR26 TRANSLOCATION//2*WBLL1*2/KKTS/3/BECARD	A:A	T:T
BW22GS019526	KASUKO/3/SUP152/QUAIU #2//BECARD/QUAIU #1	A:A	T:T
BW22GS019527	KASUKO/3/SUP152/QUAIU #2//BECARD/QUAIU #1	A:A	T:T
BW22GS019528	BOKOTA/5/UP2338*2/VIVITSI/3/FRET2/TUKURU//FRET2/4/MISR 1/6/BABAX/LR42//BABAX*2/3/KUKUNA/4/CROSBILL #1/5/BECARD/7/BORL14//KFA/2*KACHU	A:A	T:T
BW22GS019529	WBLL1*2/BRAMBLING//JUCHI/3/KINGBIRD #1//INQALAB 91*2/TUKURU/4/WBLL1*2/BRAMBLING//KACHU/5/KASUKO	A:A	T:T
BW22GS019530	BABAX/LR42//BABAX/3/ER2000/5/BABAX/LR39//BABAX*2/4/KABY/BAV92/3/CROC_1/AE.SQUARROSA (224)//OPATA/6/SUP152//WBLL1*2/BRAMBLING*2/3/KSW/SAUAL//SAUAL	A:A	T:T
BW22GS019531	TACUPETO F2001/BRAMBLING//KACHU/8/REH/HARE//2*BCN/3/CROC_1/AE.SQUARROSA (213)//PGO/4/HUITES/5/T.DICOCCON PI94624/AE.SQUARROSA (409)//BCN/6/REH/HARE//2*BCN/3/CROC_1/AE.SQUARROSA (213)//PGO/4/HUITES/7/MUTUS/9/BORL14*2//KFA/2*KACHU	A:A	T:T
BW22GS019532	FRANCOLIN #1//WBLL1*2/KURUKU/3/WBLL1*2/BRAMBLING//CHYAK/4/SUP152//WBLL1*2/BRAMBLING*2/3/KSW/SAUAL//SAUAL	A:A	C:C
BW22GS019533	SUP152/BAJ #1/3/KINGBIRD #1//INQALAB 91*2/TUKURU/8/ATTILA/3*BCN//BAV92/3/TILHI/4/SUP152/5/SUP152/6/KFA/2*KACHU/7/ATTILA/3*BCN//BAV92/3/PASTOR/4/TACUPETO F2001*2/BRAMBLING/5/PAURAQ	A:A	T:T

BW22GS019534	SUP152/BAJ #1/3/KINGBIRD #1//INQALAB 91*2/TUKURU/8/ATTILA/3*BCN//BAV92/3/TILHI/4/SUP152/5/SUP152/6/KFA/2*KACHU/7/ATTILA/3*BCN//BAV92 /3/PASTOR/4/TACUPETO F2001*2/BRAMBLING/5/PAURAQ	A:A	T:T
BW22GS019535	KACHU/BECARD//WBLL1*2/BRAMBLING/3/KACHU/KINDE/4/KASUKO	A:A	T:T
BW22GS019536	PREMIO/4/CROC_1/AE.SQUARROSA (205)//KAUZ/3/PIFED/5/VORB/FISCAL//KACHU/3/WBLL1*2/BRAMBLING/6/KASUKO	NA	T:T
BW22GS019537	PREMIO/4/CROC_1/AE.SQUARROSA (205)//KAUZ/3/PIFED/5/VORB/FISCAL//KACHU/3/WBLL1*2/BRAMBLING/6/KASUKO	A:A	T:T
BW22GS019538	PREMIO/4/CROC_1/AE.SQUARROSA (205)//KAUZ/3/PIFED/5/VORB/FISCAL//KACHU/3/WBLL1*2/BRAMBLING/6/KASUKO	A:A	T:T
BW22GS019539	BORL14*2//MUNAL #1/FRANCOLIN #1/4/KACHU*2/3/ND643//2*PRL/2*PASTOR	A:A	T:T
BW22GS019540	KACHU//WBLL1*2/BRAMBLING*2/6/ROLF07*2/5/REH/HARE//2*BCN/3/CROC_1/AE.SQUARROSA (213)//PGO/4/HUITES/7/KUTZ*2//KFA/2*KACHU	A:A	T:T
BW22GS019541	CIANO M2018/4/KACHU//WBLL1*2/BRAMBLING*2/3/KACHU/KIRITATI	A:A	T:T
BW22GS019542	BLOUK #1/MUNAL/3/WBLL1*2/SHAMA//BAJ #1/4/SUP152/BAJ #1/5/MOKUE #1	A:A	T:T
BW22GS019543	BECARD/AKURI/3/KACHU//WBLL1*2/BRAMBLING/4/MUTUS/AKURI/5/MOKUE #1	A:A	T:T
BW22GS019544	BECARD/AKURI/3/KACHU//WBLL1*2/BRAMBLING/4/MUTUS/AKURI/5/MOKUE #1	A:A	T:T
BW22GS019545	BECARD/AKURI/3/KACHU//WBLL1*2/BRAMBLING/4/MUTUS/AKURI/5/MOKUE #1	A:A	T:T
BW22GS019546	ATTILA/3*BCN//BAV92/3/PASTOR/4/MUNAL #1/5/MUNAL/6/2*BECARD/QUAIU #1/7/KFA/2*KACHU*2//SUP152	A:A	T:T
BW22GS019547	SUP152/BAJ #1/3/KACHU//WBLL1*2/BRAMBLING/6/KSW/SAUAL//SAUAL/3/TRCH/HUIRIVIS #1/5/UP2338*2/SHAMA/3/MILAN/KAUZ//CHIL/CHUM18/4/UP2338*2/SHAMA	A:A	T:T
BW22GS019548	CHIPAK*2//KFA/2*KACHU/3/KUTZ//KFA/2*KACHU	A:A	T:T
BW22GS019549	CHIPAK*2//KFA/2*KACHU/3/KUTZ//KFA/2*KACHU	A:A	T:T
BW22GS019550	CHIPAK*2//KFA/2*KACHU/7/ATTILA/3*BCN//BAV92/3/PASTOR/4/TACUPETO F2001*2/BRAMBLING/5/PAURAQ/6/KFA/2*KACHU	A:A	T:T
BW22GS019551	TUKURU//BAV92/RAYON/3/MUNAL #1/4/2*KFA/2*KACHU/5/BORL14*2//KFA/2*KACHU	A:A	T:T
BW22GS019552	KUTZ//KFA/2*KACHU/3/KASUKO	A:A	T:T
BW22GS019553	BORL14*2//KFA/2*KACHU/3/KASUKO	A:A	T:T
BW22GS019554	ROLF07*2/DIAMONDBIRD//TRCH/HUIRIVIS #1/3/BORL14/4/NELOKI*2//KACHU/KIRITATI	A:A	T:T
BW22GS019555	ROLF07*2/DIAMONDBIRD//TRCH/HUIRIVIS #1/3/BORL14/4/NELOKI*2//KACHU/KIRITATI	A:A	T:T
BW22GS019556	FRANCOLIN #1*2/HAWFINCH #1//2*MUCUY/4/MUTUS*2/KINGBIRD #1/3/KSW/SAUAL//SAUAL	A:A	T:T
BW22GS019557	FRANCOLIN #1*2/HAWFINCH #1//2*MUCUY/4/MUTUS*2/KINGBIRD #1/3/KSW/SAUAL//SAUAL	A:A	T:T
BW22GS019558	FRANCOLIN #1*2/HAWFINCH #1//2*MUCUY/4/MUTUS*2/KINGBIRD #1/3/KSW/SAUAL//SAUAL	A:A	T:T

BW22GS019559	TACUPETO F2001/6/CNDO/R143//ENTE/MEXI_2/3/AEGILOPS SQUARROSA (TAUS)/4/WEAVER/5/PASTOR/7/ROLF07/8/PBW343*2/KUKUNA*2//FRTL/PIFED/9/KUTZ*2//KFA/2*KACHU	A:A	T:T
BW22GS019560	ND643/2*TRCH//BECARD/3/BECARD/4/SUP152*2/TECUE #1/5/KUTZ*2//KFA/2*KACHU	A:A	T:T
BW22GS019561	MUNAL/WESTONIA//SUP152/BAJ #1/3/MOKUE #1	A:A	T:T
BW22GS019562	BORL14/MOKUE #1//KASUKO	A:A	C:T
BW22GS019563	MUCUY/4/SUP152//WBLL1*2/BRAMBLING*2/3/KSW/SAUAL//SAUAL/5/MOKUE #1	A:A	T:T
BW22GS019564	QUAIU #1/SUP152/3/BAJ #1/TECUE #1//MUTUS*2/TECUE #1/4/CHIPAK*2/3/KSW/SAUAL//SAUAL	A:A	T:T
BW22GS019565	PRL/2*PASTOR//PBW343*2/KUKUNA/3/ROLF07/4/BERKUT//PBW343*2/KUKUNA/5/KASUKO/6/BORL14//KFA/2*KACHU	A:A	T:T
BW22GS019566	MUTUS//ND643/2*WBLL1/4/SHORTENED SR26 TRANSLOCATION//2*WBLL1*2/KKTS/3/BECARD/5/MOKUE #1	A:A	T:T
BW22GS019567	CHIBIA//PRLII/CM65531/3/MISR 2*2/4/HUW234+LR34/PRINIA//PBW343*2/KUKUNA/3/ROLF07/5/BORL14*2//KFA/2*KACHU/6/BORL14*2//KFA/2*KACHU	A:A	T:T
BW22GS019568	MELON//FILIN/MILAN/3/FILIN/4/TRCH/SRTU//KACHU/5/2*KACHU//WBLL1*2/BRAMBLING/3/KACHU/KIRITATI	A:A	T:T
BW22GS019569	MELON//FILIN/MILAN/3/FILIN/4/TRCH/SRTU//KACHU/5/2*KACHU//WBLL1*2/BRAMBLING/3/KACHU/KIRITATI	A:A	T:T
BW22GS019570	KACHU/3/WHEAR//2*PRL/2*PASTOR/4/KASUKO/5/PRL/2*PASTOR//KACHU	A:A	T:T
BW22GS019571	KACHU/3/WHEAR//2*PRL/2*PASTOR/4/KASUKO/5/PRL/2*PASTOR//KACHU	A:A	T:T
BW22GS019572	BAV92//IRENA/KAUZ/3/HUITES/4/PVN/5/CIRO16/6/2*MOKUE #1	A:A	T:T
BW22GS019573	CIRO16/3/TRCH/SRTU//KACHU/4/KUTZ//KFA/2*KACHU/5/KUTZ//KFA/2*KACHU	A:A	T:T
BW22GS019574	KACHU/3/WHEAR//2*PRL/2*PASTOR/4/BOKOTA*2/5/MOKUE #1	A:A	T:T
BW22GS019575	FRANCOLIN #1/3/PBW343*2/KUKUNA*2//YANAC/4/KINGBIRD #1//INQALAB 91*2/TUKURU*2/5/BORL14	A:A	T:T
BW22GS019576	TACUPETO F2001/BRAMBLING/5/NAC/TH.AC//3*PVN/3/MIRLO/BUC/4/2*PASTOR*2/6/WAXWING/SRTU//WAXWING/KIRITATI/7/KASUKO/8/ROLF07*2/DIAMONDBIRD//TRCH/HUIRIVIS #1/3/BORL14	A:A	T:T
BW22GS019577	SAUAL/MUTUS//KINGBIRD #1/3/SAUAL/MUTUS/4/BORL14*2//KFA/2*KACHU/5/BORL14*2//KFA/2*KACHU	A:A	T:T
BW22GS019578	SAUAL/MUTUS//KINGBIRD #1/3/SAUAL/MUTUS/4/KUTZ//KFA/2*KACHU/5/KUTZ*2//KFA/2*KACHU	A:A	T:T
BW22GS019579	SAUAL/MUTUS*2//CIRO16*2/3/MOKUE #1	A:A	T:T
BW22GS019580	SAUAL/MUTUS*2//CIRO16*2/3/MOKUE #1	A:A	T:T

BW22GS019581	SAUAL/MUTUS*2//CIRO16*2/3/MOKUE #1	A:A	T:T
BW22GS019582	FRET2/KUKUNA//FRET2/3/PARUS/4/FRET2*2/SHAMA*2/5/WBLL1/KUKUNA//TACUPETO F2001/3/UP2338*2/VIVITSI/6/PRL/2*PASTOR//KACHU/7/KFA/2*KACHU*2//SUP152	A:A	T:T
BW22GS019583	CROC_1/AE.SQUARROSA (205)//BORL95/3/PRL/SARA//TSI/VEE#5/4/FRET2/6/MTRWA92.161/PRINIA/5/SERI*3//RL6010/4*YR/3/PASTOR/4/ BAV92/7/BORL14//KFA/2*KACHU/8/BORL14*2//KFA/2*KACHU	A:A	T:T
BW22GS019584	CROC_1/AE.SQUARROSA (205)//BORL95/3/PRL/SARA//TSI/VEE#5/4/FRET2/6/MTRWA92.161/PRINIA/5/SERI*3//RL6010/4*YR/3/PASTOR/4/ BAV92/7/2*KACHU//WBLL1*2/BRAMBLING/3/KACHU/KIRITATI	A:A	T:T
BW22GS019585	QUAIU #2/BAVIS #1//KAKURU/4/SHORTENED SR26 TRANSLOCATION//2*WBLL1*2/KKTS/3/BECARD	A:A	T:T
BW22GS019586	FRET2*2/SHAMA//PARUS/3/FRET2*2/KUKUNA/4/WBLL1/KUKUNA//TACUPETO F2001/3/UP2338*2/VIVITSI/5/PRL/2*PASTOR//KACHU/6/KACHU//WBLL1*2/BRAMBLING*2/3/KACHU/KIRITATI	A:A	T:T
BW22GS019587	FRET2*2/SHAMA//PARUS/3/FRET2*2/KUKUNA/4/WBLL1/KUKUNA//TACUPETO F2001/3/UP2338*2/VIVITSI/5/PRL/2*PASTOR//KACHU/6/KACHU//WBLL1*2/BRAMBLING*2/3/KACHU/KIRITATI	A:A	T:T
BW22GS019588	AMUR*2/CIRO16*2/3/KFA/2*KACHU*2//SUP152	A:A	T:T
BW22GS019589	WADER #2/4/2*SHORTENED SR26 TRANSLOCATION//2*WBLL1*2/KKTS/3/BECARD	A:A	T:T
BW22GS019590	WADER #2/4/2*SHORTENED SR26 TRANSLOCATION//2*WBLL1*2/KKTS/3/BECARD	A:A	T:T
BW22GS019591	KUTZ//KFA/2*KACHU/3/CHIPAK/4/CHIPAK*2/3/KSW/SAUAL//SAUAL	A:A	T:T
BW22GS019592	MOKUE #1/3/BORL14*2//KFA/2*KACHU/4/BORL14*2//KFA/2*KACHU	A:A	T:T
BW22GS019593	MOKUE #1/3/BORL14*2//KFA/2*KACHU/4/BORL14*2//KFA/2*KACHU	A:A	T:T
BW22GS019594	MOKUE #1*2/NADI	A:A	T:T
BW22GS019595	MOKUE #1*2/NADI	A:A	T:T
BW22GS019596	MOKUE #1*2/NADI	A:A	T:T
BW22GS019597	MOKUE #1/6/WBLL1*2/KKTS//PASTOR/KUKUNA/3/KINGBIRD #1//INQALAB 91*2/TUKURU/5/KAUZ//ALTAR 84/AOS/3/MILAN/KAUZ/4/SAUAL/7/BORL14*2//BECARD/QUAIU #1	A:A	T:T
BW22GS019598	NADI#1/3/PBW343*2/KUKUNA*2//FRTL/PIFED/4/NADI#2*2/5/KASUKO	A:A	T:T
BW22GS019599	NAINA #3/3/NELOKI*2//KACHU/KIRITATI/4/NAINA #2	A:A	T:T
BW22GS019600	WAXWING/4/BL 1496/MILAN/3/CROC_1/AE.SQUARROSA (205)//KAUZ/5/FRNCLN/6/KINGBIRD #1//INQALAB 91*2/TUKURU/7/BECARD/QUAIU #1/8/2*BORL14*2//KFA/2*KACHU	A:A	T:T
BW22GS019601	WAXWING/4/BL 1496/MILAN/3/CROC_1/AE.SQUARROSA (205)//KAUZ/5/FRNCLN/6/KINGBIRD #1//INQALAB 91*2/TUKURU/7/BECARD/QUAIU #1/8/2*KACHU//WBLL1*2/BRAMBLING*2/3/KACHU/KIRITATI	A:A	T:T

BW22GS019602	W15.92/4/PASTOR//HXL7573/2*BAU/3/WBLL1/7/CNO79//PF70354/MUS/3/PASTOR/4/BAV92/5/FRET2/KUKUNA//FRET2/6/MILAN/KAUZ//PRINIA/3/BAV92*2/8/MOKUE #1	A:A	T:T
BW22GS019603	KFA/2*KACHU/4/WBLL1*2/KURUKU//KRONSTAD F2004/3/WBLL1*2/BRAMBLING/5/KUTZ//KFA/2*KACHU/6/KUTZ//KFA/2*KACHU	A:A	T:T
BW22GS019604	BAJ #1*2/PREMIO//2*MOKUE #1	A:A	T:T
BW22GS019605	PBW343*2/KUKUNA*2//FRTL/PIFED/3/ABLEU*2/4/MOKUE #1	A:A	T:T
BW22GS019606	PBW343*2/KUKUNA*2//FRTL/PIFED/3/ABLEU*2/4/MOKUE #1	A:A	T:T
BW22GS019607	SUP152/BAJ #1/3/KINGBIRD #1//INQALAB 91*2/TUKURU*2/4/MOKUE #1	A:A	T:T
BW22GS019608	SUP152/BAJ #1/3/KINGBIRD #1//INQALAB 91*2/TUKURU*2/4/MOKUE #1	A:A	T:T
BW22GS019609	SUP152/BAJ #1/3/KINGBIRD #1//INQALAB 91*2/TUKURU*2/4/MOKUE #1	A:A	T:T
BW22GS019610	SUP152/BAJ #1/3/KINGBIRD #1//INQALAB 91*2/TUKURU*2/4/MOKUE #1	A:A	T:T
BW22GS019611	CIRO16/2*BORL14//MOKUE #1/3/BORL14*2//KFA/2*KACHU	A:A	T:T
BW22GS019612	BORL14*2/MUNAL #1*2/3/SWSR22T.B./2*BLOUK #1//WBLL1*2/KURUKU	A:A	T:T
BW22GS019613	BORL14*2/MUNAL #1/4/SHORTENED SR26 TRANSLOCATION//2*WBLL1*2/KKTS/3/BECARD/5/BORL14*2/MUNAL #1	A:A	T:T
BW22GS019614	BORL14*2/3/KBIRD//WBLL1*2/KURUKU/4/SUP152//WBLL1*2/BRAMBLING*2/3/KSW/SAUAL//SAUAL/5/ROLF07*2/DIAMONDBIRD//TRCH/HUIRIVIS #1/3/BORL14	A:A	T:T
BW22GS019615	BORL14*2//KFA/2*KACHU/3/MOKUE #1/4/BORL14*2//KFA/2*KACHU	A:A	T:T
BW22GS019616	BORL14*2//BECARD/QUAIU #1/3/MOKUE #1/4/BORL14*2//KFA/2*KACHU	A:A	T:T
BW22GS019617	CNO79//PF70354/MUS/3/PASTOR/4/BAV92*2/5/HAR311/6/BECARD/QUAIU #1/7/BECARD/QUAIU #1/8/BORL14//KFA/2*KACHU/9/ROLF07*2/DIAMONDBIRD//TRCH/HUIRIVIS #1/3/BORL14	A:A	T:T
BW22GS019618	BAJ #1/AKURI*2//HUIRIVIS #1/KBIRD*2/3/KUTZ*2//KFA/2*KACHU	A:A	T:T
BW22GS019619	NADI#1*2/3/ATTILA*2/PBW65*2//MURGA*2/4/BORL14*2//KFA/2*KACHU	A:A	T:T
BW22GS019620	NADI#1*2/3/ATTILA*2/PBW65*2//MURGA*2/4/BORL14*2//KFA/2*KACHU	A:A	T:T
BW22GS019621	NADI#1*2/3/ATTILA*2/PBW65*2//MURGA*2/4/BORL14*2//KFA/2*KACHU	A:A	T:T
BW22GS019622	KACHU//WBLL1*2/BRAMBLING*2/6/ROLF07*2/5/REH/HARE//2*BCN/3/CROC_1/AE.SQUARROSA (213)//PGO/4/HUITES*2/7/KUTZ//KFA/2*KACHU	A:A	T:T
BW22GS019623	KACHU//WBLL1*2/BRAMBLING*2/6/ROLF07*2/5/REH/HARE//2*BCN/3/CROC_1/AE.SQUARROSA (213)//PGO/4/HUITES*2/7/KUTZ//KFA/2*KACHU	A:A	T:T
BW22GS019624	KACHU//WBLL1*2/BRAMBLING*2/6/ROLF07*2/5/REH/HARE//2*BCN/3/CROC_1/AE.SQUARROSA (213)//PGO/4/HUITES*2/7/KUTZ//KFA/2*KACHU	A:A	T:T
BW22GS019625	KACHU//WBLL1*2/BRAMBLING*2/6/ROLF07*2/5/REH/HARE//2*BCN/3/CROC_1/AE.SQUARROSA (213)//PGO/4/HUITES*2/7/KUTZ//KFA/2*KACHU	A:A	T:T
BW22GS019626	KACHU//WBLL1*2/BRAMBLING*2/3/KACHU/KIRITATI/4/CIRO16/2*BORL14/5/KACHU//WBLL1*2/BRAMBLING*2/3/KACHU/KIRITATI	A:A	T:T

BW22GS019627	CHIBIA//PRLII/CM65531/3/MISR 2*2/4/QUAIU/5/PBW343*2/KUKUNA*2//FRTL/PIFED/6/CHIBIA//PRLII/CM65531/3/FISCAL/4/SUP152/7/2*MOKUE #1	A:A	T:T
BW22GS019628	BLOUK #1/MUNAL/3/WBLL1*2/SHAMA//BAJ #1/4/SUP152/BAJ #1/5/MOKUE #1/6/KACHU//WBLL1*2/BRAMBLING*2/3/KACHU/KIRITATI	A:A	T:T
BW22GS019629	BLOUK #1/MUNAL/3/WBLL1*2/SHAMA//BAJ #1/4/SUP152/BAJ #1/5/MOKUE #1/6/KACHU//WBLL1*2/BRAMBLING*2/3/KACHU/KIRITATI	A:A	T:T
BW22GS019630	BLOUK #1/MUNAL/3/WBLL1*2/SHAMA//BAJ #1/4/SUP152/BAJ #1/5/MOKUE #1/6/KACHU//WBLL1*2/BRAMBLING*2/3/KACHU/KIRITATI	A:A	T:T
BW22GS019631	SUP152/BLOUK #1/3/PRL/2*PASTOR*2//VORB/4/SUP152/BLOUK #1*2/5/BORL14*2//KFA/2*KACHU	A:A	T:T
BW22GS019632	SUP152/BLOUK #1/3/PRL/2*PASTOR*2//VORB/4/SUP152/BLOUK #1*2/5/BORL14*2//KFA/2*KACHU	A:A	C:T
BW22GS019633	BECARD/AKURI/3/KACHU//WBLL1*2/BRAMBLING/4/MUTUS/AKURI*2/5/MOKUE #1	A:A	T:T
BW22GS019634	KACHU//WBLL1*2/BRAMBLING/3/KACHU/KIRITATI/4/MOKUE #1/5/BORL14*2//MUNAL #1/FRANCOLIN #1	A:A	T:T
BW22GS019635	KACHU #1/3/T.DICOCCON PI94624/AE.SQUARROSA (409)//BCN/4/2*KACHU/5/MUTUS*2/TECUE #1/6/MUTUS*2/TECUE #1*2/7/MOKUE #1	A:A	T:T
BW22GS019636	KACHU #1/3/T.DICOCCON PI94624/AE.SQUARROSA (409)//BCN/4/2*KACHU/5/MUTUS*2/TECUE #1/6/MUTUS*2/TECUE #1*2/7/NELOKI*2//KACHU/KIRITATI	A:A	T:T
BW22GS019637	KACHU #1/3/T.DICOCCON PI94624/AE.SQUARROSA (409)//BCN/4/2*KACHU/5/MUTUS*2/TECUE #1/6/MUTUS*2/TECUE #1*2/7/NELOKI*2//KACHU/KIRITATI	A:A	T:T
BW22GS019638	KACHU #1/3/T.DICOCCON PI94624/AE.SQUARROSA (409)//BCN/4/2*KACHU/5/MUTUS*2/TECUE #1/6/MUTUS*2/TECUE #1*2/7/NELOKI*2//KACHU/KIRITATI	A:A	T:T
BW22GS019639	KACHU #1/3/T.DICOCCON PI94624/AE.SQUARROSA (409)//BCN/4/2*KACHU/5/MUTUS*2/TECUE #1/6/MUTUS*2/TECUE #1*2/7/NELOKI*2//KACHU/KIRITATI	A:A	T:T
BW22GS019640	SUP152/QUAIU #2//BECARD/QUAIU #1/7/ATTILA/3*BCN//BAV92/3/PASTOR/4/TACUPETO F2001*2/BRAMBLING/5/PAURAQ/6/KFA/2*KACHU/8/KFA/2*KACHU*2//MISR 1	A:A	T:T
BW22GS019641	KACHU #1/3/T.DICOCCON PI94624/AE.SQUARROSA (409)//BCN/4/2*KACHU/5/MUTUS*2/TECUE #1*2/7/ATTILA/3*BCN//BAV92/3/PASTOR/4/TACUPETO F2001*2/BRAMBLING/5/PAURAQ/6/KFA/2*KACHU	A:A	T:T

BW22GS019642	ATTILA*2/PBW65*2//KACHU/3/FRNCLN*2/TECUE #1/4/KUTZ*2//KFA/2*KACHU/5/BORL14*2//MUNAL #1/FRANCOLIN #1	A:A	T:T
BW22GS019643	ATTILA*2/PBW65*2//KACHU/3/FRNCLN*2/TECUE #1*2/6/WBLL1*2/KKTS//PASTOR/KUKUNA/3/KINGBIRD #1//INQALAB 91*2/TUKURU/5/KAUZ//ALTAR 84/AOS/3/MILAN/KAUZ/4/SAUAL	A:A	T:T
BW22GS019644	SUP152//WBLL1*2/BRAMBLING*2/3/KSW/SAUAL//SAUAL/4/BORL14*2//KFA/2*KACHU/5/BORL14*2//KFA/2*KACHU	A:A	T:T
BW22GS019645	SUP152//WBLL1*2/BRAMBLING*2/3/KSW/SAUAL//SAUAL*2/4/PRL/2*PASTOR//KACHU	A:A	T:T
BW22GS019646	SUP152//WBLL1*2/BRAMBLING*2/3/KSW/SAUAL//SAUAL*2/4/CIRO16/2*BORL14	NA	T:T
BW22GS019647	BECARD//ND643/2*WBLL1*2/3/KSW/SAUAL//SAUAL*2/4/KFA/2*KACHU*2//SUP152	NA	T:T

Table S18 - Target-specific amplification efficiencies of the splicing variants *Pm4b_V1* and *Pm4b_V2* and the reference genes used for RT-qPCR in this study.

gene / Target	gene ID	position	primer	amplicon length bp	reference	efficiency (%)	Slope	r2 of calibration curve
Pm4_V1		Exon 5-6	F: TAGGTTGGAGAGATCACAACGA (GH414)<br>R: CTGAGGTAGAGGAGGCAACTT (GH415)	179	Sanchéz-Martin et al, 2021	100.75	-3.304	0.9983
Pm4_V2		Exon 5-7	F: AGAGTGCAGAGACTTCAATCCA (GH377)<br>R: TTCTTCGTACCCAGCAGGTC (GH417)	159	Sanchéz-Martin et al, 2021	83.44	-3.795	0.9918
ADP	TraesCS3B01G368600, TraesCS3D01G330500 (TA.2291)	Exon 2	F: TCTCATGGTTGGTCTCGATG (GH094)<br>R: GGATGGTGGTGACGATCTCT (GH095)	80	Giménez et al, as referenced by Sanchéz-Martin et al, 2021	98.92	-3.348	0.9985
ZFL	TraesCS3D01G432800, TraesCS3A01G440000	Exon 1	F: CAGGCATCTCACTGGAGACT (GH105)<br>R: TGGCATCTCTCTTGCTTCTG (GH106)	79	Sanchéz-Martin et al, 2021	98.6	-3.356	0.9933

Supplementary references

Anh, V., Y. Inoue, S. Asuke, T. T. P. Vy, N. T. Anh, S. Wang, I. Chuma & Y. Tosa (2018) Rmg8 and Rmg7, wheat genes for resistance to the wheat blast fungus, recognize the same avirulence gene AVR-Rmg8. *Molecular plant pathology,* 19**,** 1252-1256.

Anh, V. L., N. T. Anh, A. G. Tagle, T. T. P. Vy, Y. Inoue, S. Takumi, I. Chuma & Y. Tosa (2015) Rmg8, a New Gene for Resistance to Triticum Isolates of Pyricularia oryzae in Hexaploid Wheat. *Phytopathology,* 105**,** 1568-1572.

Arora, S., A. Steed, R. Goddard, K. Gaurav, T. O'Hara, A. Schoen, N. Rawat, A. F. Elkot, A. V. Korolev & C. Chinoy (2023) A wheat kinase and immune receptor form host-specificity barriers against the blast fungus. *Nature Plants***,** 1-8.

Briggle, L. (1966) Transfer of resistance to Erysiphe graminis f. sp. tritici from Khapli Emmer and Yuma Durum to Hexaploid Wheat 1. *Crop Science,* 6**,** 459-461.

Doussinault, G., A. Delibes, R. Sanchez-Monge & F. Garcia-Olmedo (1983) Transfer of a dominant gene for resistance to eyespot disease from a wild grass to hexaploid wheat. *Nature,* 303**,** 698-700.

Helguera, M., I. Khan, J. Kolmer, D. Lijavetzky, L. Zhong-Qi & J. Dubcovsky (2003) PCR assays for the Lr37-Yr17-Sr38 cluster of rust resistance genes and their use to develop isogenic hard red spring wheat lines. *Crop Science,* 43**,** 1839-1847.

McIntosh, R. & F. G. Bennett (1979) Cytogenetical studies in wheat. IX. Monosomic analyses, telocentric mapping and linkage relationships of genes Sr21, Pm4 and Mle. *Australian Journal of Biological Sciences,* 32**,** 115-126.

Nga, N., V. Hau & Y. Tosa (2009) Identification of genes for resistance to a Digitaria isolate of Magnaporthe grisea in common wheat cultivars. *Genome,* 52**,** 801-809.

Sánchez-Martín, J., V. Widrig, G. Herren, T. Wicker, H. Zbinden, J. Gronnier, L. Spörri, C. R. Praz, M. Heuberger, M. C. Kolodziej, J. Isaksson, B. Steuernagel, M. Karafiátová, J. Doležel, C. Zipfel & B. Keller (2021) Wheat Pm4 resistance to powdery mildew is controlled by alternative splice variants encoding chimeric proteins. *Nature Plants,* 7**,** 327-341.

Tagle, A. G., I. Chuma & Y. Tosa (2015) Rmg7, a New Gene for Resistance to Triticum Isolates of Pyricularia oryzae Identified in Tetraploid Wheat. *Phytopathology,* 105**,** 495-9.

Takabayashi, N., Y. Tosa, H. S. Oh & S. Mayama (2002) A gene-for-gene relationship underlying the species-specific parasitism of Avena/Triticum isolates of Magnaporthe grisea on wheat cultivars. *Phytopathology,* 92**,** 1182-1188.

VSN, I. 2022. Genstat *for Windows* 22nd Editiion. VSN International, Hemel Hempstead, UK.

Vy, T. T. P., G.-S. Hyon, N. T. T. Nga, Y. Inoue, I. Chuma & Y. Tosa (2014) Genetic analysis of host–pathogen incompatibility between Lolium isolates of Pyriculariaoryzae and wheat. *Journal of general plant pathology,* 80**,** 59-65.

Walkowiak, S., L. Gao, C. Monat, G. Haberer, M. T. Kassa, J. Brinton, R. H. Ramirez-Gonzalez, M. C. Kolodziej, E. Delorean & D. Thambugala (2020) Multiple wheat genomes reveal global variation in modern breeding. *Nature,* 588**,** 277-283.

Wang, S., S. Asuke, T. T. P. Vy, Y. Inoue, I. Chuma, J. Win, K. Kato & Y. Tosa (2018) A new resistance gene in combination with Rmg8 confers strong resistance against Triticum isolates of Pyricularia oryzae in a common wheat landrace. *Phytopathology,* 108**,** 1299-1306.

Zhan, S. W., S. Mayama & Y. Tosa (2008) Identification of two genes for resistance to Triticum isolates of Magnaporthe oryzae in wheat. *Genome,* 51**,** 216-21.
